# Artemisinin analog SM934 alleviates epithelial barrier dysfunction *via* inhibiting apoptosis and caspase-1-mediated pyroptosis in experimental colitis

**DOI:** 10.3389/fphar.2022.849014

**Published:** 2022-09-01

**Authors:** Meijuan Shao, Yuxi Yan, Fenghua Zhu, Xiaoqian Yang, Qing Qi, Fangming Yang, Tingting Hao, Zemin Lin, Peilan He, Yu Zhou, Wei Tang, Shijun He, Jianping Zuo

**Affiliations:** ^1^ Laboratory of Immunology and Virology, Shanghai University of Traditional Chinese Medicine, Shanghai, China; ^2^ Laboratory of Immunopharmacology, State Key Laboratory of Drug Research, Shanghai Institute of Materia Medica, Chinese Academy of Sciences, Shanghai, China; ^3^ University of Chinese Academy of Sciences, Beijing, China; ^4^ Laboratory of Anti-inflammation, Shanghai Institute of Materia Medica, Chinese Academy of Sciences, Shanghai, China

**Keywords:** SM934, colitis, intestinal epithelial barrier, apoptosis, pyroptosis, NLRP3/caspase-1/GSDMD, MAPK /NF-kB

## Abstract

Intestinal barrier disruption due to the intestinal epithelial cells’ (IECs) death is one of the critical pathological features of inflammatory bowel diseases (IBDs). SM934, an artemisinin analog, has previously been proven to ameliorate colitis induced by dextran sulfate sodium (DSS) in mice by suppressing inflammation response. In this study, we investigated the protective effects of SM934 on the epithelial barrier and the underlying mechanism in trinitrobenzene sulfonic acid (TNBS)-induced colitis mice. We demonstrated that SM934 restored the body weight and colon length, and improved the intestine pathology. Furthermore, SM934 treatment preserved the intestinal barrier function *via* decreasing the intestinal permeability, maintaining epithelial tight junction (TJ) protein expressions, and preventing apoptosis of epithelial cells, which were observed both in the colon tissue and the tumor necrosis factor-α (TNF-α)-induced human colonic epithelial cell line HT-29. Specifically, SM934 reduced the pyroptosis of IECs exposed to pathogenic signaling and inhibited pyroptosis-related factors such as NOD-like receptor family pyrin domain containing 3 (NLRP3), adapter apoptosis-associated speck-like protein (ASC), cysteine protease-1 (caspase-1), gasdermin (GSDMD), interleukin-18 (IL-18), and high-mobility group box 1 (HMGB1) both in colon tissue and lipopolysaccharide (LPS) and adenosine triphosphate (ATP) co-stimulated HT-29 cells *in vitro*. Moreover, SM934 interdicted pyroptosis *via* blocking the transduction of mitogen-activated protein kinase (MAPK) and nuclear factor-kB (NF-kB) signaling pathways. In conclusion, SM934 protected TNBS-induced colitis against intestinal barrier disruption by inhibiting the apoptosis and pyroptosis of epithelial cells *via* the NLRP3/NF-κB/MAPK signal axis, and intestinal barrier protection in company with an anti-inflammatory strategy might yield greater benefits in IBD treatment.

## Introduction

Crohn’s disease (CD) and ulcerative colitis (UC) are two major subtypes of inflammation bowel disease (IBD), with a lifetime risk of recurrence inflammation in the gastrointestinal tract. It is clinically characterized by abdominal pain, diarrhea, and body weight loss (Hisamatsu et al., 2013). During the pathogenesis, the impaired intestinal epithelial barrier function with increased permeability is caused by the mediators of pathogenic microorganisms and then recruits large numbers of pro-inflammatory molecules, which in turn leads to intestinal mucosa barrier injury and plays a crucial role in the progression of IBD (Mankertz and Schulzke, 2007; Peterson and Artis, 2014).

It is well known that the intestinal epithelial barrier is essential to maintain gastrointestinal homeostasis depending on the functions of intestinal epithelial cells (IECs), which is regulated by the epithelial apical junctional complex (AJC) consisting of adherent junction (AJ) and tight junction (TJ) proteins (McCarter et al., 2010; Lei-Leston et al., 2017). Moreover, E-cadherin, a type I cadherin cell adhesion molecule, is the major TJ of zonula occludens-1 (ZO-1), occludin, and claudins that are crucial in maintaining the intestinal barrier (Mehta et al., 2015). Furthermore, the report demonstrated that E-cadherin in patients with CD showed a remarkable reduction and reflected the degree of inflammation in the intestine (Meir et al., 2020). On the other hand, the decreasing expression of occludin, ZO-1, and claudin proteins leads to the loss of epithelial barrier integrity and permeability improving in the intestinal tissues of CD (Pochard et al., 2021; Song et al., 2022). Furthermore, apoptosis regulates the replacement rate of the epithelial cells, and an increase in epithelial cell apoptosis in colitis disrupts the mucosal epithelial barrier (Cai et al., 2018; Xiong et al., 2019). However, an aberrantly high rate of IEC apoptosis in IBD leads to a positive feedback cycle of epithelial barrier disruption but does not cause an inflammation response (Bayrer et al., 2018; Zhang L. et al., 2021).

At present, the molecular and pathophysiological mechanisms by which the intestinal barrier function is regulated by IECs’ death trigger an inflammatory cascade to clear the invading bacteria during the development of IBD remain elusive. Accumulating evidence indicates that microbiota-driven activation of inflammatory responses and pathological immune activation to further progressive tissue damage through the release of pro-inflammatory mediators from dying IECs is commonly associated with inflammatory pyroptosis (Wang et al., 2021). It is well known that pyroptosis is defined as caspase-1-dependent programmed cell death that is activated by the NOD-, LRR-, and pyrin domain-containing 3 (NLRP3) inflammasomes, which is a cytosolic protein complex composed of NLRP3, the adapter apoptosis-associated speck-like protein (ASC) and procaspase-1 (Li et al., 2020). In addition, activation of NLRP3 facilitates pro-caspase-1 into cleaved caspase-1 formation. Furthermore, the pyroptosis effector gasdermin (GSDMD) is cleaved by activation of caspase-1 into an N-terminal domain (GSDMD-N) and a C-terminal domain (GSDMD-C). Then, mature GSDMD causes features like cell membrane-located pore formation, cell swelling, and rupture, which ultimately leads to cell pyroptosis, as well as the following releases of pro-inflammatory cytokines interleukin-1β (IL-1β) and interleukin-18 (IL-18) that occur in intestinal tissues to modulate the epithelial barrier function and mediate the inflammatory response in the progression of IBD (Kim and Kim, 2018; Liu et al., 2018). In addition, Nod-like receptors trigger inflammation. NLRP3 can be initiated by transcription factor nuclear factor-kappa B (NF-κB) as the first step of pyroptosis, and also, NF-κB can be activated by the mitogen-activated protein kinase (MAPK) pathway; both of them regulate the balance between mucosal homeostasis and inflammation in colitis (Liang et al., 2020; Lan et al., 2021).

Artemisinin has been isolated from *Artemisia annua* of a Chinese medicinal plant, as an active principle against malaria, and has shown good immunosuppressive and anti-inflammatory effects on autoimmune diseases, including inflammatory bowel disease, osteoarthritis, erythematosus, and arthritis (Hou et al., 2011; Zhao et al., 2017; Kim et al., 2019; Sun et al., 2020). In addition, SM934 is a novel water-soluble artemisinin analog named β-aminoarteether maleate; it has been demonstrated that it possesses strong immunosuppressive activities and fewer side effects in our laboratory studies earlier (Hou et al., 2009). Previous studies showed that SM934 protected rat experimental membranous nephropathy mice against glomerulonephritis and renal fibrosis (Li et al., 2015). In our latest studies, we reported that SM934 ameliorated DSS-induced ulcerative colitis, exerted anti-inflammatory functions on neutrophils and macrophages (Yan et al., 2018), and alleviated dry eye disease in rodent models (Yang et al., 2021). Recently, studies reported that the artemisinin derivative such as artesunate ameliorated colitis by protecting the intestinal barrier and inhibiting apoptosis (Yin et al., 2020). Dihydroartemisinin prevented colitis through inhibition of the activation of the NLRP3 inflammasome, and NF-κB and MAPK signaling (Liang et al., 2020). However, the underlying mechanisms of SM934 against intestinal barrier disruption based on cell death in the treatment of colitis remain unclear and need further exploration.

In this study, we aimed to investigate the effects of SM934 on the trinitrobenzene sulfonic acid (TNBS)-induced murine colitis model. It has been demonstrated that SM934 treatment significantly reduces the severity of the disease. Moreover, we found that SM934 repaired intestinal barrier damage and inhibited IEC apoptosis, as well as blocked caspase-1-mediated pyroptosis *via* NlRP3/NF-κB/MAPK signaling pathways *in vivo* and *in vitro*. In summary, this might provide an experimental basis for understanding the therapeutics and mechanism of SM934 for IBD progression.

## Materials and methods

### Materials and animals

SM934 (No. 2011012-4), β-aminoarteether maleate, was synthesized and provided by Zhejiang Puluokangyu Natural Medicine Co., Ltd. (Zhejiang, China), and dissolved in sterile double distilled water (ddH_2_O) as a stock solution. Prednisone acetate (PNS, No. 018161105) was purchased from Shanghai Pharmaceutical Co., Ltd. (Shanghai, China). 2, 4, 6-Trinitro-benzene sulfonic acid (5% TNBS in H_2_O, wt/vol) was purchased from Sigma (P2297, Sigma-Aldrich, St. Louis, MO, United States).

Female C57BL/6 mice (6–8 weeks, 18–21 g) were obtained from Shanghai Lingchang Biotechnology Co., Ltd. (Shanghai, China). The mice were housed in an air-conditioned room with the laboratory temperature under a 12-h light/dark cycle (22 ± 1°C), provided with a standard chow diet and water *ad libitum*, and allowed to acclimate for 1 week before the experiments were performed. All experimental protocols were guided in accordance with the approval of the Institutional Animal Care and Use Committee of Shanghai Institute of Materia Medica (SIMM, Shanghai, China).

### Trinitrobenzene sulfonic acid-induced colitis in mice and treatment

The experimental colitis mouse model was established by intrarectal administration of TNBS together with ethanol and was performed, as previously described (Wirtz et al., 2017). In brief, mice were randomly divided into four groups (*n* = 9): the normal group, vehicle group (only TNBS), the treatment group of the SM934 group (TNBS and SM934), and the PNS (TNBS and PNS ) group, according to body weight. From day 0, other than the normal group, the vehicle group, SM934 group, and PNS group mice were given intrarectal (i.r.) administration with 2.5% TNBS containing 50% ethanol. The mice of the SM934 group and PNS group were treated with SM934 10 mg/kg and PNS 2 mg/kg daily from day 3 to day 7. The normal group and vehicle group mice received ddH_2_O orally daily. On day 7, mice were killed, and their colons were gathered and photographed. The colon tissues were collected for histopathological analysis with hematoxylin and eosin (H&E) staining; other colon tissues were used for qRT-polymerase chain reaction (qRT-PCR) analysis, Western blot, and immunohistochemistry (IHC) experiments as follows.

### Histological analyses

The colonic tissues isolated from mice were fixed in 10% formalin and embedded in paraffin. Tissue sections of the colon (3 μm) were stained with hematoxylin and eosin (H&E) for evaluating the pathological damage of colitis. Histological scoring was performed by three professional gastrointestinal pathologists (Drug Safety Evaluation and Research Center, SIMM, CAS) according to reported criteria using a previously described method (Santucci et al., 2003). Briefly, the scoring criteria was graded as: 0, no signs of inflammation; 1, very low level of inflammation; 2, moderate inflammation with positive leukocyte infiltration; 3, high level of positive leukocyte infiltration, high vascular density, and thickening of the colon wall; and 4, transmural inflammatory and abundant leukocyte infiltration, loss of goblet cells, and thickening of the colon wall. The colon sections were examined using a microscope (Nikon Eclipse Ci, Tokyo, Japan). The images were acquired with an imaging system (digital sight DS-FI2, Eclipse C2, Nikon, Tokyo, Japan).

### Intestinal epithelial barrier permeability assay

Intestinal permeability was evaluated using the intestinal barrier function, as previously described (Li et al., 2018). Briefly, the mice were gavaged with FITC-CM-dextran (400 mg/kg body weight, NO. 68059, Sigma-Aldrich, St. Louis, MO, United States). Then, 4 h later, the blood was collected, and the FITC–dextran levels of the serum were measured at the excitation 488 nm and emission 525 nm using a fluorescence spectrophotometer (SpectraMax M5, Molecular Devices Corporation, Sunnyvale, CA).

*In vitro*, Caco2 cells were seeded in a 24-well Transwell^®^ polycarbonate membrane insert (No. 3415, 3-μm pore size, Corning, New York, United States) until monolayers were formed. The cells were treated with or without TNF-α (100 ng/ml) in the absence or presence of SM934 (10 μM) for 24 h. The monolayers were washed, and FITC-dextran solution (1 mg/ml) was added to the apical chambers and incubated at 37°C for 2 h. Then, the medium of the basal chamber was detected with a fluorescence spectrophotometer.

### Transepithelial electrical resistance (TEER)

To evaluate the intestinal barrier function, intestinal monolayer cells of Caco-2 cells were formed in a Transwell^®^ polycarbonate membrane insert. The electrical resistance was measured with a Millicell^®^ ERS-2 Voltohmmeter (Merck, Darmstadt, Germany).

### Immunohistochemistry and immunofluorescence

The colon sections were deparaffinized in xylene and rehydrated into water through graded alcohol. For immunohistochemistry, the sections were blocked with 5% BSA and incubated with the anti-Ki67 antibody (1:100, ab1667, Abcam, Cambridge, United Kingdom) and anti-E-cadherin (1:100, 3195S, Cell Signaling Technology, Danvers, MA, United States) as the primary antibodies to detect the antigen overnight at 4 C. Then, the primary antibody was combined with the horseradish peroxidase (HRP)-conjugated goat anti-rabbit IgG, and the signals were detected using diaminobenzidine. Images were acquired using NanoZoomer 2.0-HT microdissection systems (Bensons, Japan) and analyzed by ImageJ software (National Institutes of Health, Bethesda, MD, United States). For immunofluorescence, the colon tissue sections were incubated with primary antibodies anti-cleaved caspase-3 (1:100, 9661S, CST, Danvers, MA, United States) and anti-caspase-1 (1:100, 8932S, Abclonal, Wuhan, China) overnight at 4°C. The sections were incubated with the Alexa Fluor^®^ 488-conjugated anti-rabbit secondary antibodies (1:400, GB35303, Servicebio, Wuhan, China) at room temperature and then counterstained with DAPI (ab104139, Abcam, Cambridge, United Kingdom) to stain the nuclei. Images were collected on a microscope imaging system (Nikon DS-U3, Tokyo, Japan).

### TUNEL assay

TUNEL staining with an *in situ* cell death detection kit purchased from (G1501, Servicebio, Wuhan, China) was performed to detect death cells in colon sections, according to the manufacturer’s instructions. Fluorescence microscopy was performed (Nikon DS-U3, Tokyo, Japan), and the images were captured.

### RNA extraction and real-time PCR

Total RNA was isolated from the colon tissues of mice using the RNAsimple total RNA kit (Tiangen, Beijing, China), and reverse transcription was performed using the Hifair™ 1st Strand cDNA Synthesis SuperMix (Yeasen, Shanghai, China). Real-time PCR was carried out with the SYBR^®^ Green Real-time PCR Master Mix (Yeasen, Shanghai, China) and analyzed with the Applied Biosystems 7500 Fast Real-Time PCR System (Foster city, CA, United States). The mRNA expression level of the target gene was normalized to GAPDH in accordance with the 2^−ΔΔCt^ method. The sequences of the qPCR primers are listed as follows: mouse bcl-2 forward, 5′-GTC​GCT​ACC​GTC​GTG​ACT​TC-3′, bcl-2 reverse, 5′- CAG​ACA​TGC​ACC​TAC​CCA​GC-3'; mouse bax forward, 5′- TGA​AGA​CAG​GGG​CCT​TTT​TG-3′, bax reverse, 5′-AAT​TCG​CCG​GAG​ACA​CTC​G-3'; mouse caspase-9 forward, 5′-TCC​TGG​TAC​ATC​GAG​ACC​TTG-3′, caspase-9 reverse, 5′-AAG​TCC​CTT​TCG​CAG​AAA​CAG-3'; mouse caspase-3 forward, 5′- ATG​GAG​AAC​AAC​AAA​ACC​TCA​GT-3′, caspase-3 reverse, 5′- TTG​CTC​CCA​TGT​ATG​GTC​TTT​AC-3'; mouse NLRP3 forward, 5′-ATT​ACC​CGC​CCG​AGA​AAG​G-3′, NLRP3 reverse, 5′-TCG​CAG​CAA​AGA​TCC​ACA​CAG-3'; mouse ASC forward, 5′-CTT​GTC​AGG​GGA​TGA​ACT​CAA​AA-3′, ASC reverse, 5′-GCC​ATA​CGA​CTC​CAG​ATA​GTA​GC-3'; mouse caspase-1 forward, 5′-ACA​AGG​CAC​GGG​ACC​TAT​G-3′, caspase-1 reverse, 5′-TCC​CAG​TCA​GTC​CTG​GAA​ATG-3'; mouse GSDMD forward, 5′-CCA​TCG​GCC​TTT​GAG​AAA​GTG-3′, GSDMD reverse, 5′-ACA​CAT​GAA​TAA​CGG​GGT​TTC​C-3'; mouse IL-18 forward, 5′-GAC​TCT​TGC​GTC​AAC​TTC​AAG​G-3′, IL-18 reverse, 5′-CAG​GCT​GTC​TTT​TGT​CAA​CGA​T-3'; mouse HMGB1 forward, 5′-GGC​GAG​CAT​CCT​GGC​TTA​TC-3′, HMGB1 reverse, 5′-GGC​TGC​TTG​TCA​TCT​GCT​G-3'; and mouse GAPDH forward, 5′- AGG​TCG​GTG​TGA​ACG​GAT​TTG-3′, GAPDH reverse, 5′- TGT​AGA​CCA​TGT​AGT​TGA​GGT​CA-3'.

### Cell cultures

HT-29 and Caco-2 cells were obtained from the American Type Culture Collection (ATCC, Manassas, VA, United States). HT-29 cells were cultured with McCoy’s 5a medium (16600082, Gibco, CA, United States), and Caco-2 cells were cultured with DMEM F12 medium (11320033, Gibco, CA, United States), supplemented with 100 U/mL ostreptomycin and 100 U/mL of penicillin containing 10% fetal bovine serum (FBS, AHW87326, HyClone, UT, United States) in a humidified incubator consisting of a 5% CO_2_ atmosphere at 37 C. In *in vitro* experiments, HT-29 cells were pre-incubated with SM934 (10 μM) for 1 h. For simulating the intestinal barrier function and apoptosis in cell models, HT-29 cells were exposed to with or without 100 ng/ml tumor necrosis factor-α (TNF-α) (041525, PeproTech, Rocky Hill, NJ, United States) in the absence or presence of SM934 (10 μM) for 24 h or 72 h. For simulating pyroptosis-related inflammatory pathways in cell models, HT-29 cells were stimulated with or without 1 μg/ml lipopolysaccharides (LPS) (L2880, Sigma-Aldrich, St. Louis, MO, United States) and 5 mM adenosine triphosphate (ATP) (A6419-1G, Sigma-Aldrich, St. Louis, MO, United States) or LPS (1 μg/ml) in the absence or presence of SM934 (10 μM) for 12 h. Cells were harvested and then applied to immunofluorescence and western blot assays, as described as follows.

### Immunofluorescence cytochemistry

HT-29 cells adherently cultured on coverslips were fixed with 4% paraformaldehyde for 20 min. For intracellular antigen, the cells were permeabilized with Triton X-100 for 10 min, while the step was not needed for the detection of the junction proteins. After that, all cells were blocked with 5% BSA for 60 min and then incubated with anti-E-cadherin (1:200, 3195S, CST, Danvers, MA, United States), ZO-1 (1:200, 21773-1-AP , Proteintech, Chicago, United States), anti-cleaved caspase-3 (1:100, 9661S, CST, Danvers, MA, United States), anti-NLRP3 (1:100, A5652, ABclonal, Wuhan, China), anti-caspase-1 (1:100, A0964, ABclonal, Wuhan, China), and anti-GSDMD (1:100, 303922, Signalway Antibody, Maryland, United States) overnight at 4 C. The Alexa Fluor^®^ 488-conjugated anti-rabbit secondary antibodies (1:400, ab50077, Abcam, Cambridge, United Kingdom) were added for 1 h. The cells were counterstained with DAPI for a while. Finally, images were captured using a Leica TCS SPS CFSMP microscope (Solms, Germany).

### Western blot analysis

The protein samples of colon tissues and the cells were lysed using SDS buffer with the proteinase inhibitor. The protein concentration was quantified with the BCA Protein Assay Kit (Thermo, United States). The protein sample was separated and transferred onto NC membranes and blocked with 5% BCA for 1 h at RT. Next, the primary antibodies against E-cadherin (1:1,000, 3195S, CST, Danvers, MA, United States), β-catenin (1:1,000, 8480S, CST, Danvers, MA, United States), occludin (1:1,000, ab67161, Abcam, Cambridge, United Kingdom), ZO-1 (1:1,000, 61-7,300, Proteintech, Rosemont, United States), claudin-2 (1:1,000, ab53032, Abcam, Cambridge, United Kingdom), claudin-1 (1:1,000, ab15098, Abcam, Cambridge, United Kingdom), Bcl-2 (1:1,000, 3498S, CST, Danvers, MA, United States), Bax (1:1,000, 2772S, CST, Danvers, MA, United States), cleaved-caspase-9 (1:1,000, 9509S, CST, Danvers, MA, United States), cleaved-caspase-3 (1:1,000, 9661S, CST, Danvers, MA, United States), cleaved caspase-1 (1:1,000, 8932S, CST, Danvers, MA, United States), NLRP3 (1:1,000, A5652, ABclonal, Wuhan, China), ASC/TMS1 (E1E3I) rabbit mAb (1:1,000, 13833S, CST, Danvers, MA, United States), caspase-1 (1:1,000, A0964, ABclonal, Wuhan, China), GSDMD (1:1,000, 30392-2, Signalway Antibody, California, United States), IL-18 (1:1,000, 32166, Signalway Antibody, California, United States), HMGB1 (1:1,000, 6893S, CST, Danvers, MA, United States), Phospho-c-Jun N-terminal kinase (p-JNK) antibody (1:1,000, 9251S, CST, Danvers, MA, United States), JNK antibody (1:1,000, 9252S, CST, Danvers, MA, United States), NF-κB p65 (C22B4) rabbit mAb (1:1,000, 8242SL, CST, Danvers, MA, United States), phospho-NF-кB p65 (Ser536) (93H1) rabbit mAb (1:1,000, 3033L, CST, Danvers, MA, United States), p38 MAPK antibody (1:1,000, 9,212, CST, Danvers, MA, United States), P-p38 MAPK (T180/Y182) rabbit mAb (1:1,000, 9211S, CST, Danvers, MA, United States), phospho-extracellular signal-regulated kinase (Erk1/2) rabbit mAb (1:1,000, 4377S, CST, Danvers, MA, United States), and p44/42 MAPK (Erk1/2) (137F5) rabbit mAb (1:1,000, 4695S, CST, Danvers, MA, United States) were incubated overnight at 4°C. The secondary antibodies, HRP-conjugated anti-rabbit IgG (1:20,000, 1706515, Bio-Rad, Richmond, CA, United States) and HRP-conjugated monoclonal mouse anti-GAPDH (1:20,000, KC-5G5, KangChen, Shanghai, China) were incubated at RT for 1 h. The obtained protein bands were captured with the ECL system (Amersham Bioscience, Buckinghamshire, United Kingdom) with a classic autoradiography film system and Image Kwile Quant (Kindle Biosciences, LLC, United States).

### Statistics analysis

All data were expressed as mean ± SEM. Statistical analysis was performed with GraphPad Prism 7.0 (GraphPad Software, San Diego, CA, United States) software. Multiple group comparisons used one-way analysis of variance (ANOVA) with Dunnett’s *post hoc* test, and two groups were carried out using a *t*-test. *p <* 0.05 were considered significant.

## Results

### SM934 ameliorated the experimental symptoms of trinitrobenzene sulfonic acid-induced colitis in mice.

**FIGURE 1:**
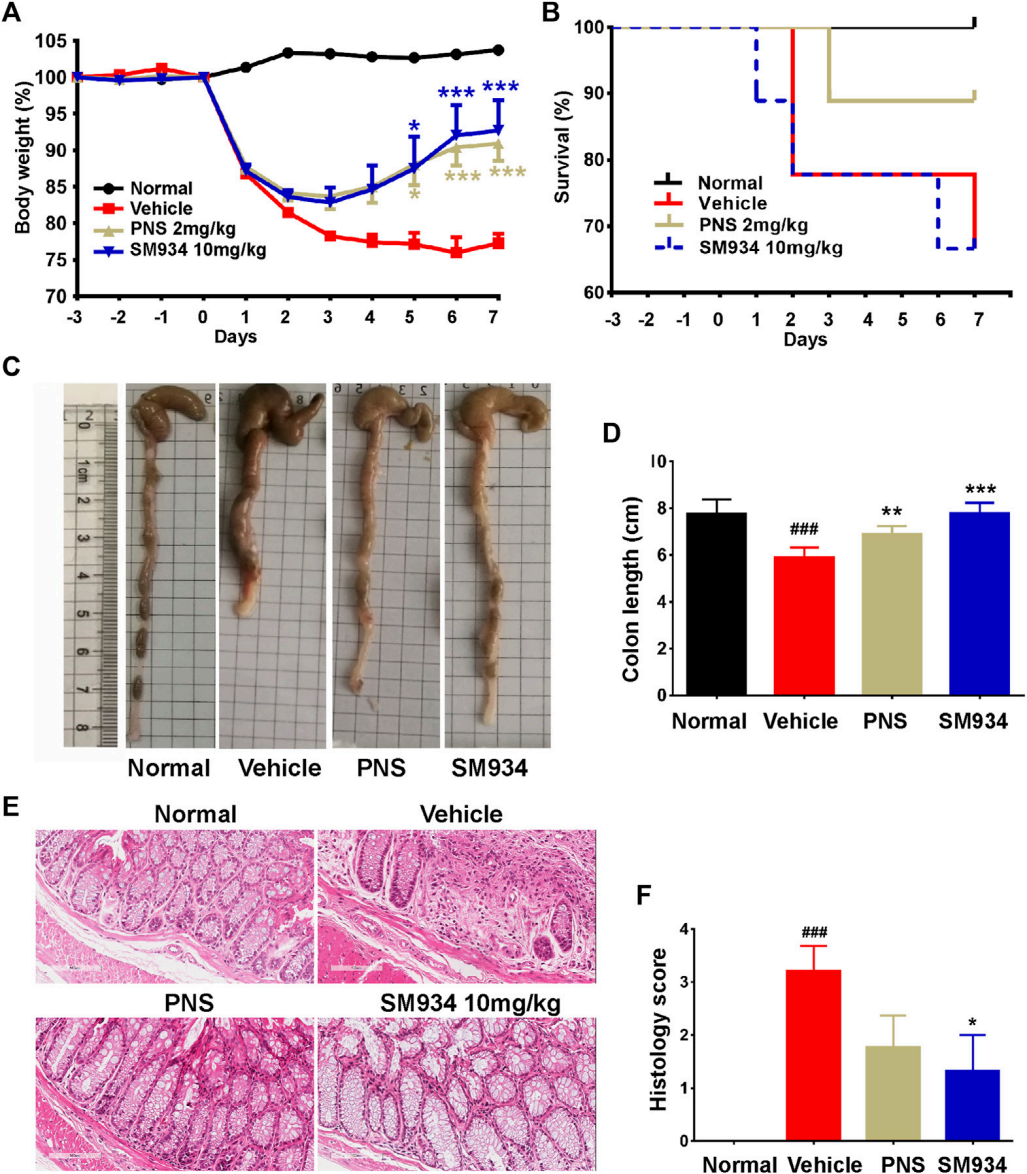
Effects of SM934 on TNBS-induced colitis in mice. **(A)** Body weight was monitored daily. **(B)** Survival curve. **(C)** Representative image of colons. **(D)** Average colon length of all groups. **(E)** Representative images of H&E-stained colon sections in mice (scale bars, 400 µm). **(F)** Histological score of mice in each group. Data are presented as means ± SEM (n = 9 mice/group). ^###^ *p* < 0.001 vs*.* normal group; *p < 0.05, **p < 0.01, and ***p < 0.001 vs*.* vehicle group.

In order to determine the therapeutic effect of SM934 on colitis. In this study, we investigated the effect of SM934 on TNBS-induced colitis in mice, which is a colitis model that directs barrier destruction with clinical symptoms similar to human CD (Wirtz et al., 2017). Here, mice were intrarectally administered with 2.5% TNBS containing 50% ethanol and treated with oral administration of SM934 (10 mg/kg) and PNS (2 mg/kg). The result showed obvious body weight loss in TNBS-treated mice, which directly reflected the physiological status of the colitis mice. As expected, SM934 and positive drug PNS effectively reversed the symptoms of colitis in mice (Figure 1A). Unexpectedly, the survival rate in SM934-treated mice had no visible difference compared with the vehicle group. In contrast, PNS-treated mice showed a decreased death rate after TNBS-challenged mice (Figure 1B), which is a class of glucocorticoids that has been widely used for autoimmune inflammatory disease (Lamb et al., 2019). Hence, we continued to observe the pivotal indicators both of colon length and pathological results at the end of the experiment to evaluate the severity of TNBS-induced colitis. In fact, the colon lengths were much shorter in the vehicle group mice than those in the normal group. Conversely, treatment with SM934 and PNS had a marked improvement in repairing the colon lengths in TNBS-induced colitis mice (Figures 1C,D). Meanwhile, H&E staining evaluated the intestinal structure and analyzed histological scores of surface epithelium erosion, thickened intestinal wall, crypt integrity, mucosal injury, and inflammatory cell infiltration. Interestingly, the pathological symptoms of SM934 treatment colitis mice presented notable remission, even better than the PNS group (Figure 1D). Taken together, our results indicated that SM934 significantly inhibited the symptoms’ severity and development in the TNBS-induced colitis.

### SM934 restored intestinal barrier function and reduced abnormal epithelial proliferation in trinitrobenzene sulfonic acid-induced colitis

**FIGURE 2:**
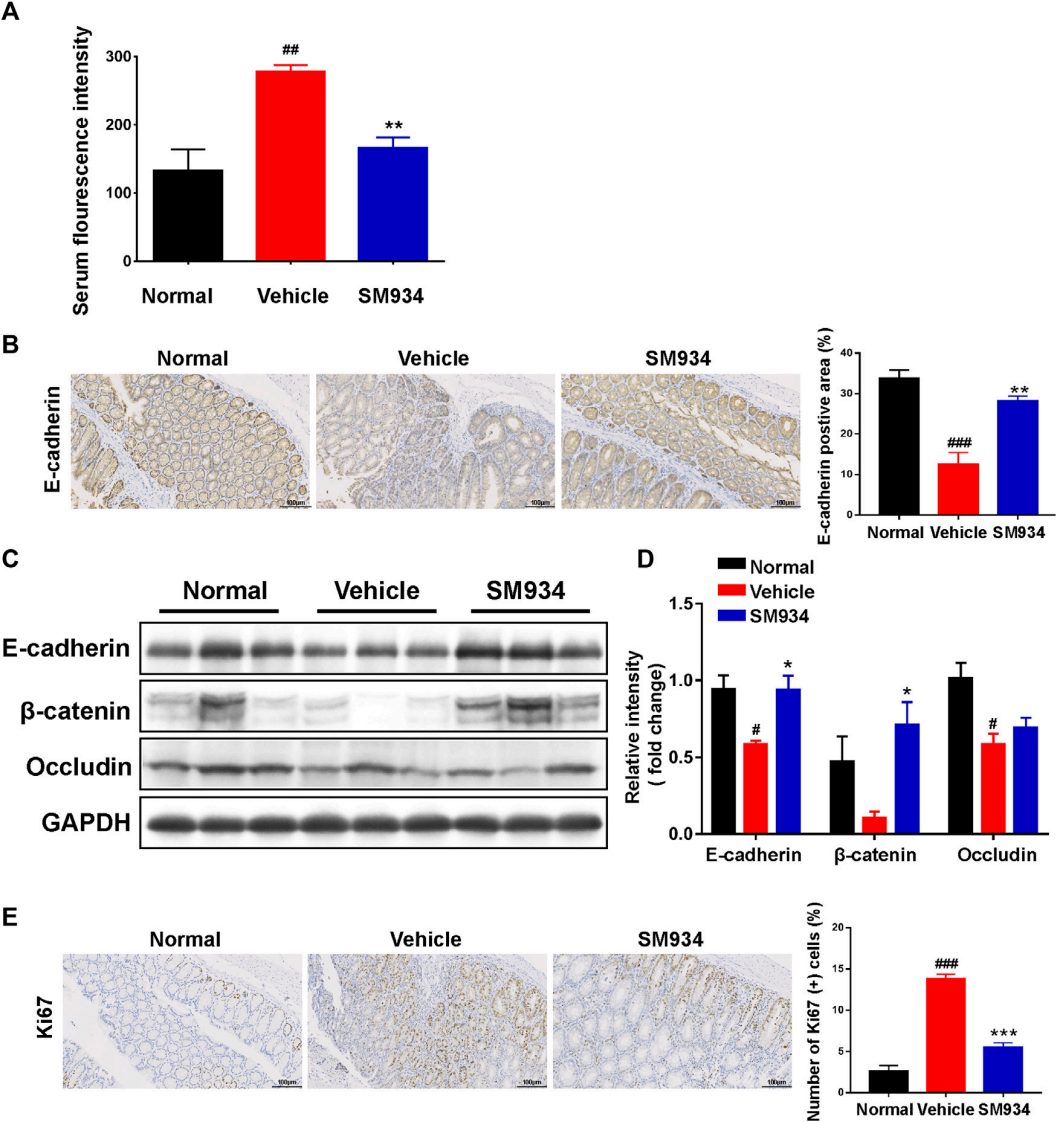
SM934 restored the intestinal barrier and reduced abnormal epithelial proliferation in TNBS-induced colitis mice. **(A)** Serum FITC-dextran fluorescence intensity was used to observe the intestinal permeability (*n* = 4). **(B)** Representative images of the positive staining area of E-cadherin with immunohistochemical staining (scare bar, 100 µm), right; the positive area was analyzed (left). **(C)** Western blotting assayed the expressions of E-cadherin/β-catenin/occludin in colon tissue. **(D)** Relative intensity of protein to GAPDH. Data are shown as means ± SEM (*n* = 3). **(E)** Representative images of colon sections stained with the proliferation marker Ki67 (Scale bar, 100 µm), right; the number of Ki67-positive cells was counted, left. ^#^ *p* < 0.05; ^###^ *p* < 0.001 vs*.* normal group; *p < 0.05, **p < 0.01, and ***p < 0.001 vs*.* vehicle group.

Accumulating evidence indicates that intestinal barrier dysfunction is manifested as the increased intestinal permeability and loss of TJ proteins in patients or mice with IBD. In addition, abnormal intestinal epithelial cell proliferation is associated with the integrity of the intestinal barrier (Kuo et al., 2019; Tian et al., 2021). Therefore, the intestinal permeability in the TNBS-induced colitis model is determined by measuring the serum fluorescence intensity of FITC-dextran. We found that the serum fluorescence intensity of FITC-dextran in TNBS-induced mice was significantly higher than in normal mice, and SM934 markedly reduced the intestinal permeability of CD mice (Figure 2A). On the other hand, with quantitative morphometry being confirmed, the colonic E-cadherin significantly reduced in vehicle mice by contrast with normal mice. In contrast, SM934 notably promoted the protein expression, which is closely related to the intestinal permeability in CD (Figure 2B). Correspondingly, Western blot analysis showed that the TJ proteins of E-cadherin, β-catenin, and occludin were enhanced under SM934 treatment compared with TNBS-induced colitis mice in colon tissues (Figures 2C,D). In addition, the numbers of hyper-proliferation of intestinal epithelial cells that reside in crypts were visualized by the Ki67 antigen. As shown in Figure 2E, administration of SM934 significantly prevented the Ki67 protein expression in colon tissue crypts, following TNBS-induced mice with colitis. These data demonstrated that SM934 protected intestinal barrier function by decreasing the intestinal permeability, promoting TJ protein expression, and suppressing the abnormal epithelial proliferation against TNBS-induced colitis.

### SM934 inhibited apoptosis in trinitrobenzene sulfonic acid-induced colitis mice

**FIGURE 3:**
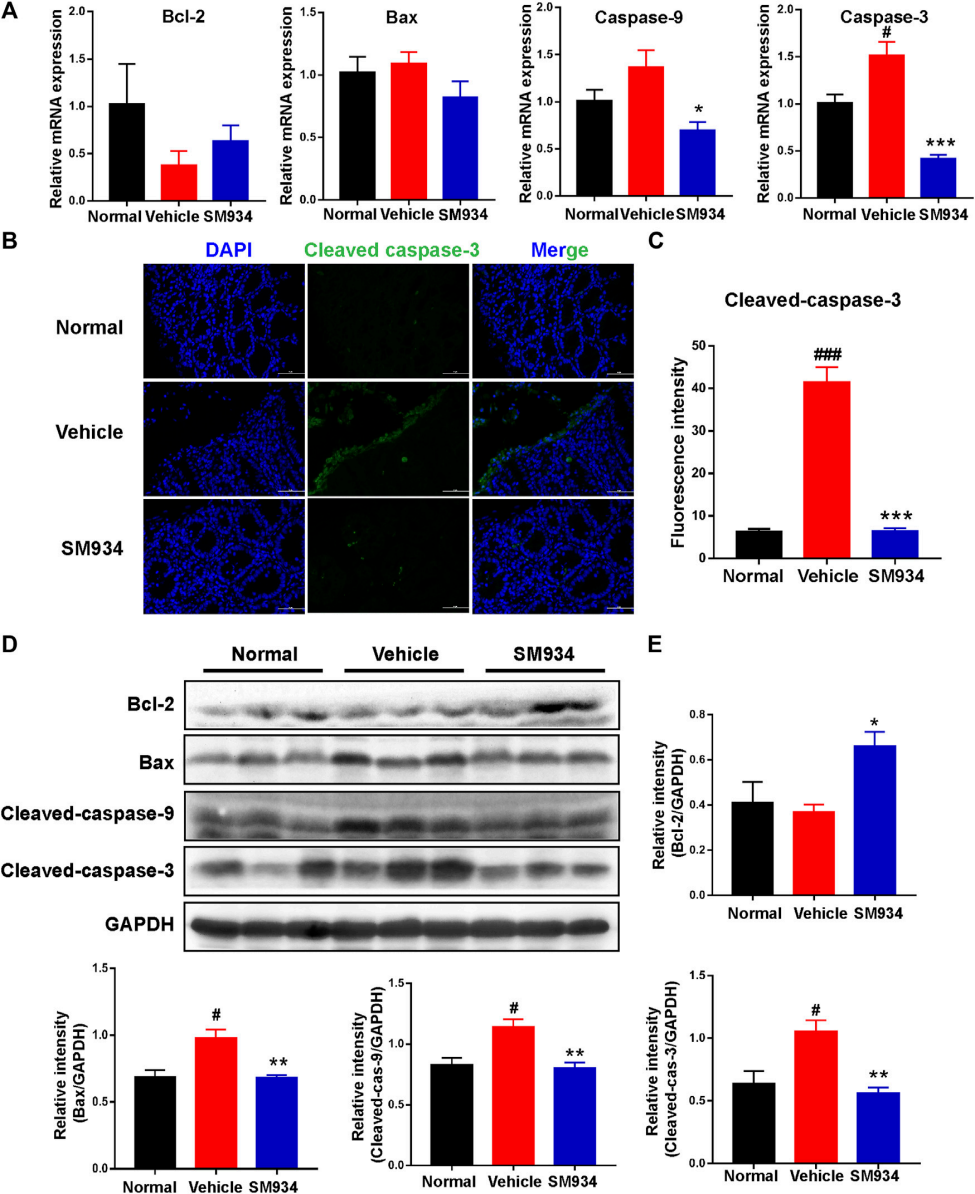
SM934 inhibited apoptosis in TNBS-induced colitis in mice. **(A)** QPCR analyzed the apoptosis-related gene levels of colon tissues in mice. **(B)** Representative fluorescence images of cleaved caspase-3 in colon sections (scale bar, 50 µm). **(C)** Quantification of cleaved caspase-3-positive cells in colon tissues. **(D)** Western blotting assayed the expressions of apoptosis-related proteins of bcl-2, bax, cleaved caspase-9, and cleaved caspase-3 in colon tissue. **(E)** Relative intensity of proteins to GAPDH. Data are shown as means ± SEM (*n* = 3–4). ^#^ *p* < 0.05 and ^###^ *p* < 0.001 vs*.* normal group; ^*^ *p* < 0.05, ^**^ *p* < 0.01, and ^***^ *p* < 0.001 vs*.* vehicle group.

Several lines of evidence illustrate that apoptosis regulates the replacement rate of epithelial cells. However, excessive IEC apoptosis is considerably high in the colons of IBD patients or animal colitis models that can disrupt the mucosal epithelial barrier and cause severe gut pathology (Di Sabatino et al., 2003; Edelblum et al., 2006). Thus, to clarify the effect of SM934 on apoptosis in TNBS-induced colitis mice is necessary. Here, qPCR analyzed the levels of apoptosis-related genes, including anti-apoptotic Bcl-2, pro-apoptotic Bax, and apoptosis initiator caspase-9 and executor caspase-3 in colon tissues. As shown in Figure 3A, the mRNA level of Bcl-2 was slightly increased, and the anti-apoptosis genes Bax, caspase-9, and caspase-3 largely decreased in SM934-treated colitis mice. In addition, cleaved caspase-3, a mark of apoptosis, was elevated with immunofluorescence staining in colon tissues. Interestingly, the result found that cleaved caspase-3 was markedly upregulated on superficial epithelial cells lining the lumen of colon tissues in TNBS-treated mice. As a result, the fluorescence intensity of cleaved caspase-3 protein was nicely reversed by the SM934 treatment in the colon tissues of colitis mice (Figures 3B,C). In line with this, the protein expressions of Bcl-2, Bax, cleaved caspase-9, and cleaved caspase-3 in colon tissues were determined with a Western blot assay. Notably, all of the protein levels were reversed with SM934 treatment in colitis mice (Figures 3D,E). These data implicated that SM934 enhanced epithelial barrier function in colitis at least in part by inhibiting epithelial cell apoptosis.

### SM934 protected the epithelial barrier function of intestinal epithelial cells by regulating the aberrant expression of tight junction proteins and apoptosis

To further explore whether SM934 protected the intestinal barrier and inhibited apoptosis directly acting on colonic epithelial cells, the study was conducted using human colonic epithelial cell lines Caco-2 and HT-29 cells exposed to TNF-α, which is commonly known to initiate epithelial barrier damage and apoptosis in IECs, which mimic this progression *in vitro* (Bruewer et al., 2003; Cao et al., 2020; Woznicki et al., 2020). Therefore, we constituted the Caco-2 cell monolayer model to evaluate the effect of SM934 on barrier function by measuring the TEER values and intestinal permeability.

**FIGURE 4:**
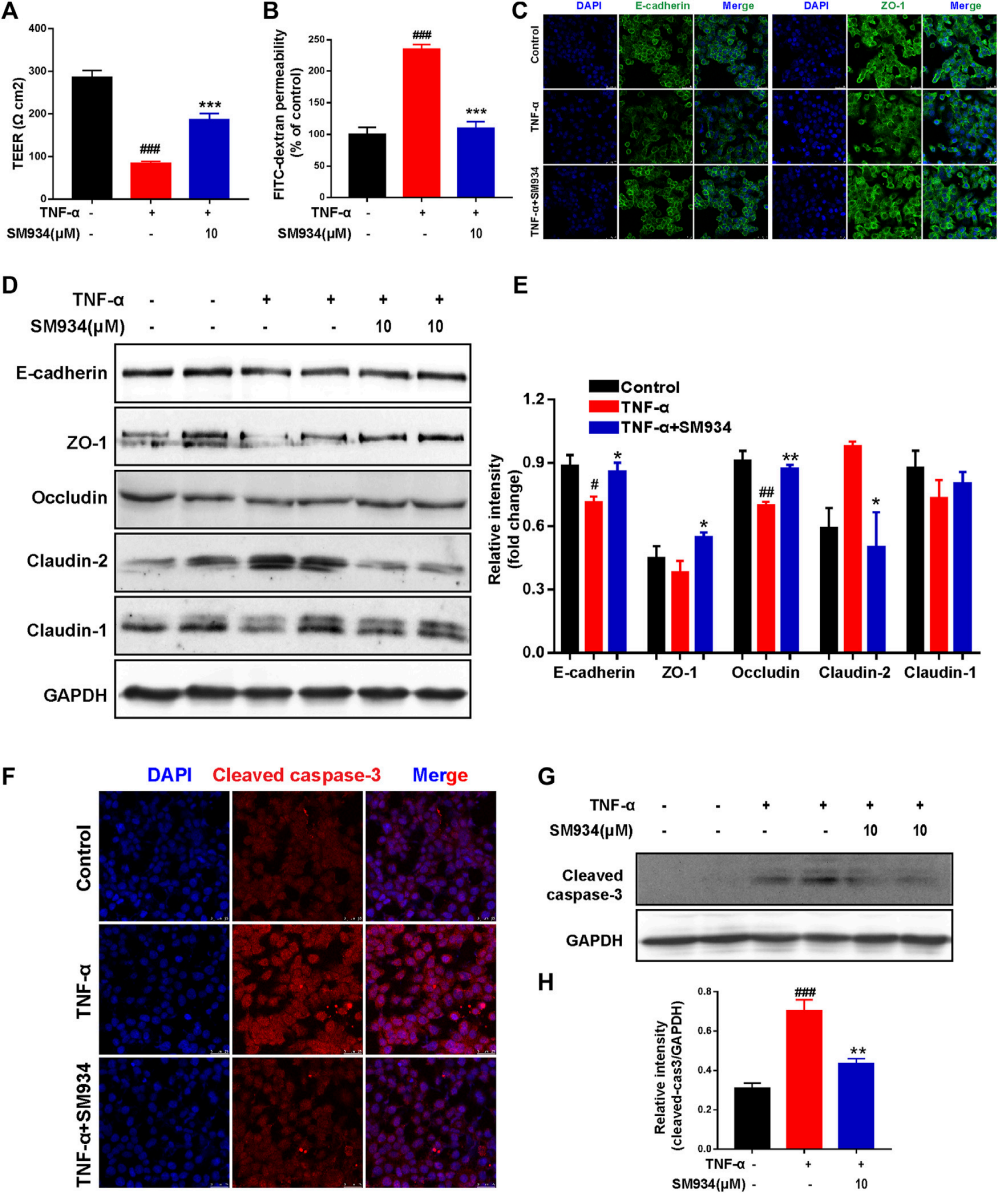
SM934 protected the epithelial barrier function of IECs by regulating the aberrant expression of TJ proteins and apoptosis. Caco-2 cells were treated with or without TNF-α (100 ng/ml) in the absence or presence of SM934 (10 μM) for 24 h **(A)** Transepithelial electrical resistance (TEER) and **(B)** permeability of FITC-dextran of Caco-2 cell monolayers were detected. HT-29 cells were pre-incubated with SM934 (10 μM) for 1 h and then exposed to hTNF-α (100 ng/ml) in the presence or absence of SM934 for 24 and 72 h and then using for immunofluorescence and Western blotting assays, respectively. **(A)** Representative fluorescence images of E-cadherin and ZO-1 stimulated by TNF-α for 24 h in HT-29 cells and measured with a confocal laser-scanning microscope (scale bars, 25 µm). **(B)** Western blotting assayed the TJ protein expressions of E-cadherin, ZO-1, occludin, claudin-2, and claudin-1 and were treated with TNF-α for 72 h in HT-29 cells. **(C)** Relative intensity of proteins to GAPDH. **(D,E)** HT-29 cells were treated with TNF-α in the presence or absence of SM934 in HT-29 cells for 24 h to measure the expression of cleaved caspase-3 by immunofluorescence staining (Scale bars, 25 µm) and Western blot assay. **(E)** Relative intensity of protein to GAPDH. Data are shown as means ± SEM from three representative experiments. **(F)** The expression of cleaved -caspase 3 in HT-29 cells was detected by Immunofluorescence staining (Scare bars, 25 μm). **(G)** The protein level of cleaved caspase-3 in HT-29 cells was detected by western blot. **(H)** The relative intensity of cleaved-caspase-3 to GAPDH. ^#^ *p* < 0.05 and ^##^ *p* < 0.01 vs*.* control group; *p < 0.05 vs*.* TNF-α group.

As shown in Figures 4A,B, SM934 obviously decreased the TEER value and blocked the flux of FITC-dextran in TNF-α-induced Caco2 cell monolayer model, while exploring the protective effect of SM934 on the TJ proteins injured by TNF-α in HT-29 cells. Immunofluorescence analysis demonstrated that administration with SM934 markedly increased the fluorescence intensity of E-cadherin and ZO-1 in TNF-α-induced HT-29 cells (Figure 4C). Similarly, under the injury of TJ proteins by TNF-α in HT-29 cells, Western blotting analysis demonstrated that SM934 significantly upregulated the protein levels of E-cadherin, ZO-1, and occludin and reduced the expression of claudin-2, whereas the increase of claudin-1 was not obvious (Figures 4D,E). Furthermore, it is well known that excessive epithelial cell death can contribute to the disruption of the gut barrier (Buonpane et al., 2020). Therefore, we next assessed whether the observed barrier-protective effects of the SM934 could be mediated by attenuating epithelial cell apoptosis. Consistently, the results revealed that HT-29 cell exposure to TNF-α remarkably cleaved caspase-3 formation. Undoubtedly, treatment with SM934 significantly inhibited caspase-3 cleaved in TNF-α-treated HT-29 cells (Figures 4F–H). On the basis of these findings, we could confirm the protective effects of the SM934 treatment of colitis on the intestinal barrier based on the decrease in intestinal permeability, regulating the expression of TJ proteins and against IEC apoptosis *in vivo* and *in vitro*.

### SM934 blocked the caspase-1-mediated pyroptosis-associated activation of NOD-like receptor family pyrin domain-containing 3 inflammasome/gasdermin in trinitrobenzene sulfonic acid-induced colitis

**FIGURE 5:**
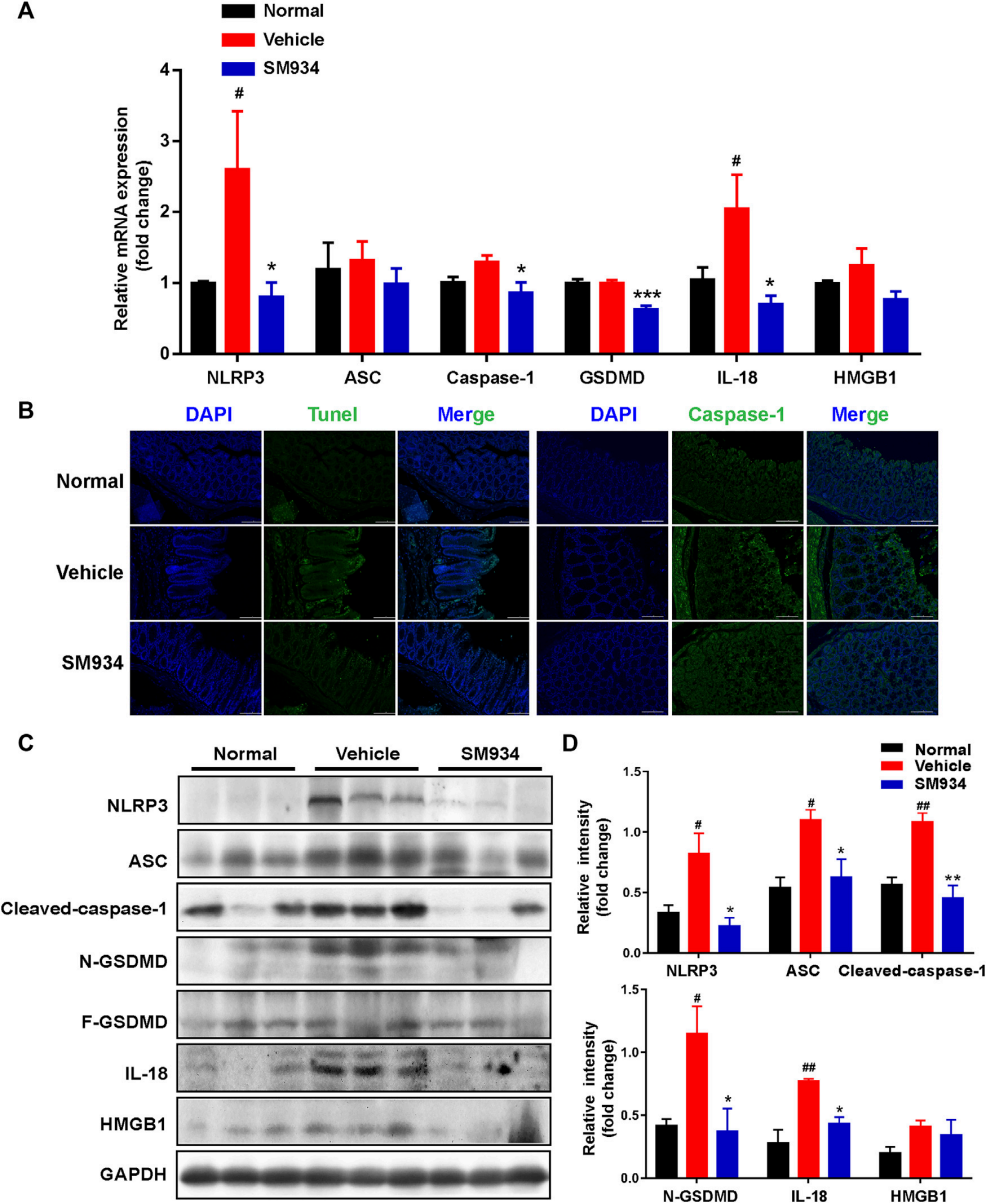
SM934 inhibited caspase-1-mediated pyroptosis activation in TNBS-induced colitis. **(A)** Levels of pyroptosis-related genes as determined by qPCR in colon tissues. **(B)** Pyroptosis hallmarkers of DNA fragmentation and caspase-1 expression were determined using the TUNEL staining assay and immunofluorescence staining in colon tissues, respectively (scare bar, 100 µm). **(C)** Expression of pyroptosis-related proteins was analyzed by Western blot assay. **(D)** Quantitative analysis of pyroptosis-related protein expression was conducted. Data are shown as means ± SEM (*n* = 3–4). ^#^ *p* < 0.05 and ^##^ *p* < 0.01 vs*.* normal group; *p < 0.05, **p < 0.01, and ***p < 0.001 vs*.* vehicle group.

It is well known that caspase-1 mediates pyroptosis when activated by the canonical inflammasome NLRP3, interacts with the adaptor protein ASC in response to microbial infection after the intestinal epithelial barrier disruption, and then cleaves the pyroptosis effector GSDMD, releasing pro-inflammatory mediators mature IL-18 and HMGB1 to the outside of cells, which is associated with the pathogenesis of Crohn’s disease (Villani et al., 2009; Zaki et al., 2010). We investigated whether SM934 exerted a protective effect against TNBS-induced colitis *via* the blockade of the canonical inflammasome caspase-1-mediated pyroptosis activation. First, we detected the mRNA expressions of pyroptosis-associated factors in colon tissue. As shown in Figure 5A, the mRNA expressions of NLRP3, ASC, caspase-1, GSDMD, IL-18, and HMGB1 were shown to increase in different extents in colitis tissues than in control tissues, whereas SM934 treatment decreased the expression of these genes. In line with this, we further found that the pyroptotic cells exhibited DNA fragmentation examined by TUNEL-positive sites and the pyroptosis hallmark of caspase-1. Importantly, the pyroptotic cells were remarkably weakened, as evidenced by decreasing the percentage of TUNEL-positive cells and abolishing the fluorescence intensity of caspase-1 in colon tissues under the administration of SM934 in colitis mice (Figure 5B). On the other hand, the protein levels of these factors were also obviously downregulated after treatment with SM934 in the colon tissues of colitis mice (Figures 5C,D). These data indicated that SM934 could improve TNBS-induced colitis in mice, possibly *via* affecting the cells’ pyroptosis, especially IECs.

### SM934 reduced epithelial cell pyroptosis by preventing the activation of NOD-like receptor family pyrin domain-containing 3 inflammasome/caspase-1/gasdermin signals

**FIGURE 6:**
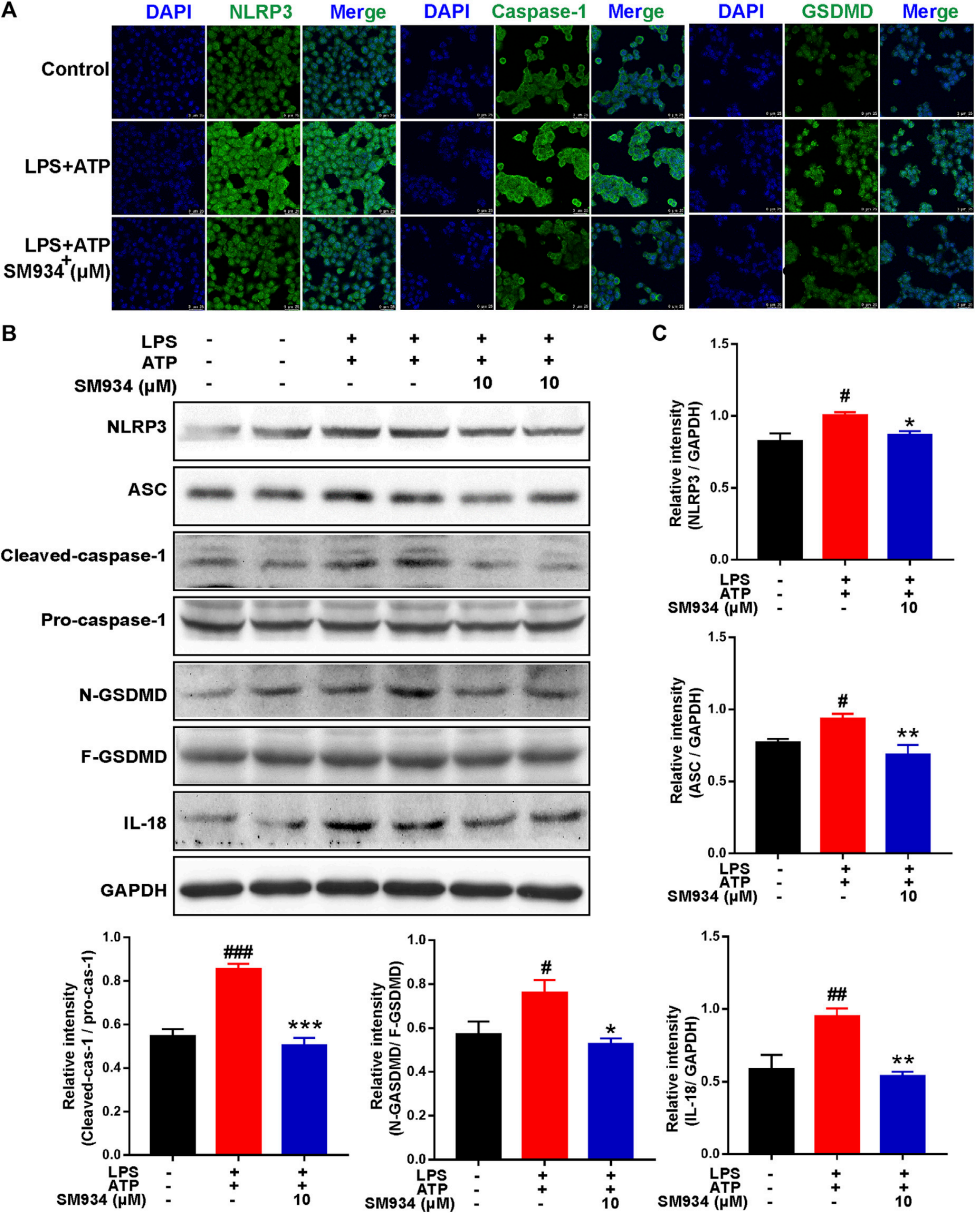
SM934 reduced the epithelial pyroptosis through preventing the activation of NLRP3/caspase-1/GSDMD signals *in vitro*. HT-29 cells were treated with LPS (1 μg/ml) for 12 h, and then treated with ATP (5 mM A) for 2 h to induce pyroptosis without or with SM934 (cells pro-cultured with SM934 for 1 h). **(A)** Immunofluorescence staining was performed to detect the formation of NLRP3, caspase-1, and GSDMD in HT-29 cells (scale bar, 25 µm). **(B)** Protein levels of NLRP3, ASC, caspase-1 (p45, p20), GSDMD-F and GSDMD-N, and IL-18 examined by immunoblotting in HT-29 cells. **(C)** Relative intensities of proteins were also conducted. Data are shown as means ± SEM from three representative experiments. ^#^ *p* < 0.05, ^##^ *p* < 0.01, and ^###^ *p* < 0.001 vs*.* control group; *p < 0.05, **p < 0.01, and ***p < 0.001 vs*.* LPS and ATP group.

As shown in *in vivo* data, the massive pyroptosis of IECs in colonic tissue of CD mice was observed. Next, we conducted *in vitro* experiments to further investigate the effects of SM934 on pyroptosis in IECs. We established a cellular pyroptosis model in HT-29 cells stimulated with LPS (1 μg/ml) for 12 h and then with ATP (5 mM) for 2 h in the presence or absence of SM934 treatment. IF staining revealed that the fluorescence intensity of NLRP3, caspase-1, and GSDMD visibly increased after LPS and ATP co-stimulated. However, the upregulation of those pyroptosis factors could be reversed by SM934 (Figure 6A). As a further confirmation, the protein levels of NLRP3, ASC, caspase-1 (p45, p20), and GSDMD-F and GSDMD-N, and IL-18 were also detected after co-stimulation of ATP + LPS. Meanwhile, SM934 treatment significantly decreased the key factors of pyroptosis protein levels of the NLRP3 inflammasome, ASC, and the cleaved caspase-1 and GSDMD-N and IL-18 releasing *in vitro* (Figures 6B,C). Together, the data further verified that SM934 inhibited IECs’ pyroptosis against colitis.

### SM934 suppressed the activation of mitogen-activated protein kinase and nuclear factor-kB signaling pathways both *in vivo* and *in vitro*

**FIGURE 7:**
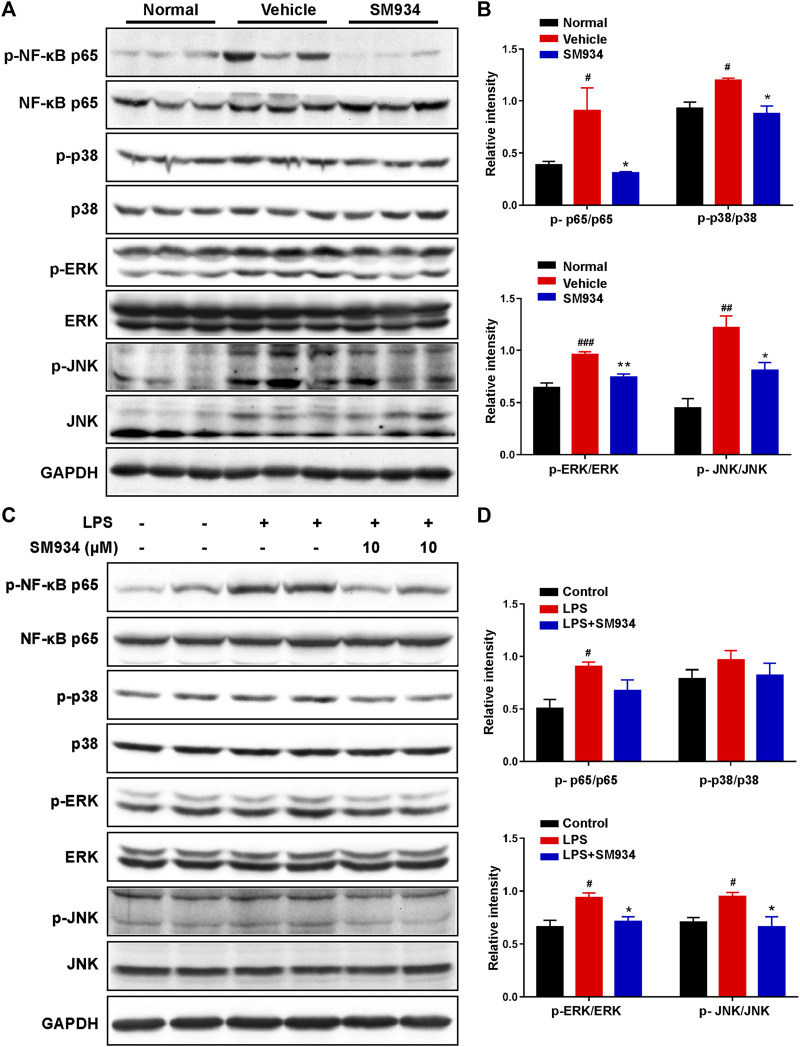
SM934 suppressed the activation of NF-κB and MAPK signaling pathways both *in vivo* and *in vitro*. **(A)** Phosphorylation of NF-κB, p38, ERK, and JNK in colon tissues was detected with a Western blot. **(B)** Relative intensities of the phosphorylation levels of NF-κB, p38, ERK, and JNK as normalized against total proteins. **(C)** HT-29 cells were stimulated with LPS (1 μg/ml) for 24 h without or with SM934 (cells pro-cultured with SM934 for 1 h); then, cells were harvested for NF-κB, p38, ERK, and JNK signals tested with Western blot. **(D)** Relative intensities of the phosphorylation levels of NF-κB, p38, ERK, and JNK as normalized against total proteins *in vitro*. Data are shown as means ± SEM. ^#^ *p* < 0.05, ^##^ *p* < 0.01, and ^###^ *p* < 0.001 vs*.* control group; *p < 0.05 and **p < 0.01 vs*.* LPS group.

MAPK and NF-κB are key upstream signals in NLRP3-caspase-1 pyroptosis activation and have been considered as pro-inflammatory signals in IBD (Cao et al., 2021). Above all, we explored the effects of SM934 on the MAPK and NF-κB pathways in TNBS-induced colitis in mice. As shown in Figures 7A,B, SM934 significantly inhibited the phosphorylation levels of NF-κB, ERK, p38, and JNK in the colon tissues of colitis mice. Subsequently, *in vitro*, HT-29 cells were stimulated with LPS (1 μg/ml) for 12 h without or with SM934, then cells were harvested for NF-κB, and p38, ERK, and JNK signals were tested with Western blot. Consistent with the observations *in vivo*, we found that the levels of p-NF-κB, p-p38, p-ERK, and p-JNK also decreased after treatment with SM934 and exposure to LPS in HT-29 cells (Figures 7C,D). These results illustrated that SM934 protected against colitis through the blockade of NF-κB and MAPK signals by inhibiting caspase-1-mediated inflammatory pyroptosis activation in IECs.

## Discussion

Crohn’s disease (CD) is a nonspecific, chronic, and relapsing multi-factorial inflammatory disease of the gut without radical treatment drugs so far. Although anti-tumor necrosis factor-α (TNF-α) is widely used as the effective therapeutic medicine for CD, over time, there are still a large number of patients who fail to respond or lose response to it (Cohn et al., 2017; Park et al., 2019). Hence, there is an urgent need to search for effective strategies for treatment of CD. In these studies, we found that SM934 had a significant improvement in body weight loss and colon length shortening in TNBS-induced colitis. Further experiments based on the colon images and histopathological analysis indicated that SM934 treatment remarkably reduced the phenotypic changes, including multifocal inflammatory lesions with epithelial hyperplasia, ulceration, dead epithelial cells, and crypt abscess (Figure 1), which are the major pathophysiological characteristics of colitis (Mizushima et al., 2013).

Recent studies have intensively suggested that the defective intestinal epithelial barrier function and excessive IEC death had a strong link with colitis (Mizushima et al., 2013; Blander, 2018). Also, the intestinal epithelium is composed of a monolayer of columnar epithelial cells and forms a physical and biochemical barrier against intestinal pathogens to maintain intestinal tissue homeostasis. Specifically, the adjacent IECs were connected by AJ (e.g., E-cadherin and β-catenin) and TJ (e.g., occludin ZO-1, claudin-1, and claudin-2) proteins to evaluate the degree of intestinal epithelial barrier injury, whose defects can increase intestinal epithelial permeability and cause gut diseases (Peterson and Artis, 2014; Zhuang et al., 2019). In particular, E-cadherin is a calcium-dependent cell adhesion receptor highly expressed by epithelial cells and is essential for the regulation of cell proliferation and apoptosis in colitis (Di Sabatino et al., 2003; Tian et al., 2021). Therefore, regulation of IEC-expressed AJ and TJ proteins facilitates intestinal homeostasis. Consistently, here in our study, we discovered that SM934 reduced the intestinal epithelial permeability and restored the aberrant expressions of E-cadherin, β-catenin, and occludin; at the same time, the abnormal epithelial cell proliferation was also inhibited in colitis mice (Figure 2). Of note, *in vitro*, we also demonstrated that SM934 decreased the intestinal epithelial permeability and restored the aberrant expressions of E-cadherin, ZO-1, occludin, claudin-2, and claudin-1 in IEC exposure to TNF-α (Figures 4A–E). In addition, TNF-α usually has been proved to participate in the destruction of the intestinal epithelial barrier and induces IEC apoptosis (Li et al., 2021). On the other hand, the reports suggest that apoptosis is thought to be one of the major accelerators of the disruption of intestinal epithelial integrity in colitis, which is mainly executed by the apoptosis effector caspase-3 and combined with the intrinsic apoptosis pathway associated with caspase-9, the pro-apoptotic protein Bax and the anti-apoptotic protein Bcl-2 to mediate the apoptotic signals (Dirisina et al., 2011; Lin et al., 2017; Kuo et al., 2019). In spite of this, SM934 regulated the balanced Bcl-2 and BAX, and cleaved caspase-9 and cleaved caspase-3 in colitis (Figure 3), and also downregulated the cleaved caspase-3 in TNF-α-induced IEC apoptosis *in vitro* (Figures 4F–H). Thus, the results manifested SM934-ameliorated colitis might be regulating the balance between the intestinal epithelial barrier based on intestinal permeability, the At/TJ proteins, and apoptosis in IECs.

Apart from apoptosis, another form of major lytic cell death in response to acute external damage ultimately elicits excessive inflammatory response and immune dysregulation to disturb the intestinal epithelial barrier termed inflammatory pyroptosis, which is involved in the pathological process of IBD (Cao et al., 2021). To date, caspase-1 is the most widely studied cysteine protease that regulates the pyroptosis pathway *via* inflammasome NLRP3 activation, which has been associated with susceptibility to Crohn’s disease (Song-Zhao et al., 2014). Recently, Liu et al. (2017) reported that the NLRP3 inflammasome in colonic epithelial cells (CECs) isolated from IL-10^−/−^ mice and Crohn’s patients was spontaneously active and hyperactive when stimulated with LPS. In addition, NLRP3 assembly and activation in response to pathogen-associated molecular patterns (PAMPs) and damage-associated molecular patterns (DAMPs), such as LPS, extracellular ATP, particulate matter, and certain bacterial toxins, induce the cleavage of pro-caspase-1 (Aguilera et al., 2014). Subsequently, the activation and cleaved caspase-1 cleaves the pyroptosis effector of GSDMD that formed pores in the cell membrane. Hence, the mature IL-18 and the high mobility group box1 (HMGB1) are sequentially released outside of the cells (Chen et al., 2020) and both of them are evidenced to have high expression in the intestinal epithelium in IBD (Pizarro et al., 1999; Fujii et al., 2008). Furthermore, IL-18 is a critical pro-inflammatory cytokine secreted by IECs of colons from Crohn’s disease patients, involved in an inflammatory caspase-1-dependent manner (Dupaul-Chicoine et al., 2010). Intriguingly, one of the remarkable findings of the present study was that SM934 had a robust effect against pyroptosis not only repressing the pyroptosis-associated gene transcription levels but also preventing the apoptosis-associated proteins of NLRP3, ASC, cleaved-caspase-1, GSDMD-N, IL-18, and HMGB1 in the colon tissues of colitis mice (Figure 5). Therefore, we, furthermore, adopted LPS- and ATP-induced cell pyroptosis in HT-29 cells, which were usually used to activate the NLRP3-caspase-1 inflammasome-mediated pyroptosis signals in IECs and macrophages (Bulek et al., 2020; Deng et al., 2020). Consistent with previous observations, the pyroptosis signals are also reversed by SM934 *in vitro* (Figure 6). The resulted indicated that SM934 restrained the IEC pyroptosis against intestinal injury of colitis *in vivo* and *in vitro*.

It is well known that NF-κB is a prime transcriptional regulator of caspase-1-mediated pyroptosis. NF-κB activation in IECs can result in the increase of inflammatory cytokines, which are important in the pathogenesis of IBD (Egan et al., 2004; Mikuda et al., 2020; Rizzo et al., 2021). On the other hand, hyper-phosphorylation of MAPK, consisting p38, ERK, and JNK molecules, eventually activates NF-κB and has been revealed to be another signal cascade initiated in colitis (Zhang Y. et al., 2021; Cao et al., 2021). We hypothesized SM934 blocked the signaling of NF-κB and MAPKs in colitis. Of interest, we noticed that SM934 reduced the phosphorylation levels of NF-κB, p38, ERK, and JNK of colon tissues not only in TNBS-induced colitis but also in LPS-induced HT-29 cells (Figure 7). Based on these results, we confirmed that SM934 protected against colitis by decreasing IEC pyroptosis might be *via* the NF-κB/MAPK pathway.

In conclusion, our study demonstrated that oral administration of SM934 exerted therapeutic effects in experimental colitis induced by TNBS *via* inhibiting the IECs’ apoptosis and blocking the caspase-1-mediated pyroptosis-associated NLRP3/NF-κB/MAPK signal pathways. Additionally, SM934 protected epithelial cells from apoptosis under inflammatory condition while hardly affecting their survival in physiological status. Furthermore, the relationship between the intestinal epithelial barrier function and pyroptosis-related inflammation needs to be further explored in IBD. We hope that the present study can provide a clue for the mechanistic investigation of the protective effects of SM934 on the intestinal barrier against colitis.

## Data Availability

The original contributions presented in the study are included in the article/Supplementary Materials; further inquiries can be directed to the corresponding authors.
