# Supplementary material for: Artemisinin analog SM934 alleviates epithelial barrier dysfunction via inhibiting apoptosis and caspase-1-mediated pyroptosis in experimental colitis
Source: Front Pharmacol. 2022 Sep 1;13:849014. doi: 10.3389/fphar.2022.849014 (PMC9477143; doi:10.3389/fphar.2022.849014)
Supplement: Supplementary file 1 [file DataSheet2.docx]

**­Figure 2. Original immunohistochemistry images**

**E-cadherin protein (Three sample images of each group)**

Normal Vehicle SM934 10mg/kg

**
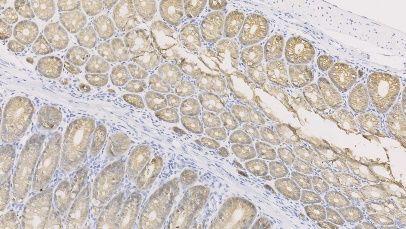

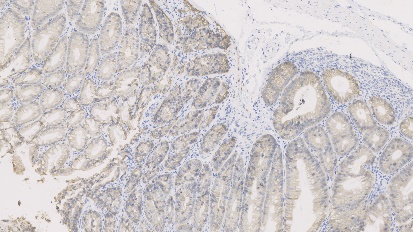

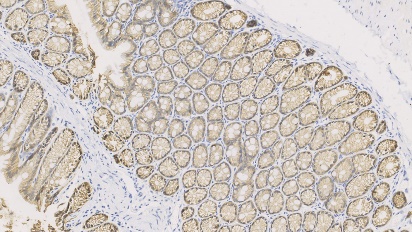
**

**
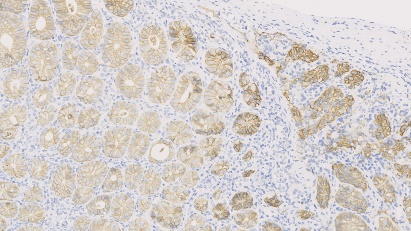

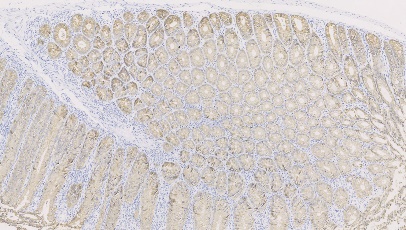

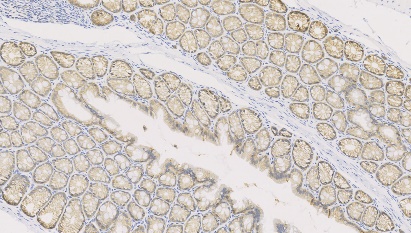
**

**
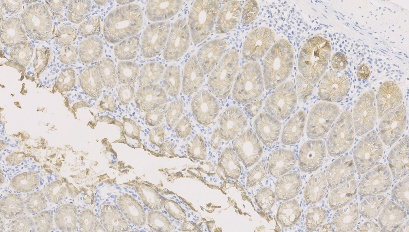

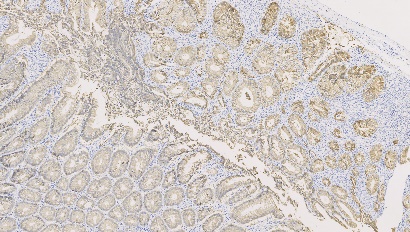

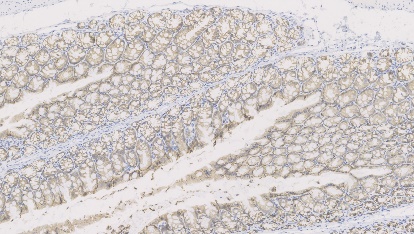
**

**Ki67 protein (Three sample images of each group)**


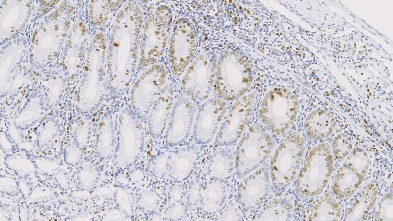

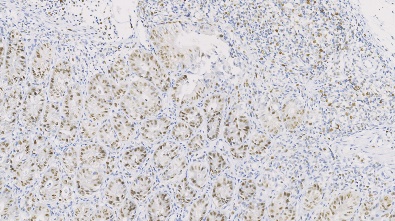

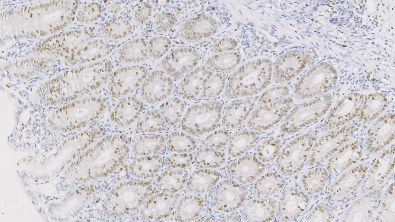

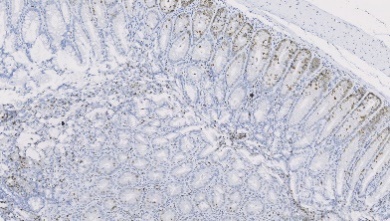

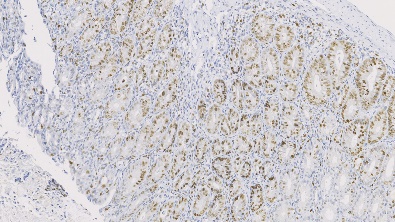

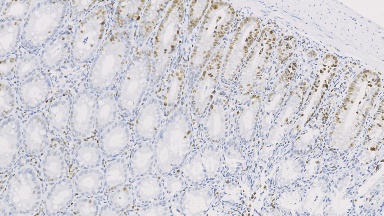

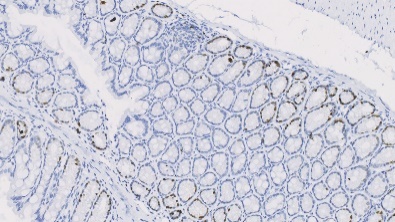
 Normal Vehicle SM934 10mg/kg


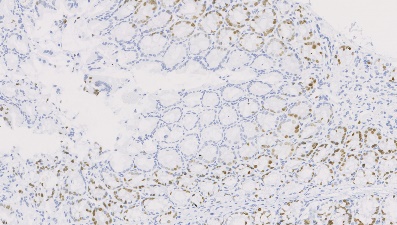
­­­­


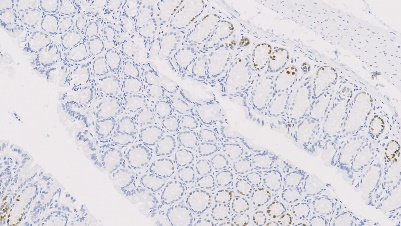


**Figure 3. Original** [**immunofluorescence**](javascript:;)**images**

**Cleaved caspase-3 (Three sample images of each group)**

**Normal**

**
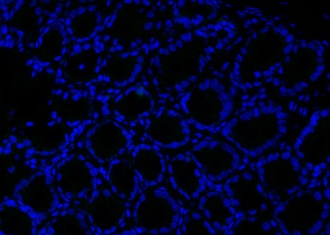

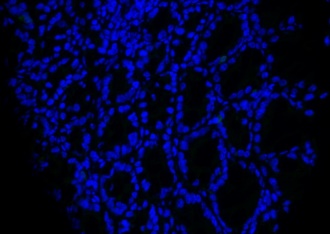

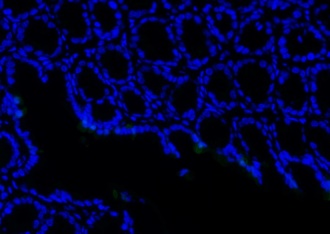

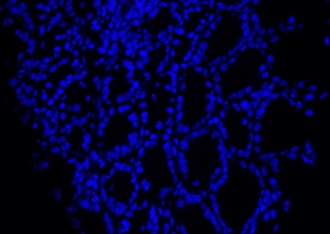

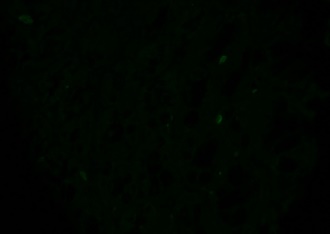

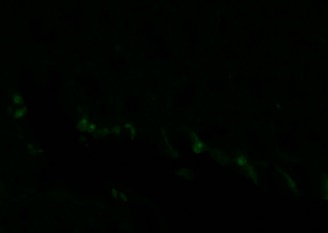

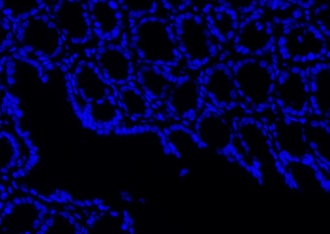
**

**DAPI**

**
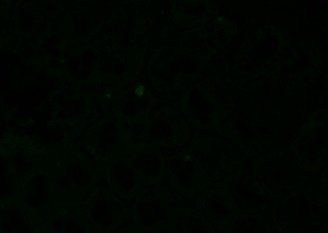
**

**Cleaved**

**caspase-3**

**
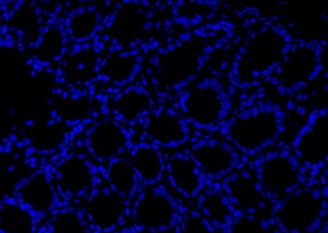
**

**Merge**

**Vehicle**

**
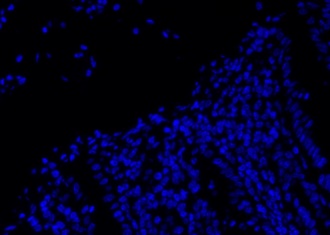

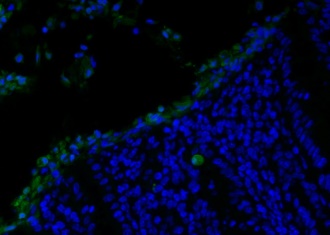

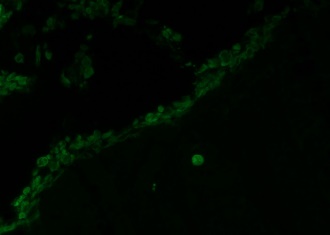

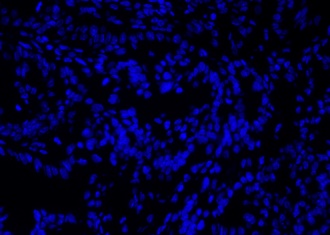

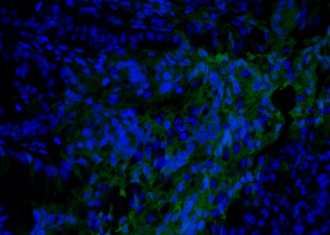

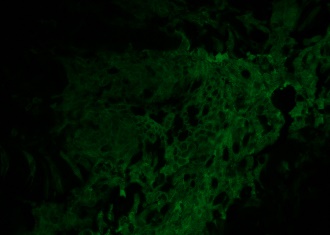

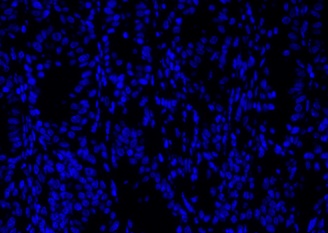

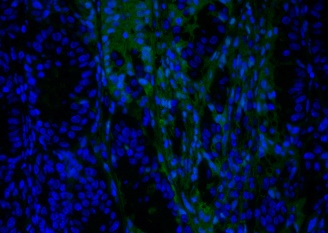

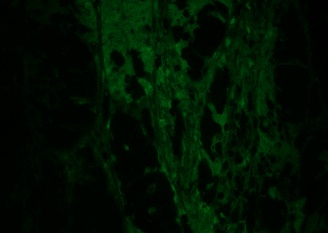
**

**DAPI**

**Cleaved**

**caspase-3**

**Merge**

**SM934 10mg/kg**


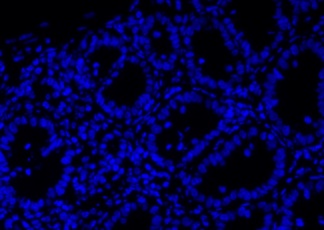

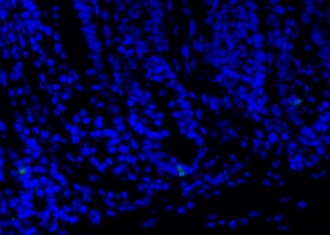

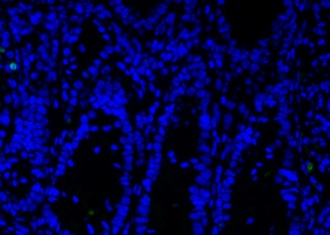

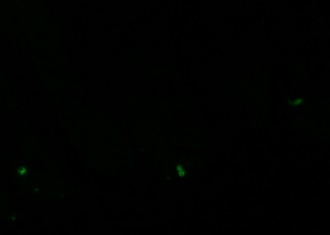

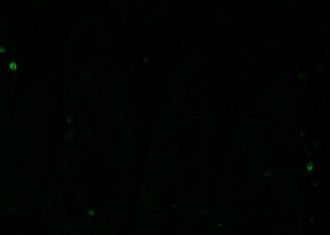

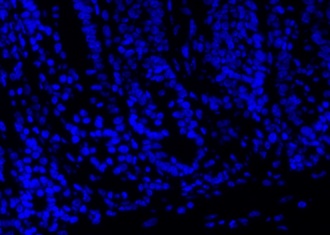

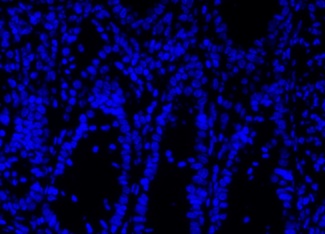


**DAPI**


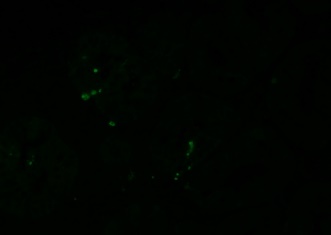


**Cleaved**

**caspase-3**


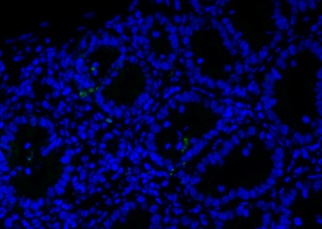


**Merge**

**Figure 4. Original Immunofluorescence cytochemistry images**

**Control-1** **Control-2 Control-3**


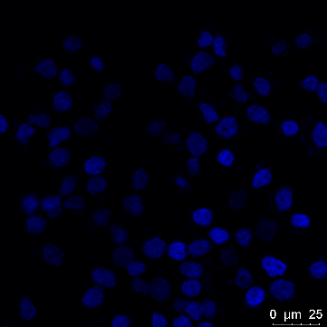

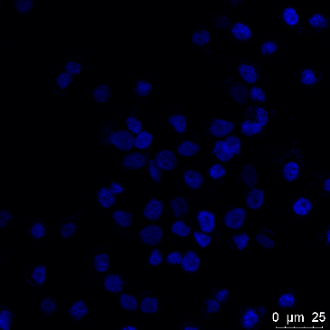

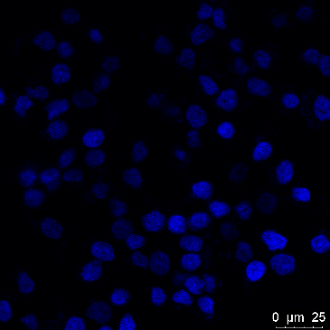


**DAPI**


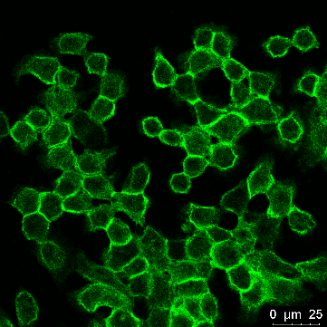

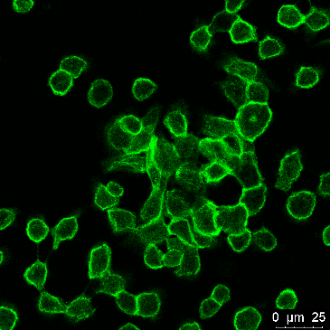

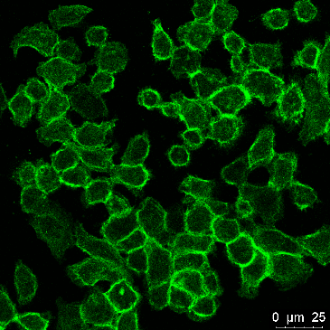


**E-cadherin**


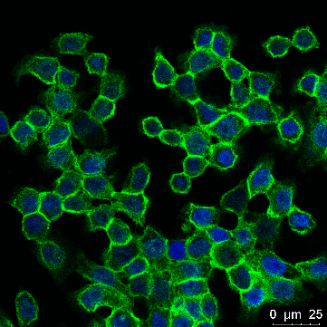

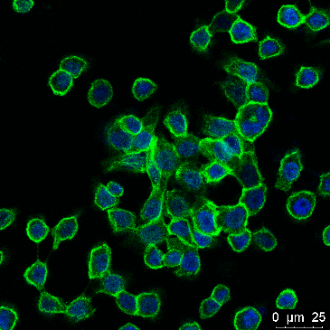

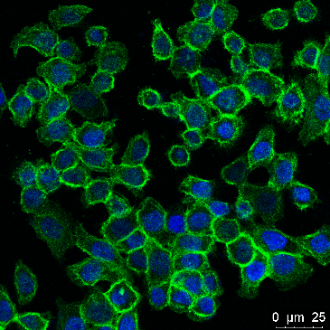


**TNF-α-1** **TNF-α-2**  **TNF-α-3**

**TNF-α-1** **TNF-α-2 TNF-α-3**


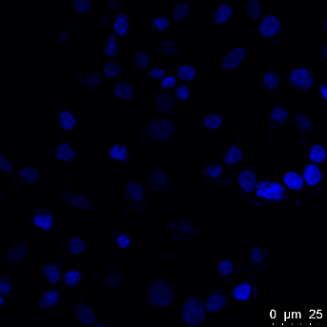

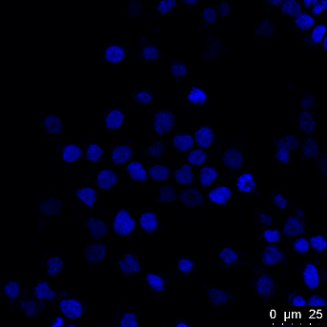

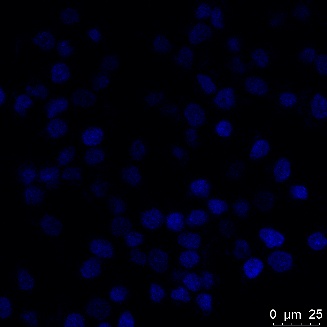


**DAPI**


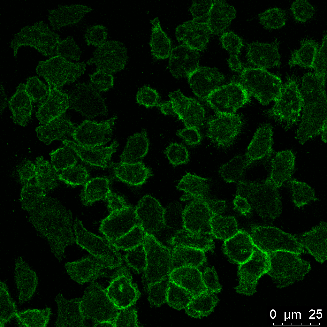

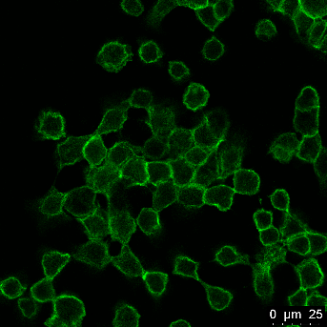

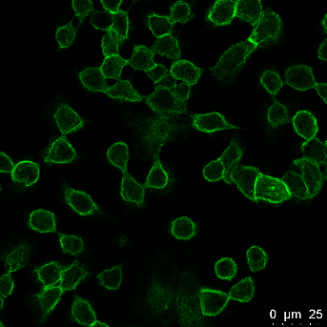


**E-cadherin**


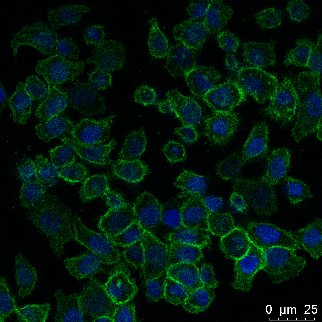

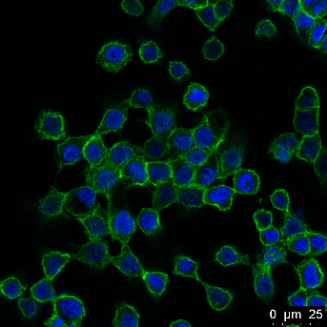

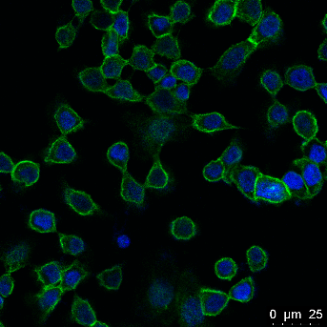


**Merge**

**SM934-1 SM934-2 SM934-3**


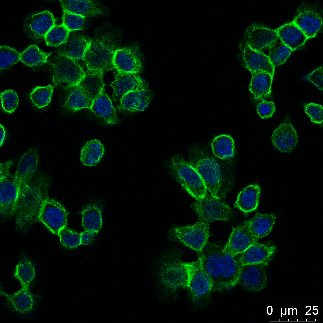

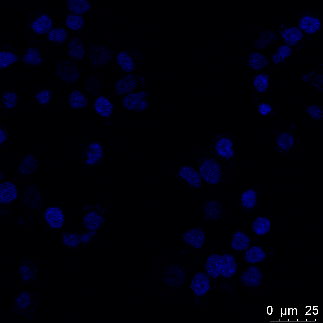

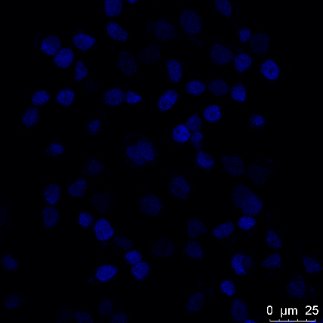

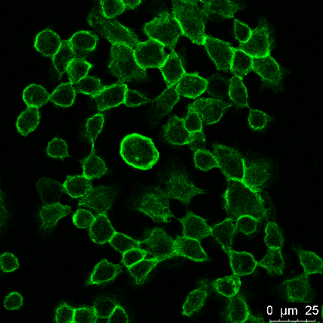

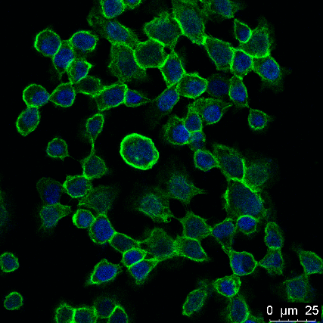

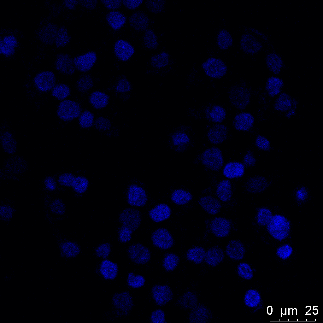

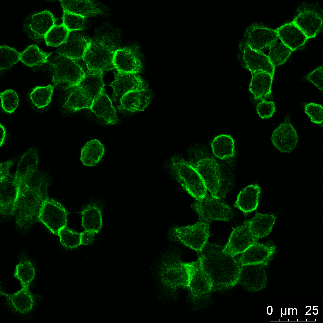

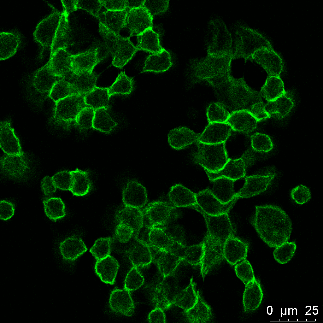

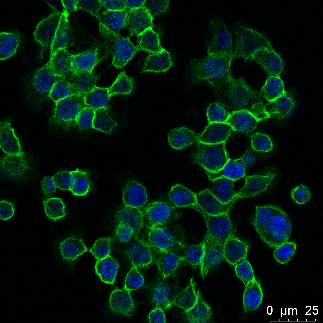


**DAPI**

**E-cadherin**

**Merge**

**Control-1** **Control-2 Control-3**

**Control-1** **Control-2 Control-3**


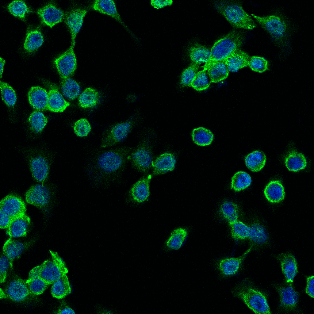

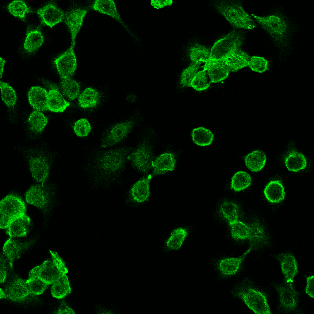

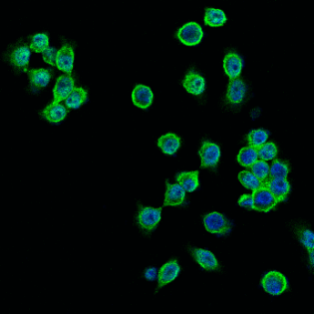

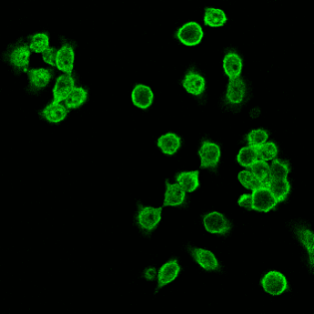

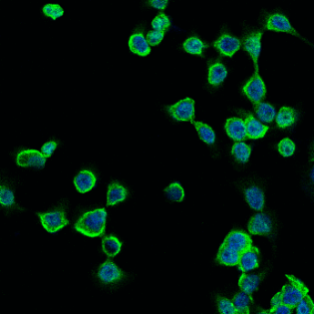

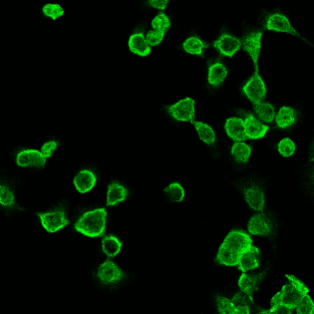


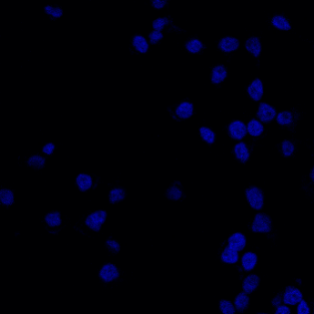

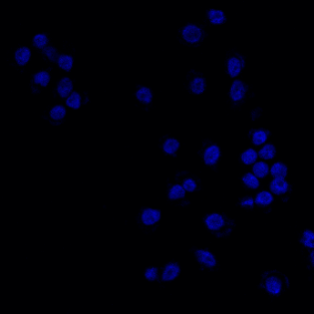

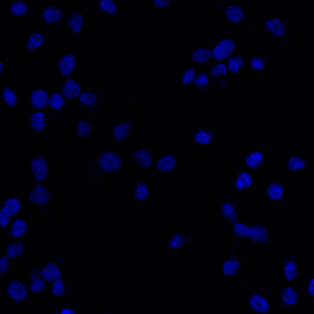


**DAPI**

**ZO-1**

**Merge**

**TNF-α-1** **TNF-α-2 TNF-α-3**


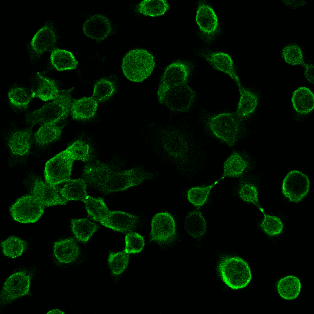

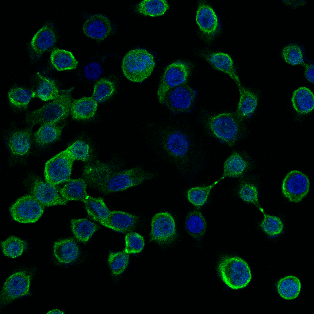

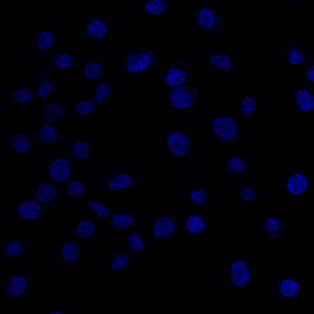

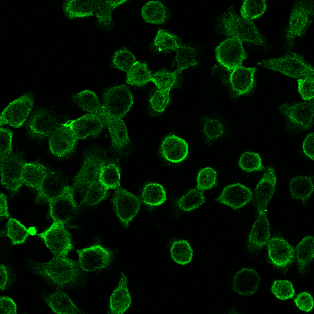

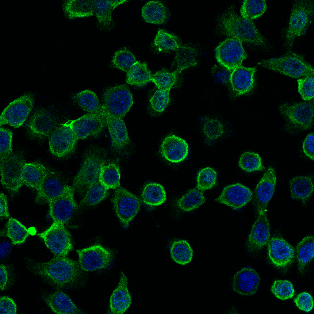

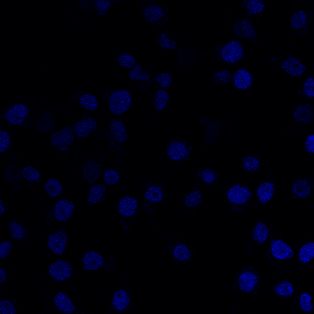

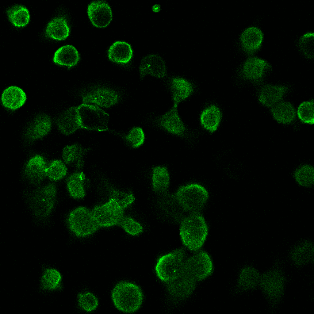

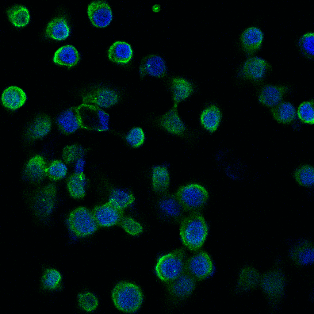

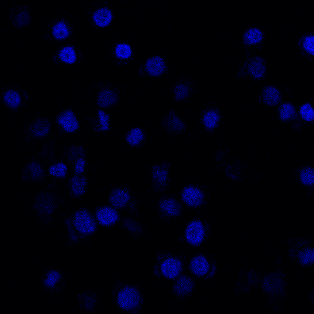


**DAPI**

**ZO-1**

**Merge**

**SM934-1 SM934-2 SM934-3**


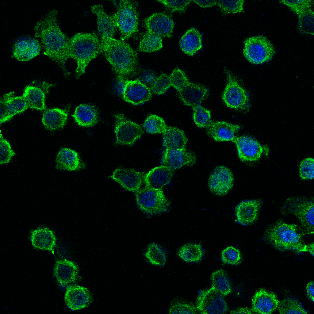

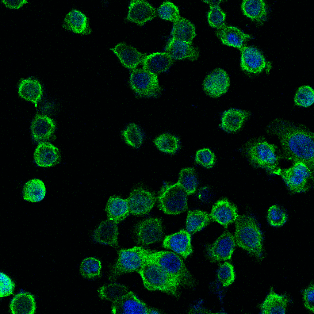

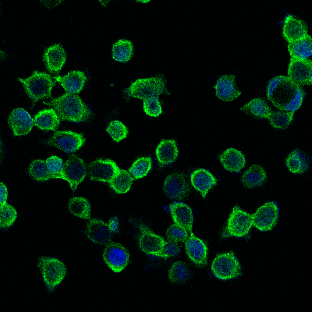

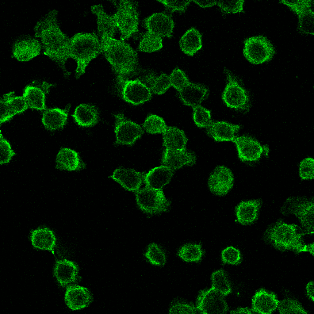

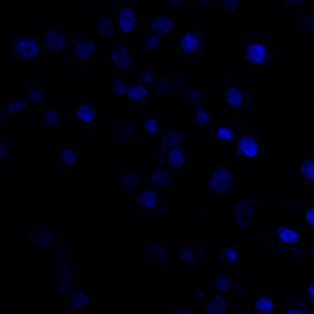

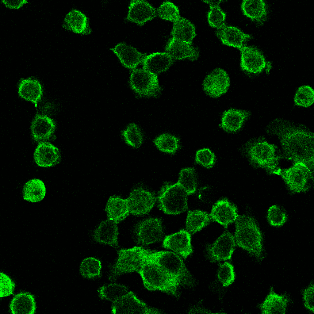

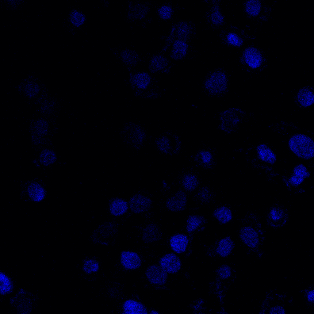

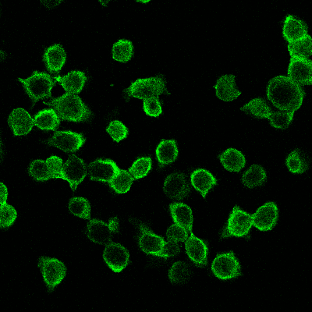

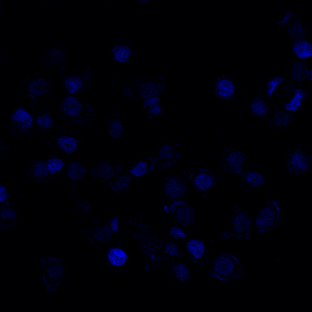


**DAPI**

**ZO-1**

**Merge**

**Control-1** **Control-2 Control-3**


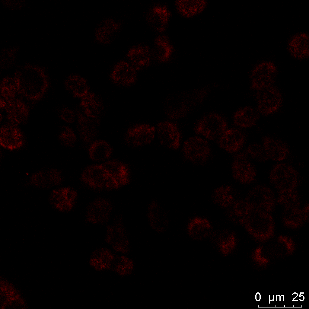


**DAPI**

**Cleaved**

**caspase-3**

**Merge**

**TNF-α-1** **TNF-α-2 TNF-α-3**

**DAPI**

**Cleaved**

**caspase-3**

**Merge**

**SM934-1 SM934-2 SM934-3**

**DAPI**

**Cleaved**

**caspase-3**

**Merge**

**Figure 5. Original** [**immunofluorescence**](javascript:;)**images (Three sample images of each group)**

**Normal**

**DAPI**

**Tunel**

**Merge**

**Vehicle**

**DAPI**

**Tunel**

**Merge**

**SM934 10mg/kg**

**DAPI**

**Tunel**

**Merge**

**Normal**

**DAPI**

**Caspase-1**

**Merge**

**Vehicle**

**DAPI**

**Caspase-1**

**Merge**

**SM934 10mg/kg**

**DAPI**

**Caspase-1**

**Merge**

**Figure 6. Original immunofluorescence cytochemistry images**

**Control-1 Control-2 Control-3**

**DAPI**

**NLRP3**

**Merge**

**LPS+ATP-1 LPS+ATP-2 LPS+ATP-3**

**DAPI**

**NLRP3**

**Merge**

**SM934-1 SM934-2 SM934-3**

**DAPI**

**NLRP3**

**Merge**

**Control-1 Control-2 Control-3**

**DAPI**

**Caspase-1**

**Merge**

**LPS+ATP-1 LPS+ATP-2 LPS+ATP-3**

**DAPI**

**Caspase-1**

**Merge**

**SM934-1 SM934-2 SM934-3**

**DAPI**

**Caspase-1**

**Merge**

**Control-1 Control-2 Control-3**

**DAPI**

**GSDMD**

**Merge**

**LPS+ATP-1 LPS+ATP-2 LPS+ATP-3**

**DAPI**

**GSDMD**

**Merge**

**SM934-1 SM934-2 SM934-3**

**DAPI**

**GSDMD**

**Merge**
