# Supplementary material for: Artemisinin analog SM934 alleviates epithelial barrier dysfunction via inhibiting apoptosis and caspase-1-mediated pyroptosis in experimental colitis
Source: Front Pharmacol. 2022 Sep 1;13:849014. doi: 10.3389/fphar.2022.849014 (PMC9477143; doi:10.3389/fphar.2022.849014)
Supplement: Supplementary file 2 [file DataSheet1.docx]

**
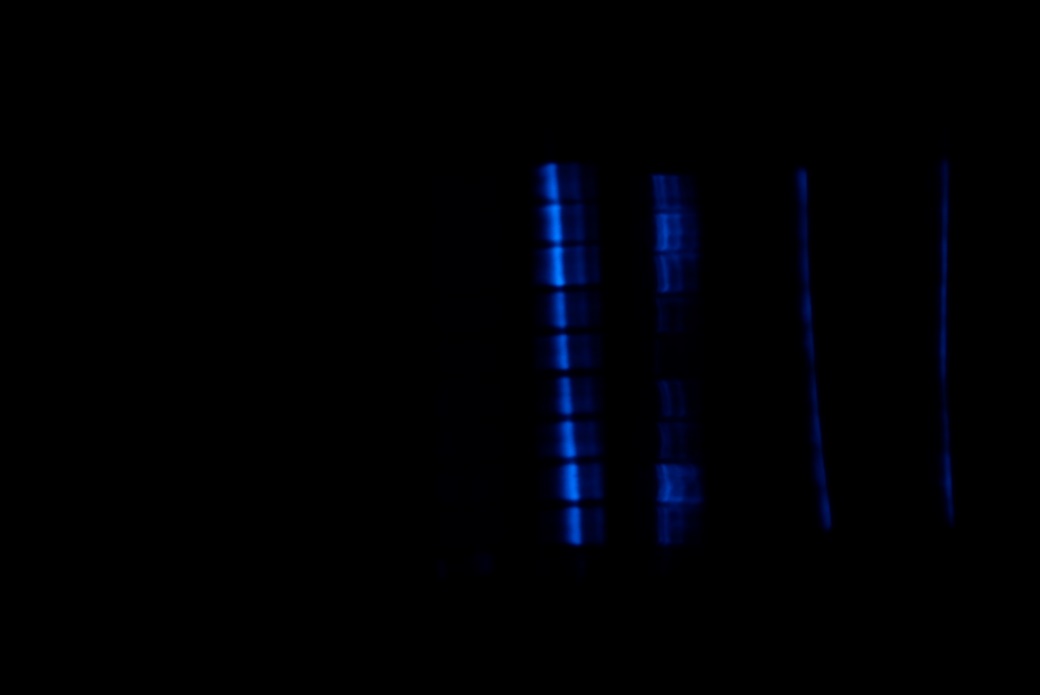
Figure 2. Original western blot**



**


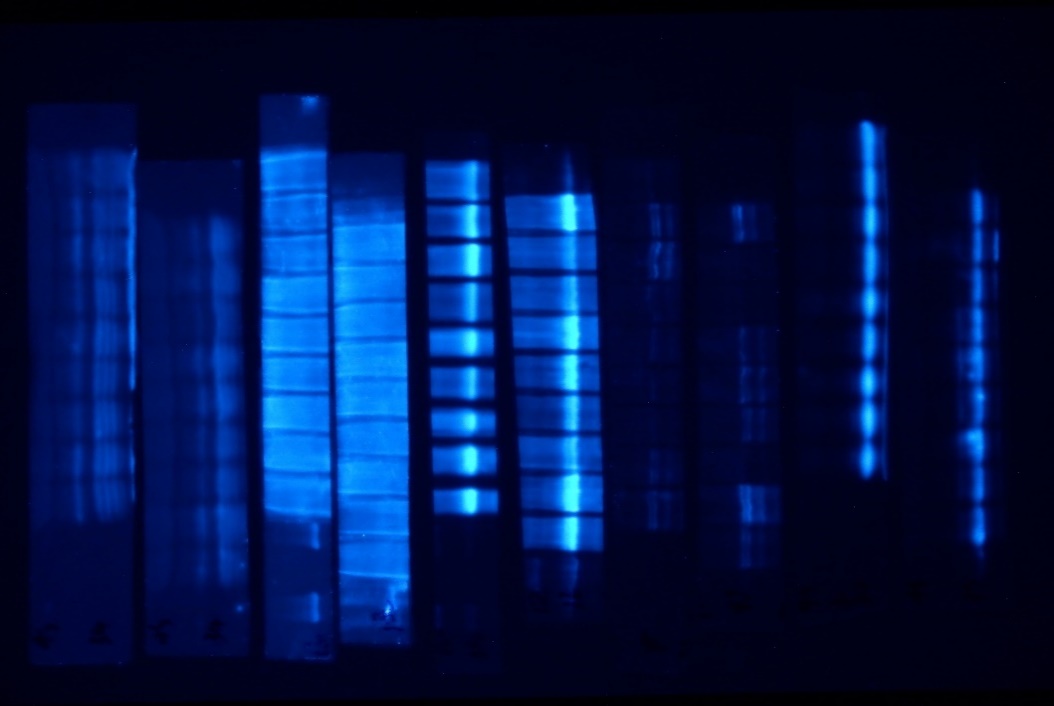

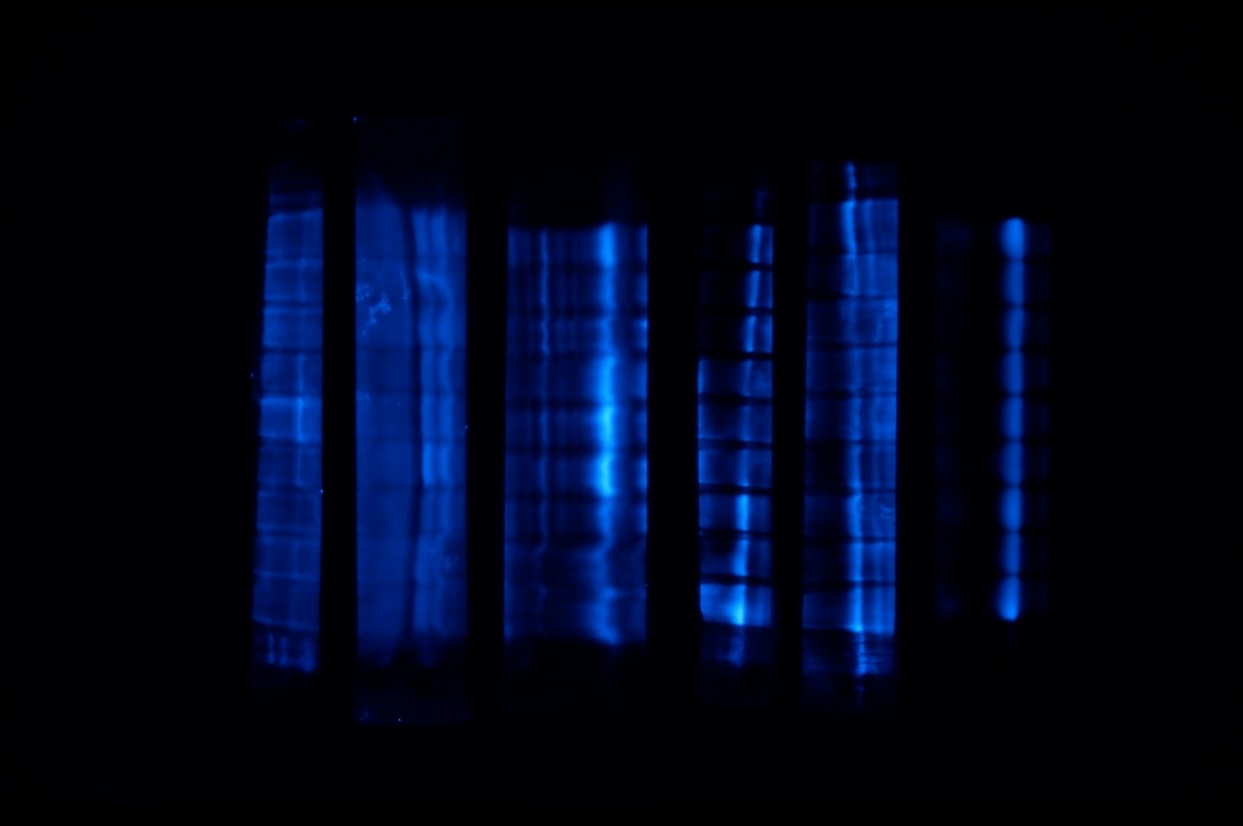


**

**E-cadherin 135 kDa**

**β-catenin 92 kDa**

**GAPDH 37 kD**

**E-cadherin 135 kDa**

**GAPDH 37 kD**

**β-catenin 92 kDa**

**Occludin 59 kDa**





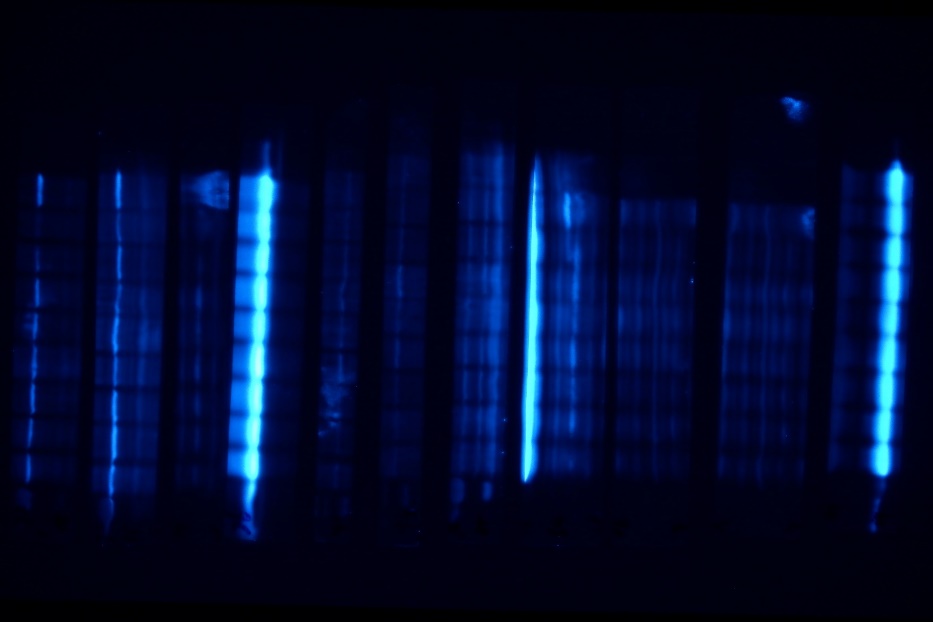


**GAPDH 37 kD**

**Figure 3. Original western blot**

**

**

**BAX 20 kDa**

**
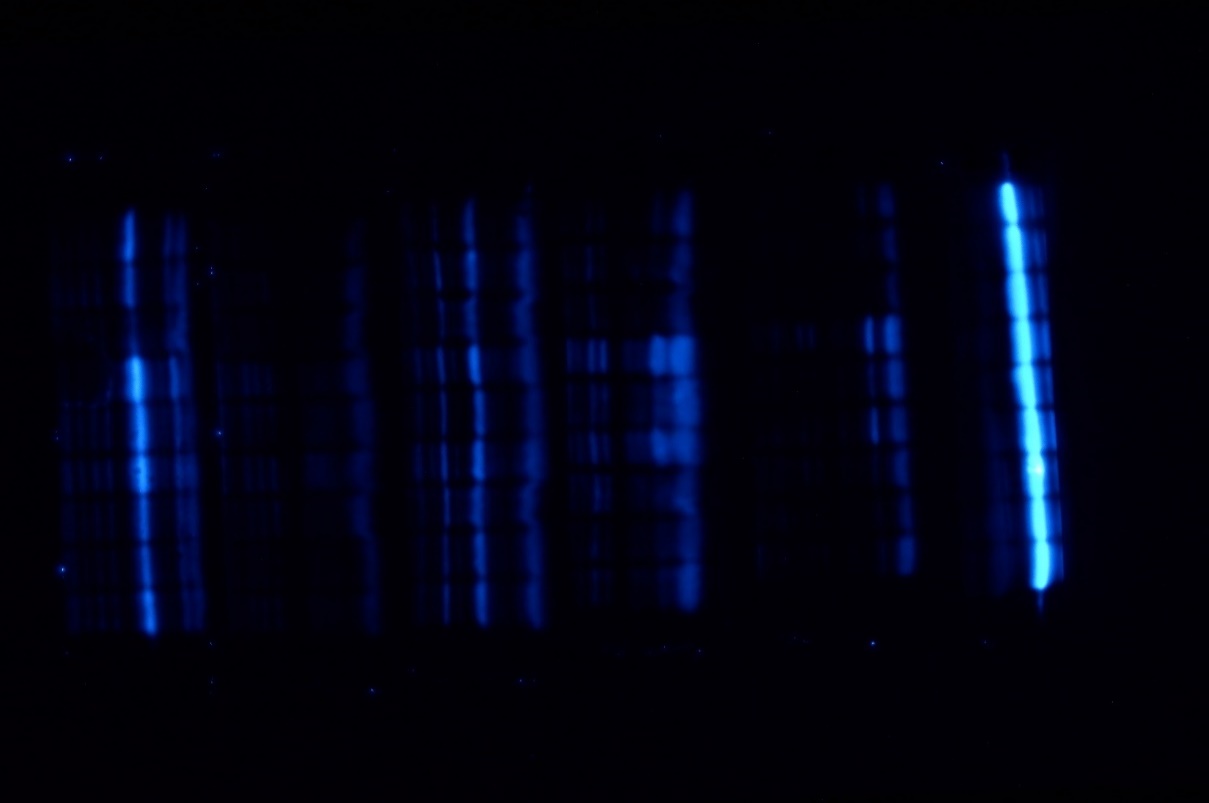
**

**Cleaved caspase-3 17, 19 kDa**

**Cleaved caspase-3 17, 19 kDa**

**GAPDH 37 kDa**

**
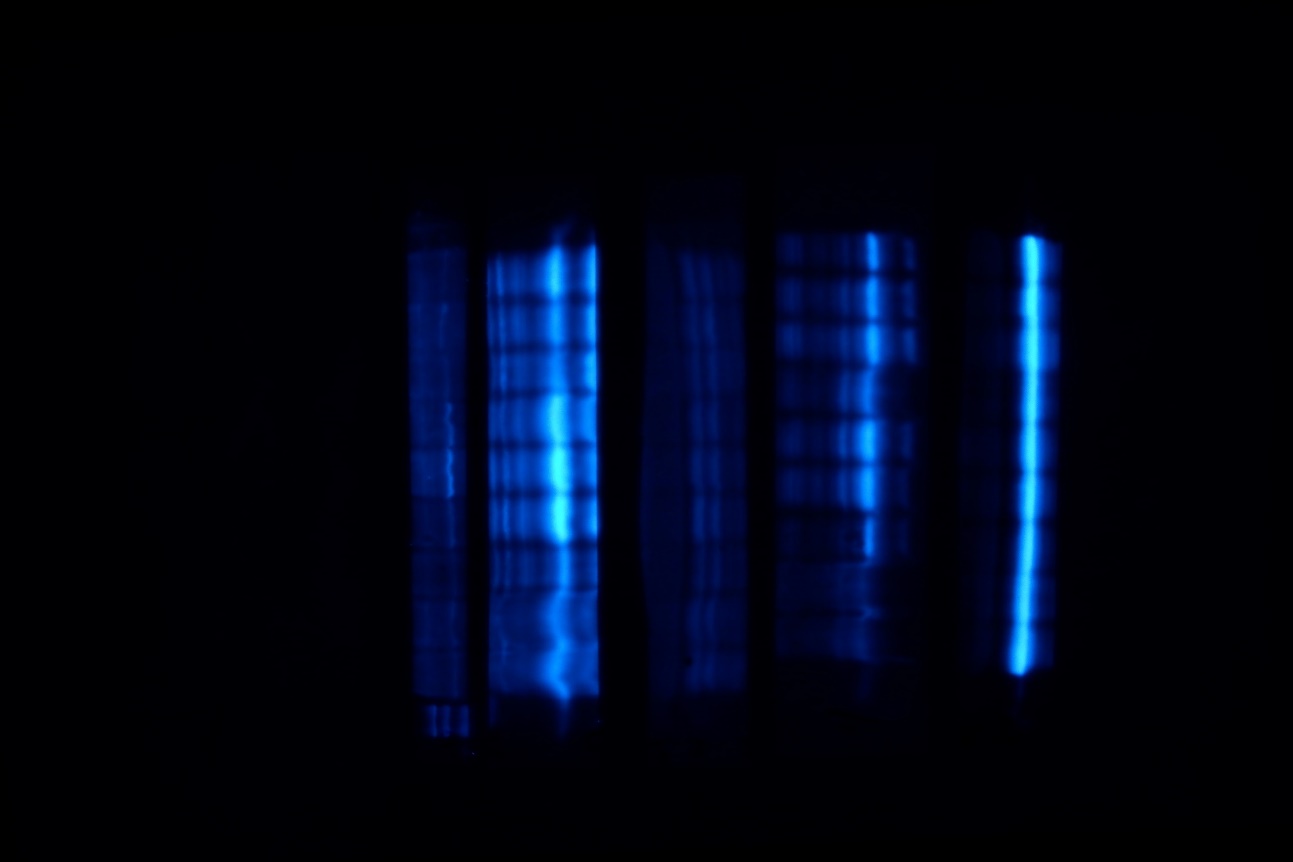
**

**Cleaved caspase-9 37 kDa**

**

**

**Cleaved caspase-3 17, 19 kDa**

**GAPDH 37 kDa**





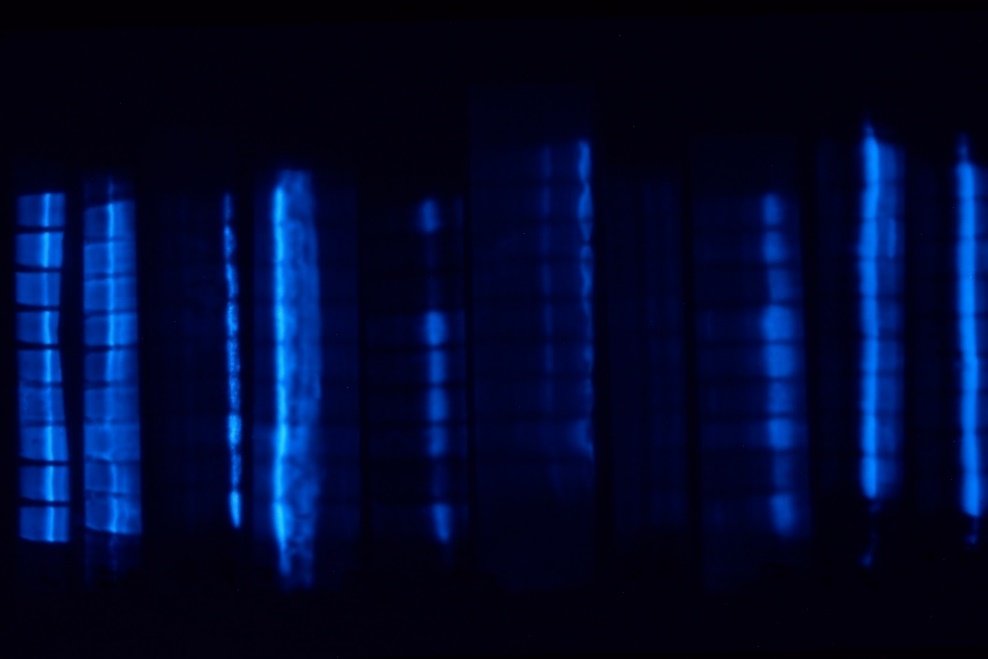


**GAPDH 37 kDa**

**Cleaved caspase-3 17, 19 kDa**

**

**
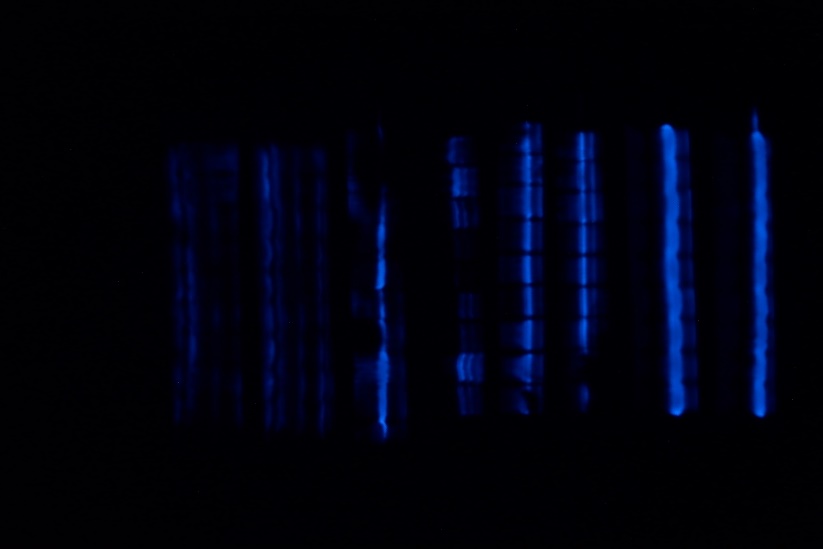


**Bcl-2 26 kDa**

**GAPDH 37 kDa**

**Figure 4. Original western blot**

**
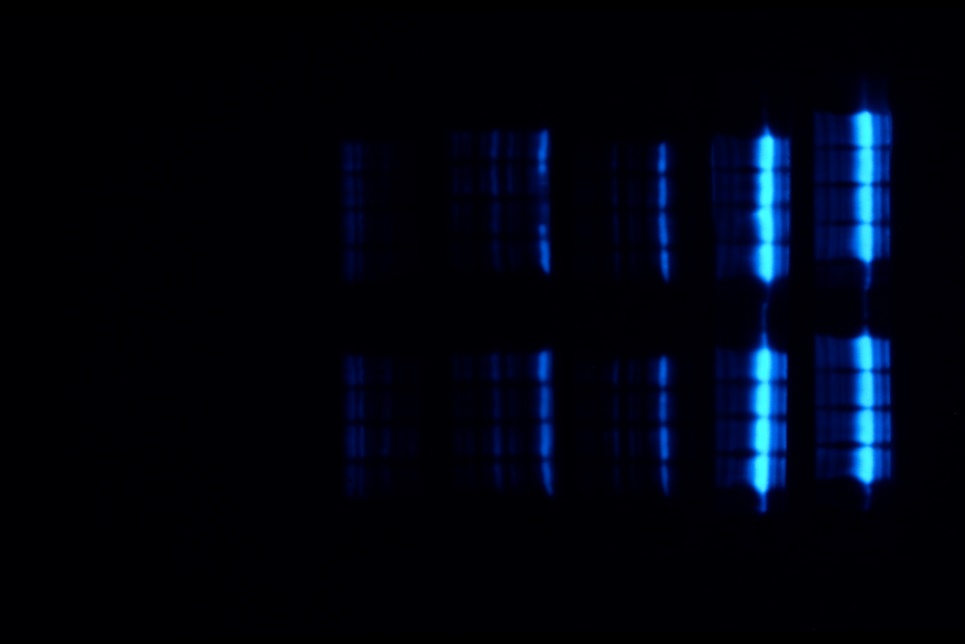


**

**Cleaved caspase-3 17, 19 kDa**

**Cleaved caspase-3 17, 19 kDa**

**GAPDH 37 kDa**

**GAPDH 37 kDa**

**

**

**
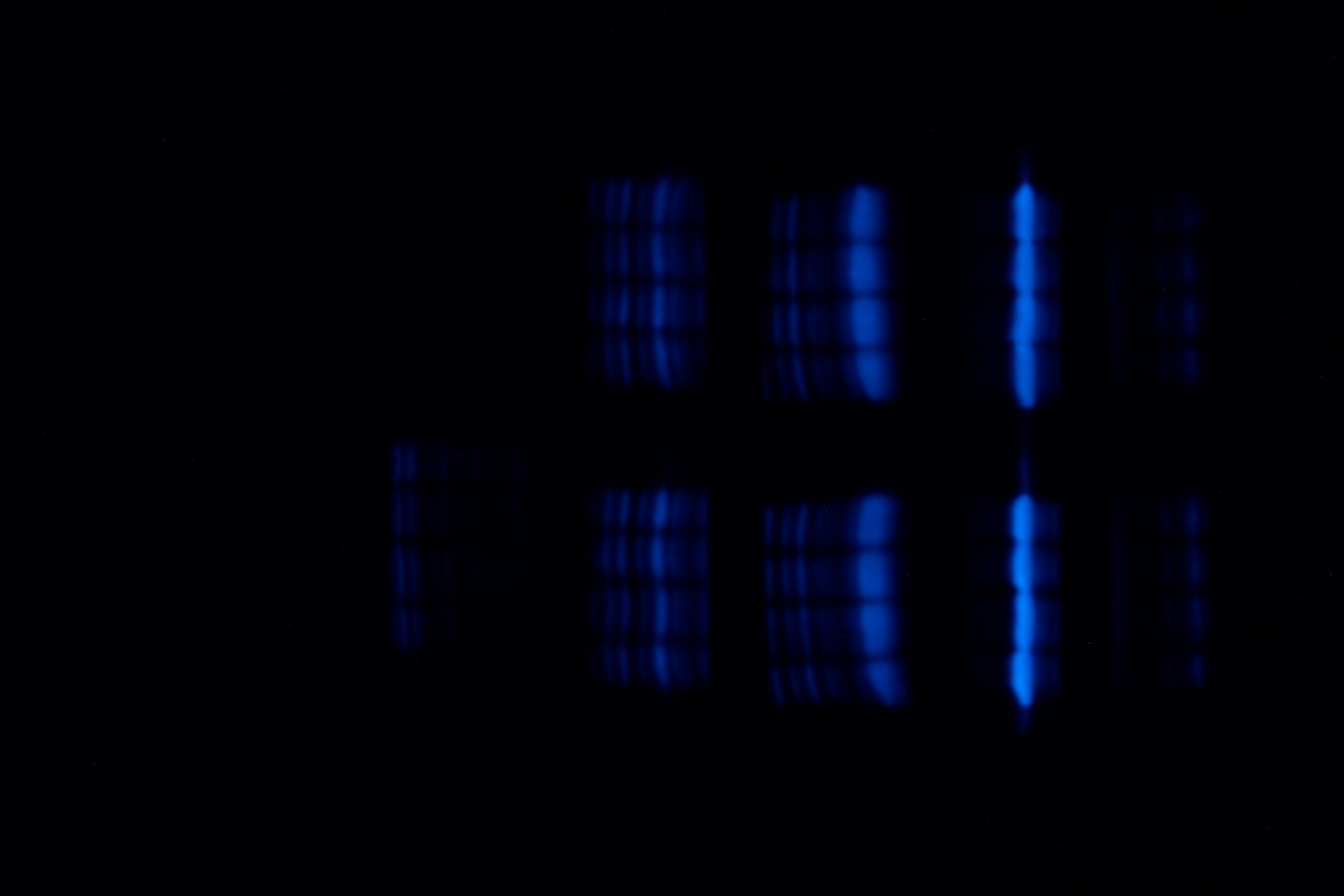
**

**Cleaved caspase-3 17, 19 kDa**

**Cleaved caspase-3 17, 19 kDa**

**GAPDH 37 kDa**





**ZO-1 189 kDa**

**
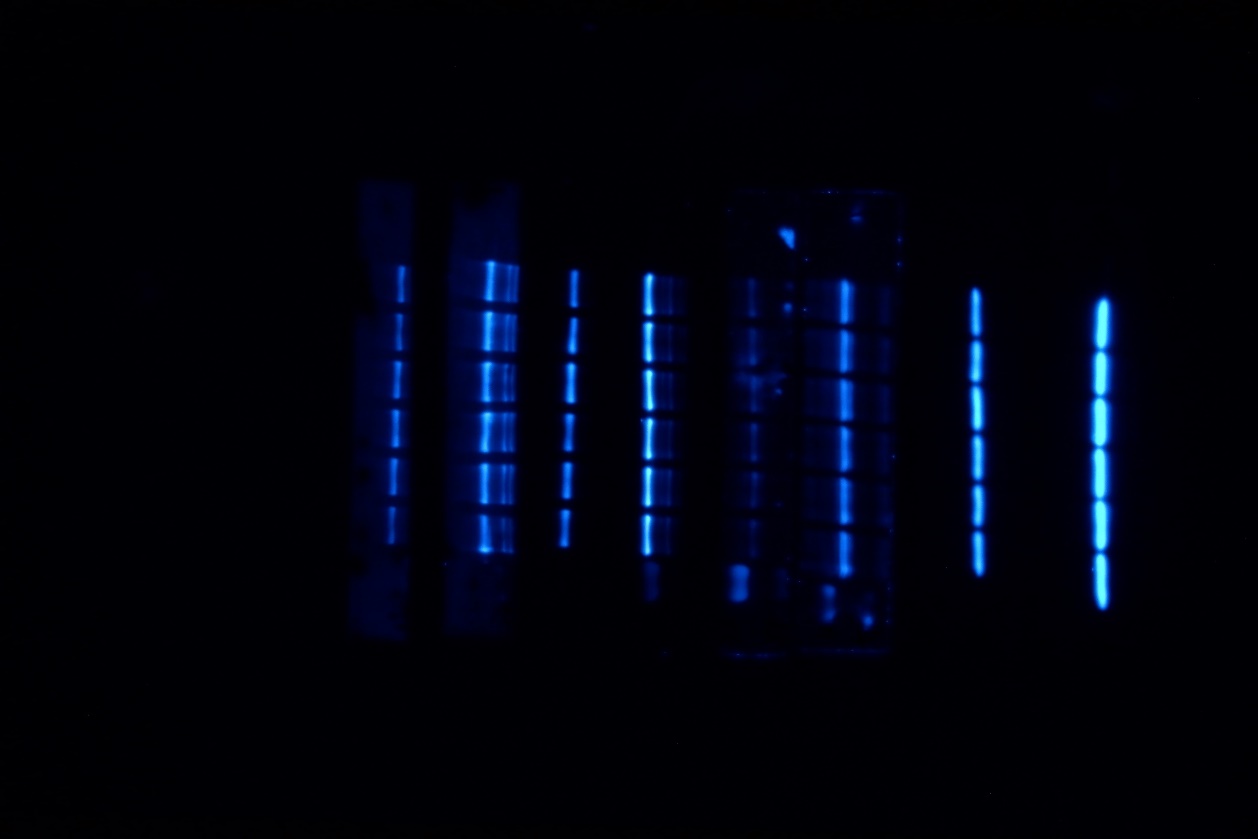
**

**E-cadherin 135 kDa**

**GAPDH 37 kDa**





**ZO-1 189 kDa**

**
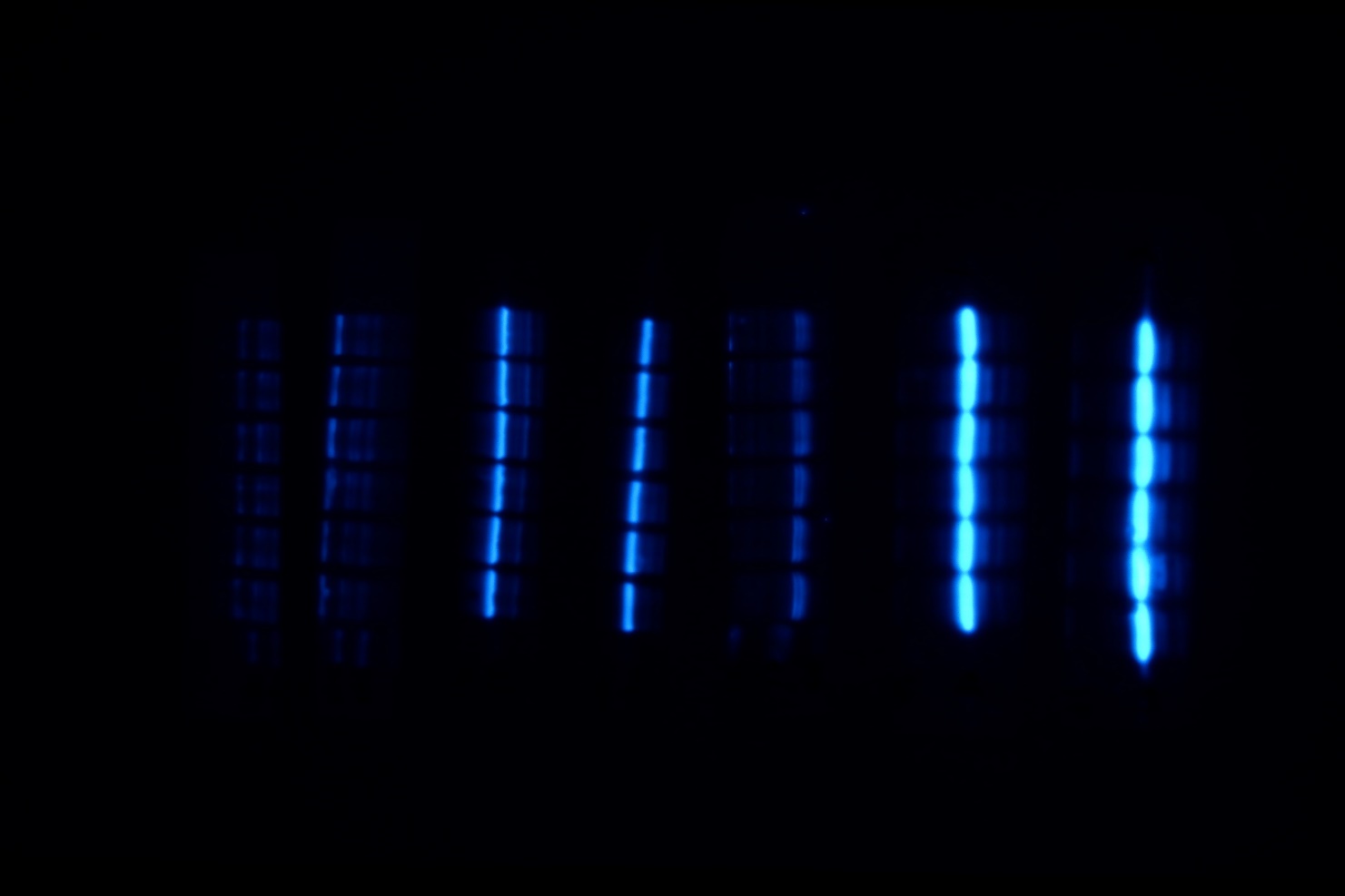
**

**E-cadherin 135 kDa**

**GAPDH 37 kDa**

**

**

**ZO-1 189 kDa**

**
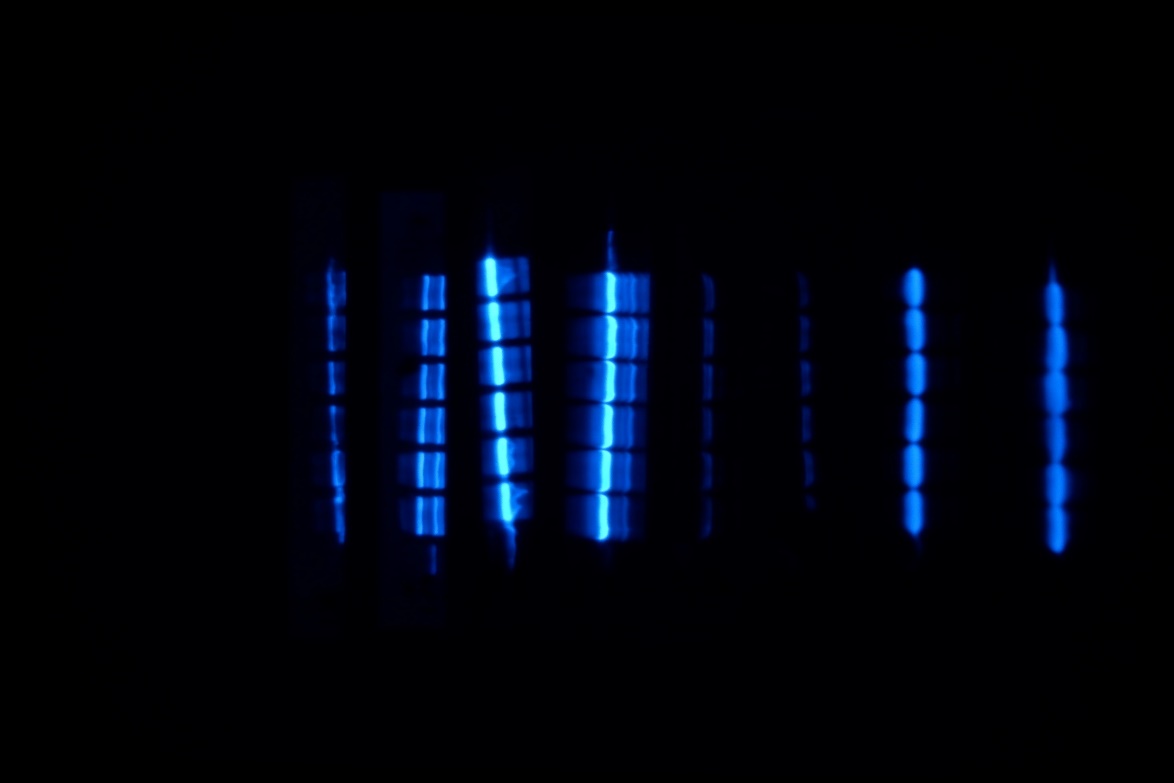
**

**E-cadherin 135 kDa**

**GAPDH 37 kDa**

**
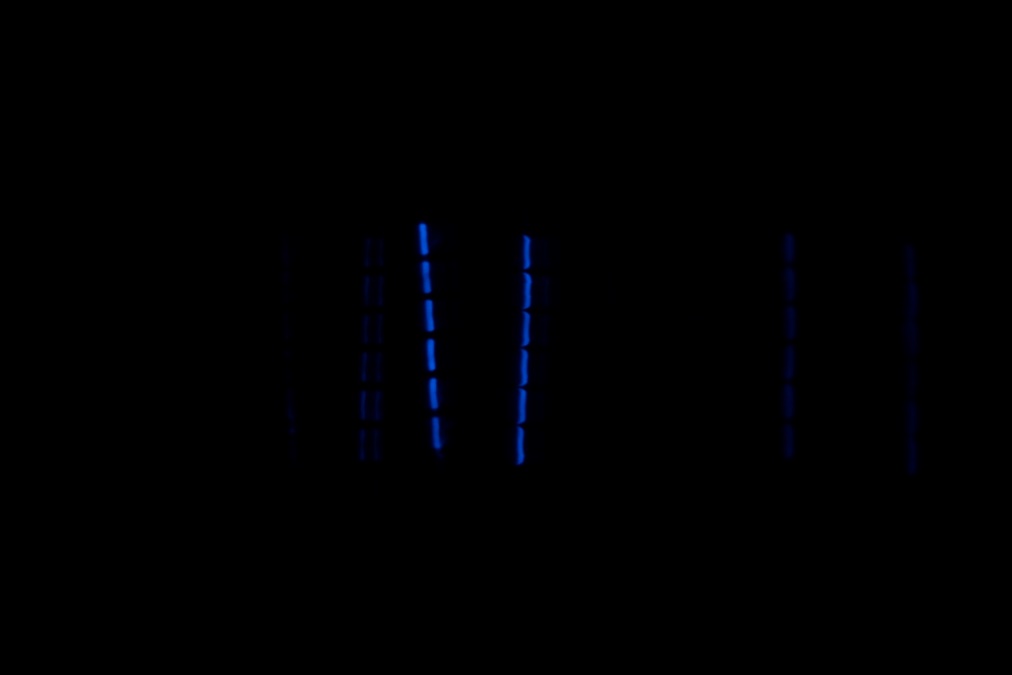
**

**

**

**E-cadherin 135 kDa**

**Figure 5. Original western blot**





**NLRP3 110 kDa**


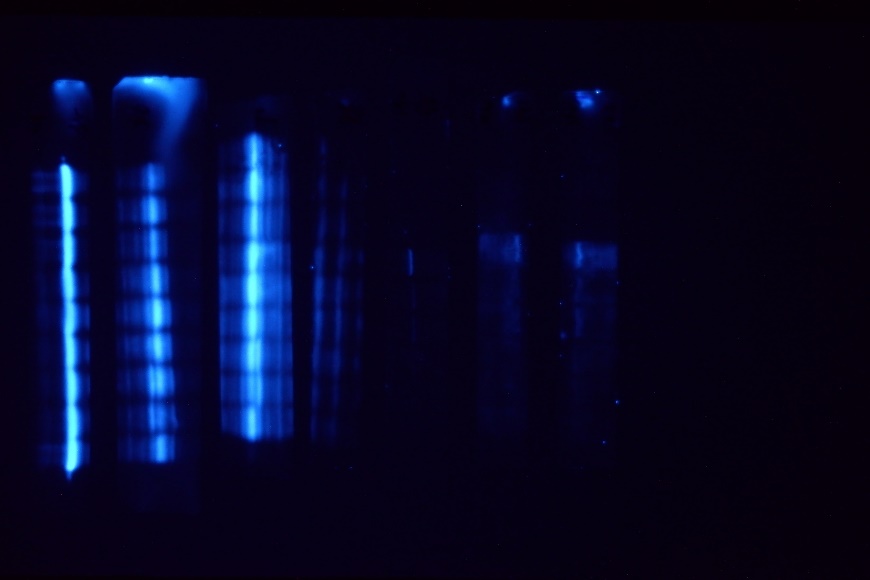


**ACS 22 kDa**

**ACS 22 kDa**

**GAPDH 37 kDa**


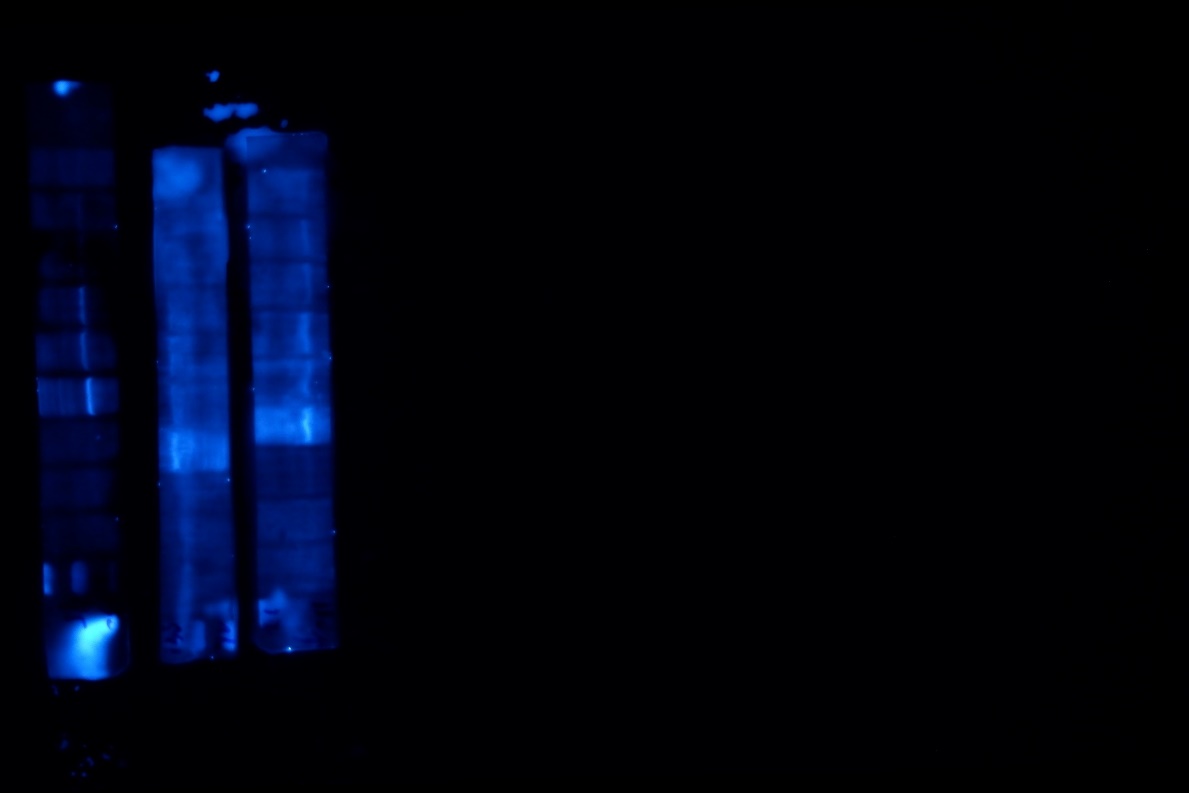


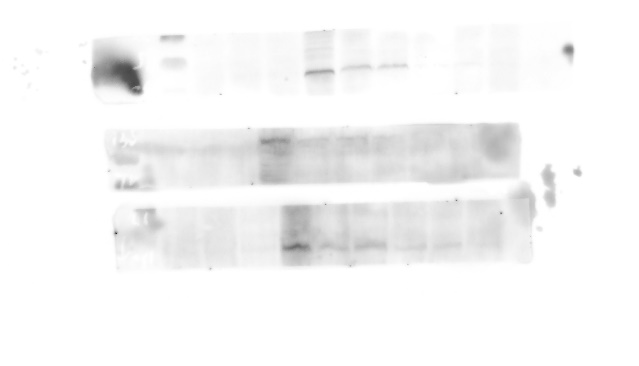


**NLRP3 110 kDa**





**Cleaved caspase-1 22 kDa**


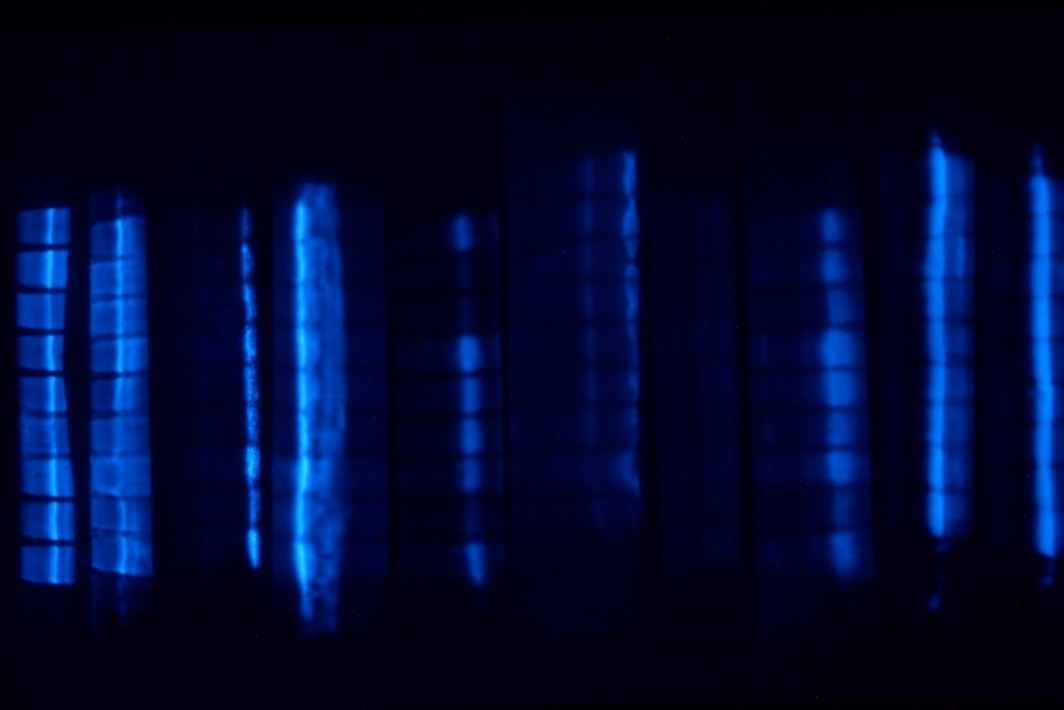


**GAPDH 37 kDa**

**GAPDH 37 kDa**





**NLRP3 110 kDa**


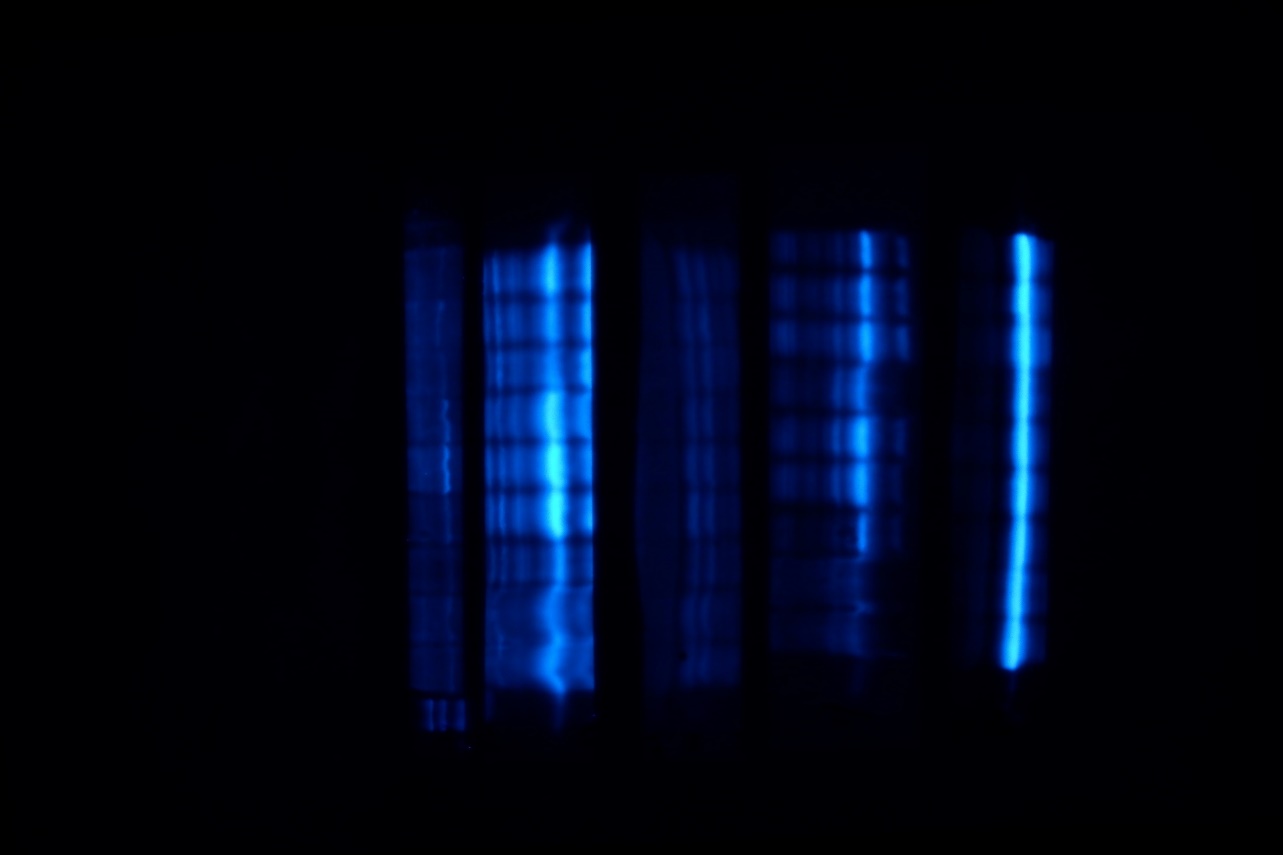


**Cleaved caspase-1 22 kDa**

**GAPDH 37 kDa**





**F-GSDMD 50-70 kDa**


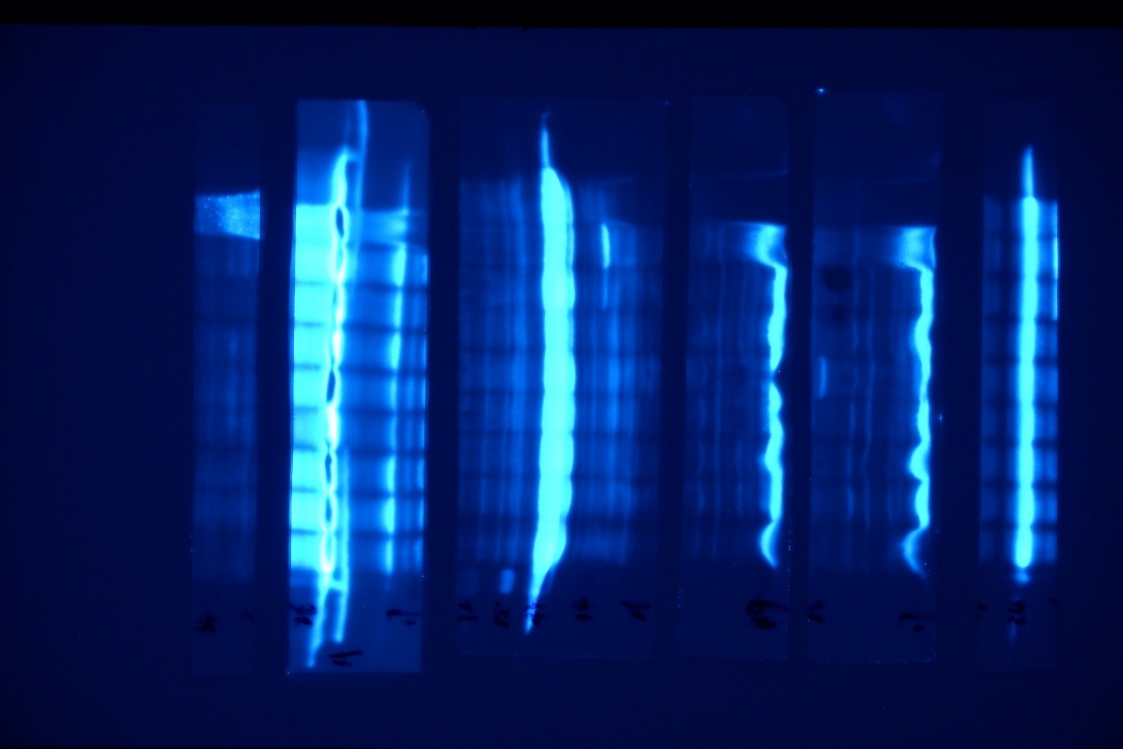


**N-GSDMD 25-35 kDa**

**GAPDH 37 kDa**





**HMGB1 29 kDa**


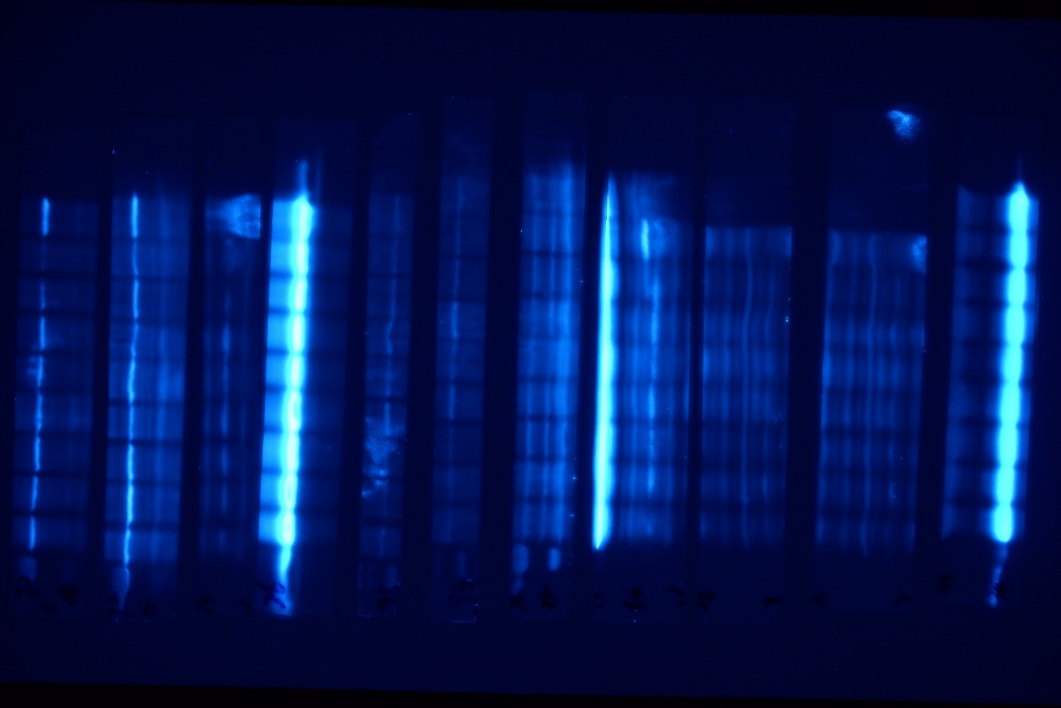


**NLRP3 110 kDa**

**N-GSDMD 50-70 kDa**

**N-GSDMD 25-35 kDa**

**IL-18 22 kDa**

**GAPDH 37 kDa**





**IL-18 22 kDa**


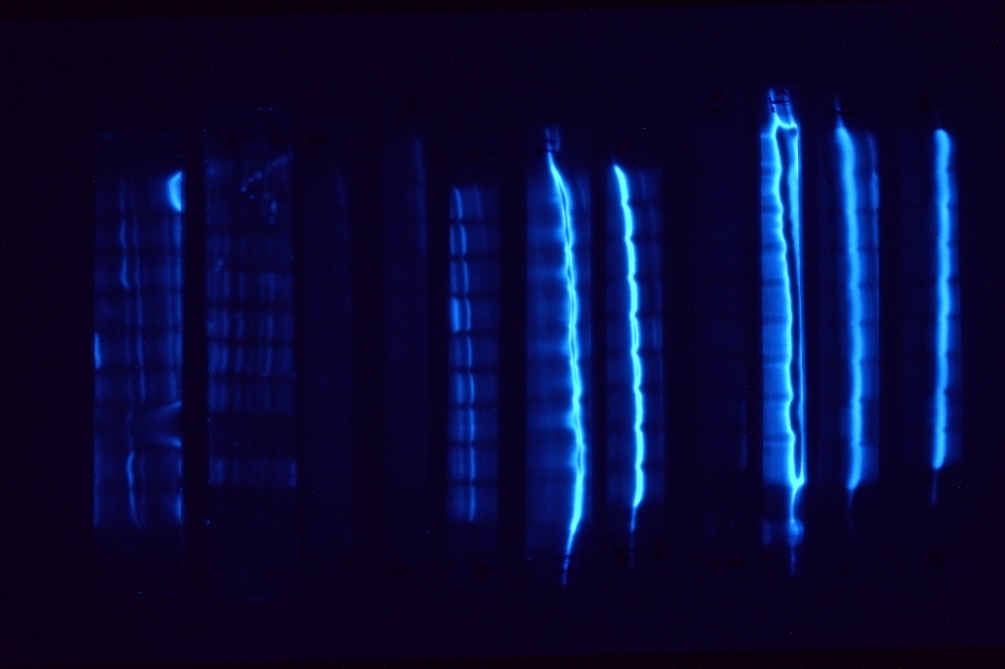


**GAPDH 37 kDa**

**Figure 6. Original western blot**

**


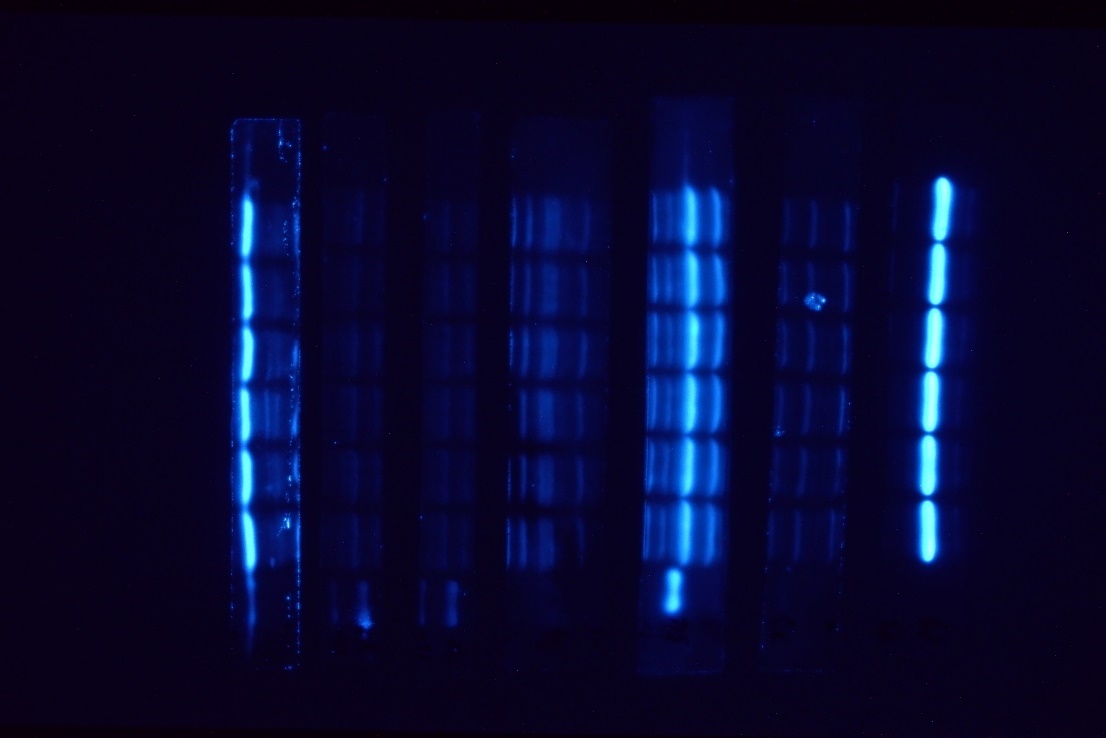
**

**NLRP3 110 kDa**

**F-GSDMD 50-70 kDa**

**IL-18 22 kDa**

**GAPDH 37 kDa**

**
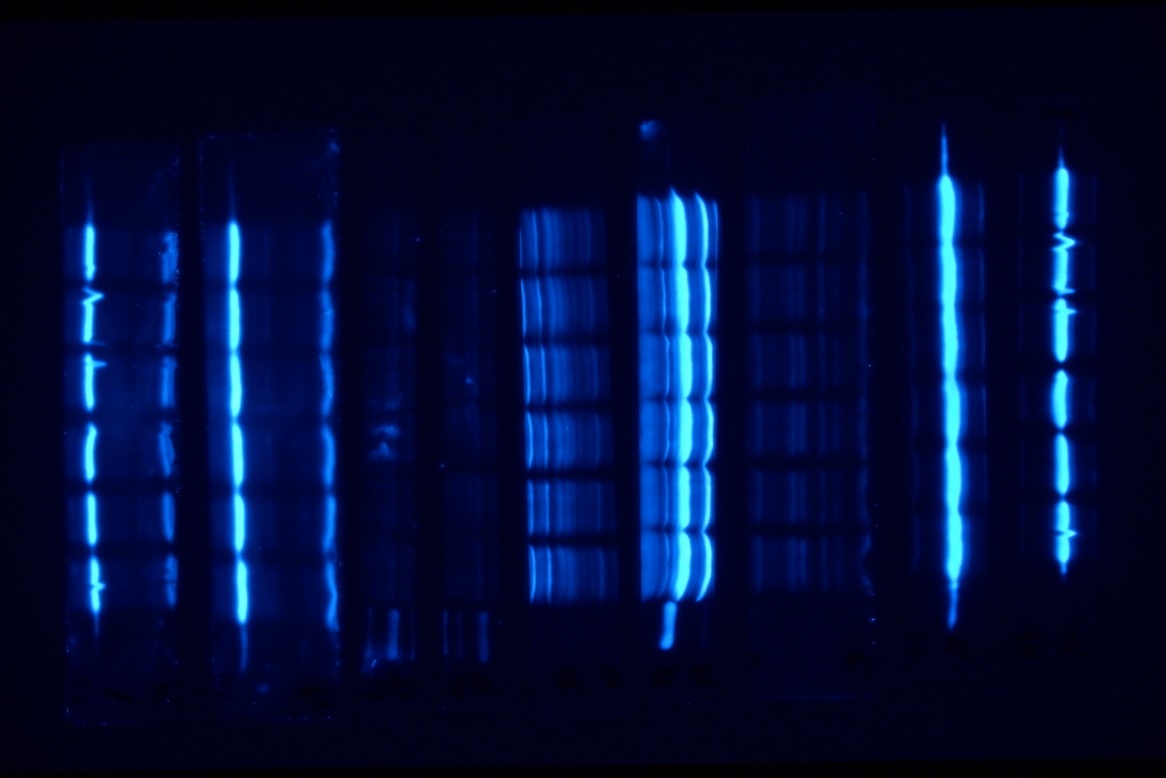
**

**

**

**N-GSDMD 25-35 kDa**

**F-GSDMD 50-70 kDa**

**IL-18 22 kDa**





**NLRP3 110 kDa**


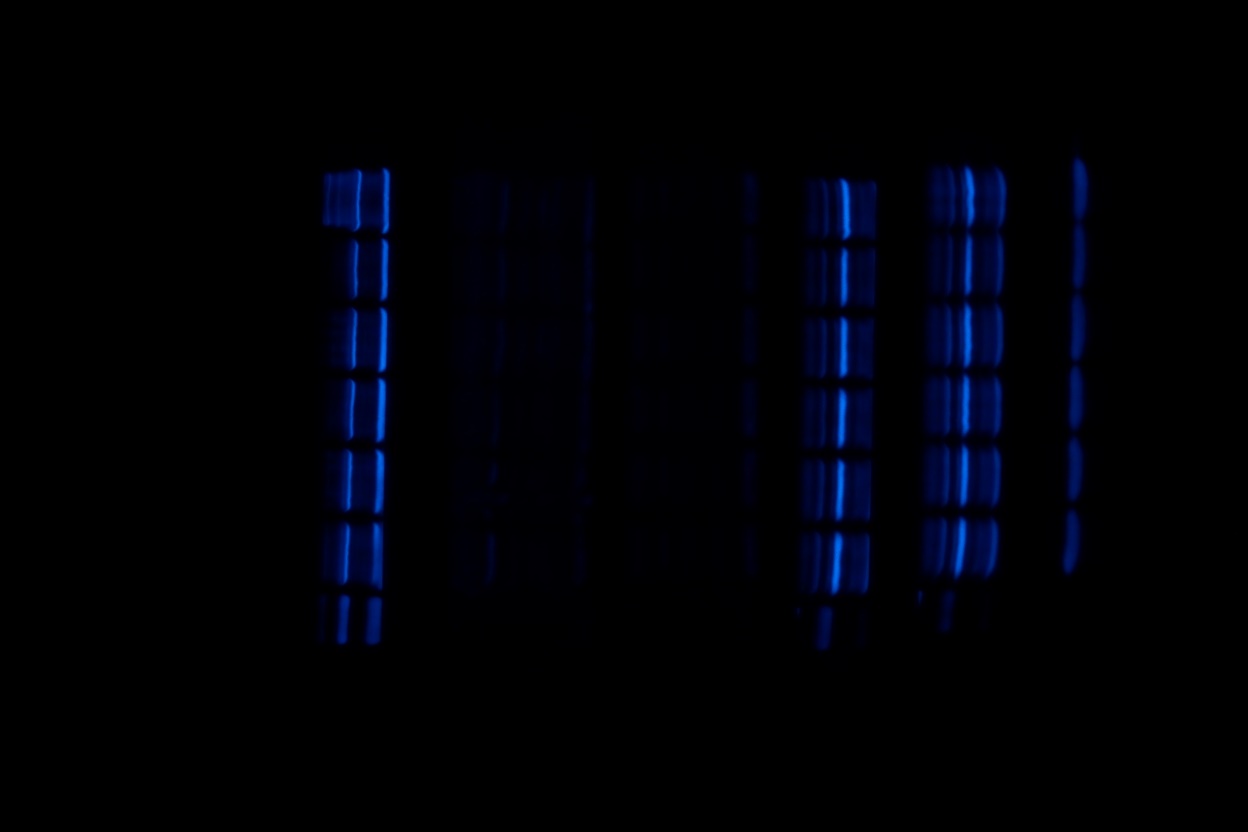


**GAPDH 37 kDa**

**

**

**ASC 22 kDa**

**
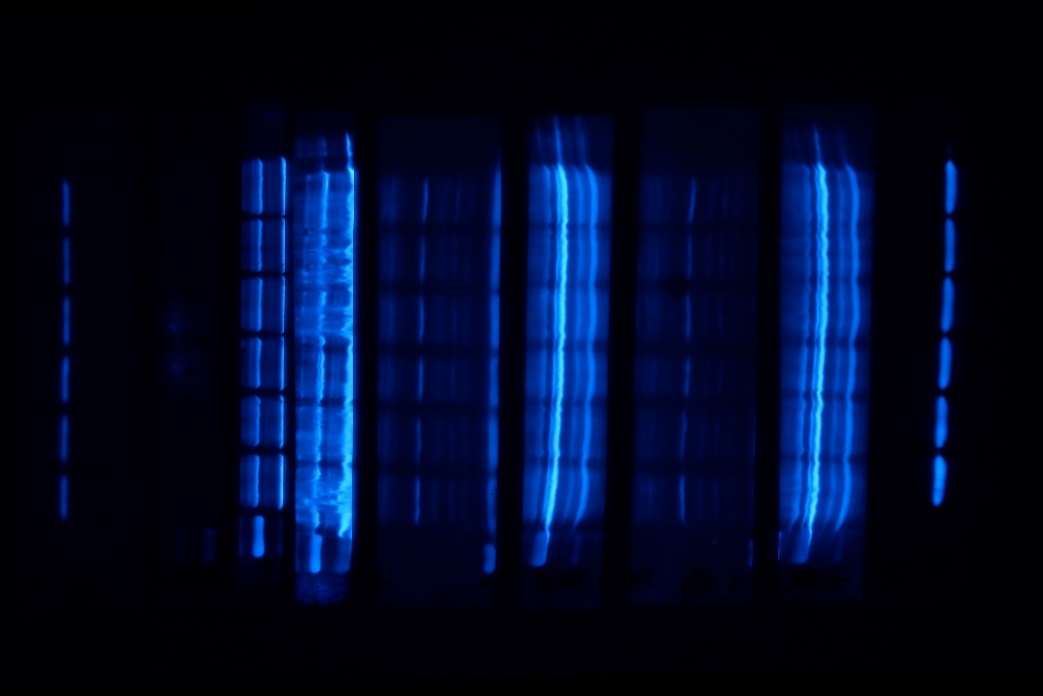
**

**Cleaved caspase-1 20-25 kDa**

**Pro-caspase-1 48 kDa**

**GAPDH 37 kDa**

**

**

**ASC 22 kDa**

**NLRP3 110 kDa**

**
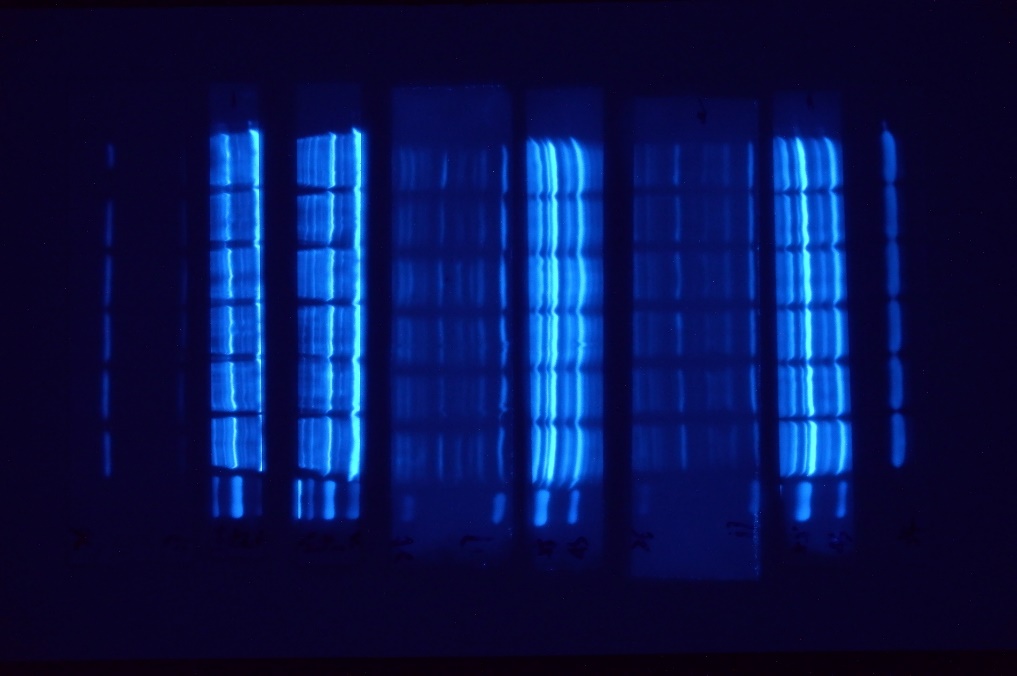
**

**Cleaved caspase-1 20-25 kDa**

**Pro-caspase-1 48 kDa**

**Cleaved caspase-1 20-25 kDa**

**Pro-caspase-1 48 kDa**

**GAPDH 37 kDa**

**Figure 7. Original western blot**


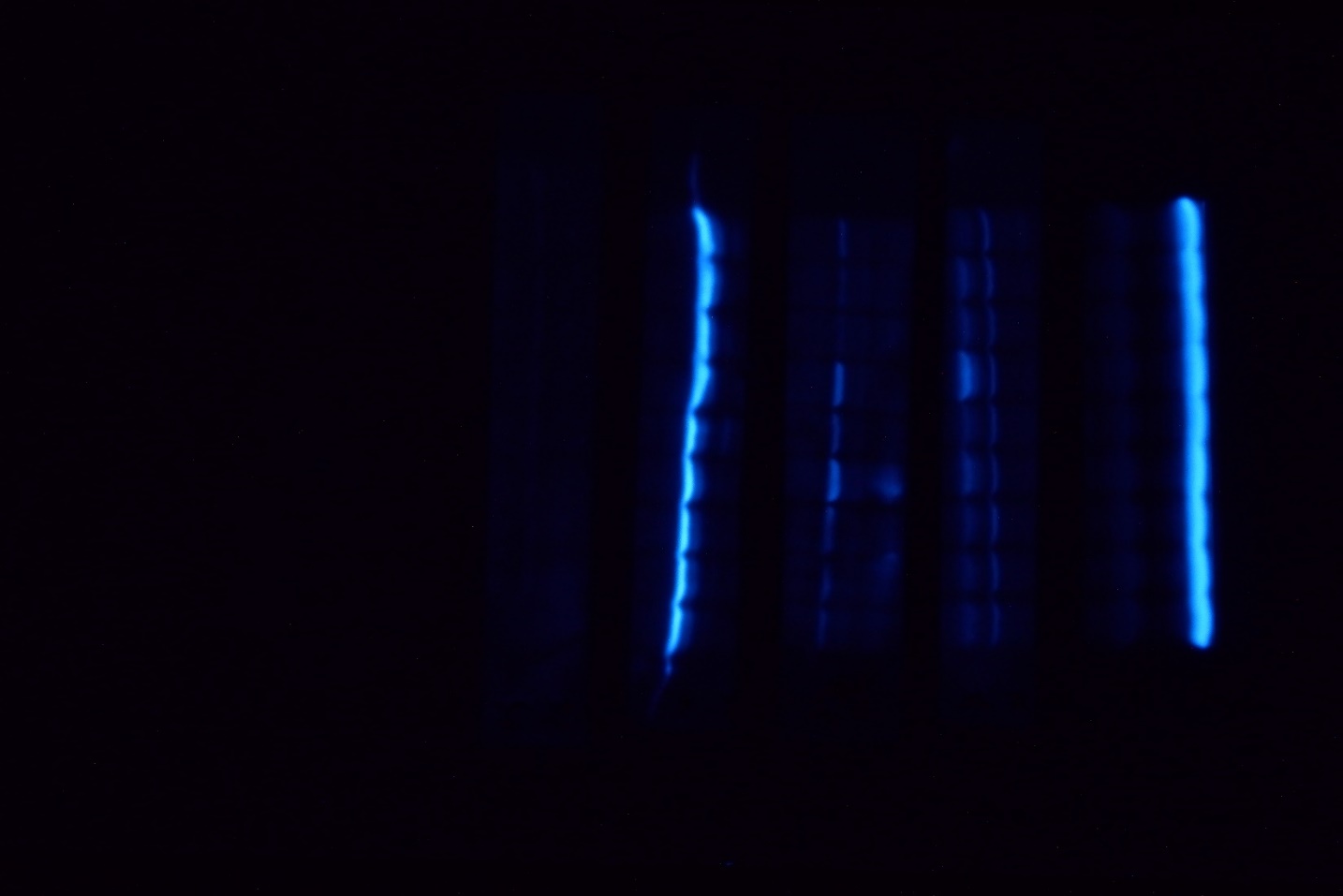





**p-NF- kB 65 kDa**

**NF- kB 65 kDa**

**GAPDH 37 kDa**


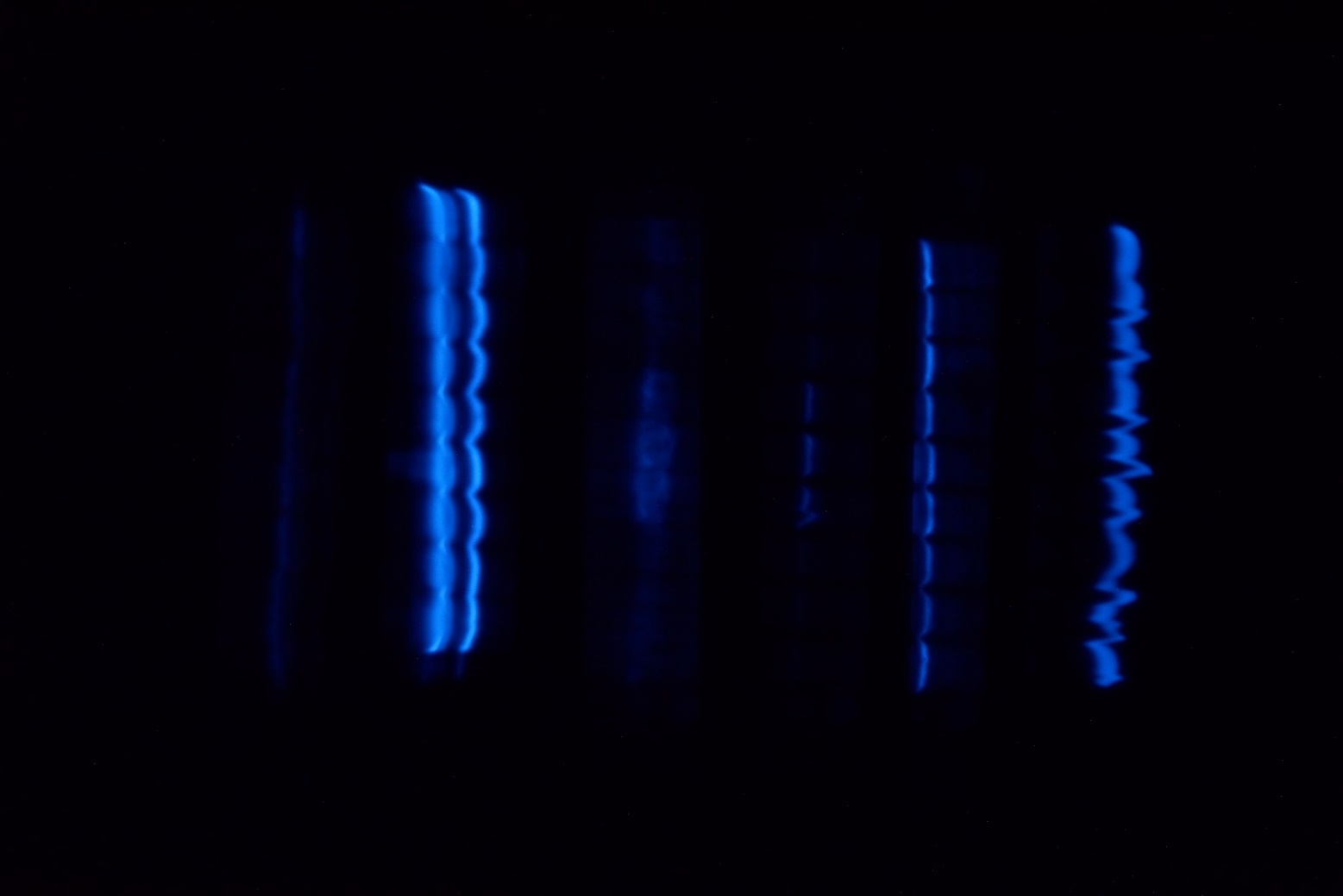


**p-NF- kB 65 kDa**





**NF- kB 65 kDa**

**GAPDH 37 kDa**


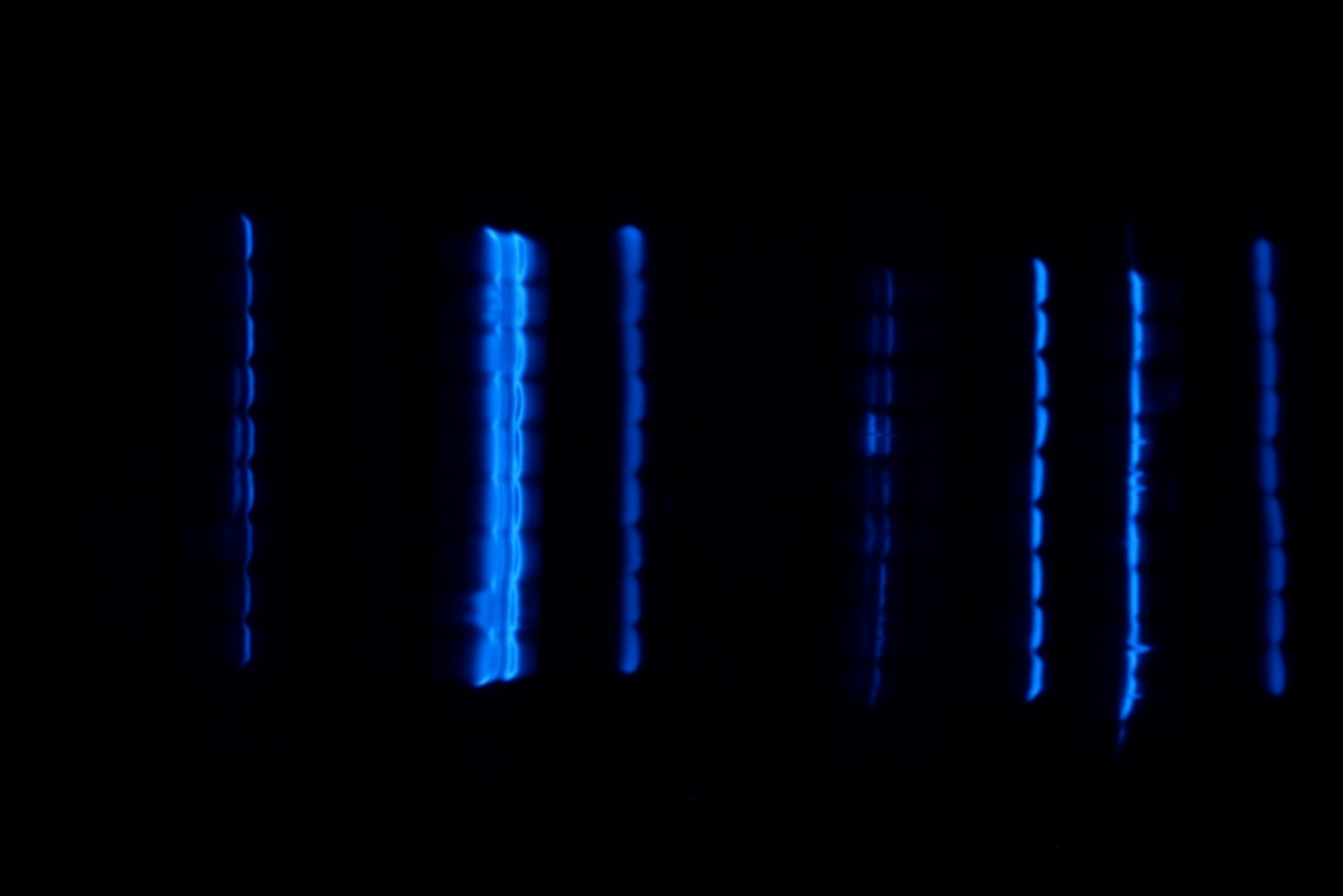





**p38 kDa 40**

**p-p38 kDa 43**

**GAPDH 37 kDa**





**p-ERK 42,44 kDa**


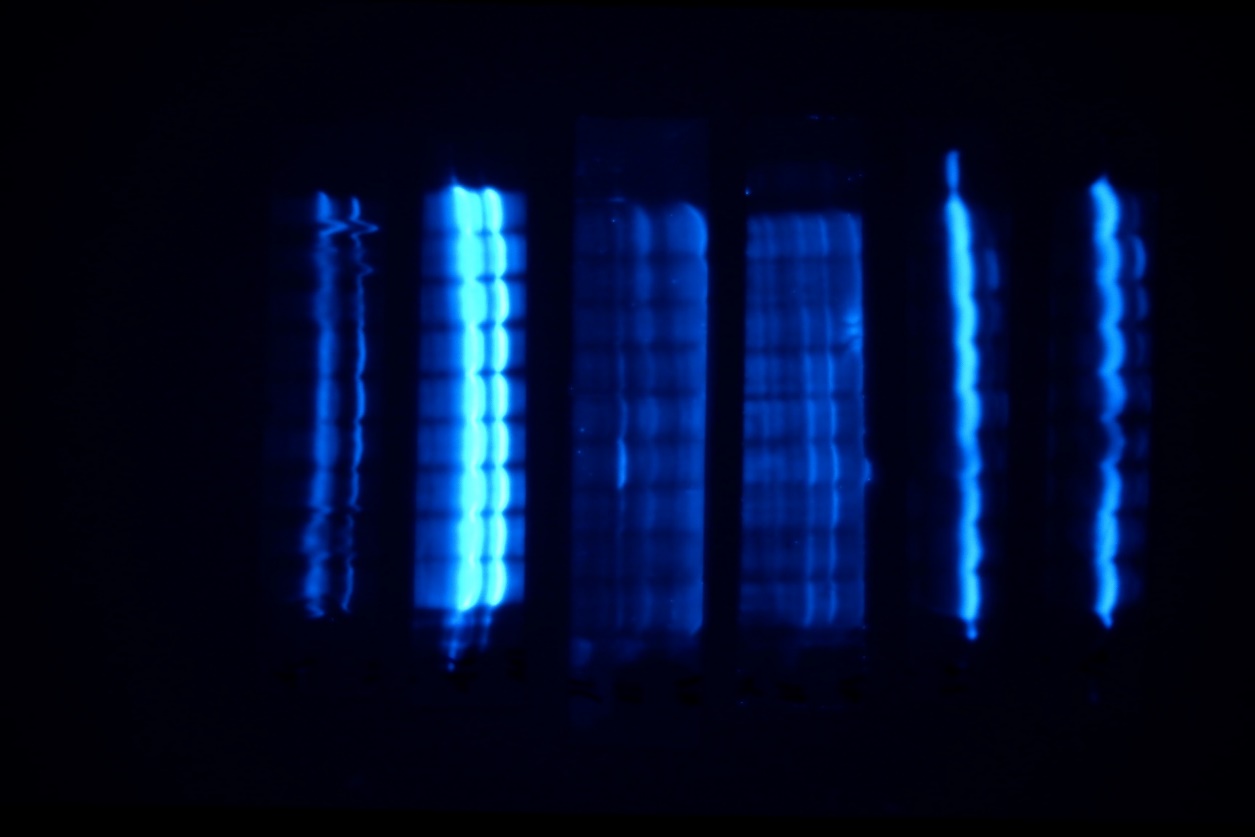


**ERK 42,44 kDa**

**GAPDH 37 kDa**





**p-ERK 42,44 kDa**

**ERK 42,44 kDa**


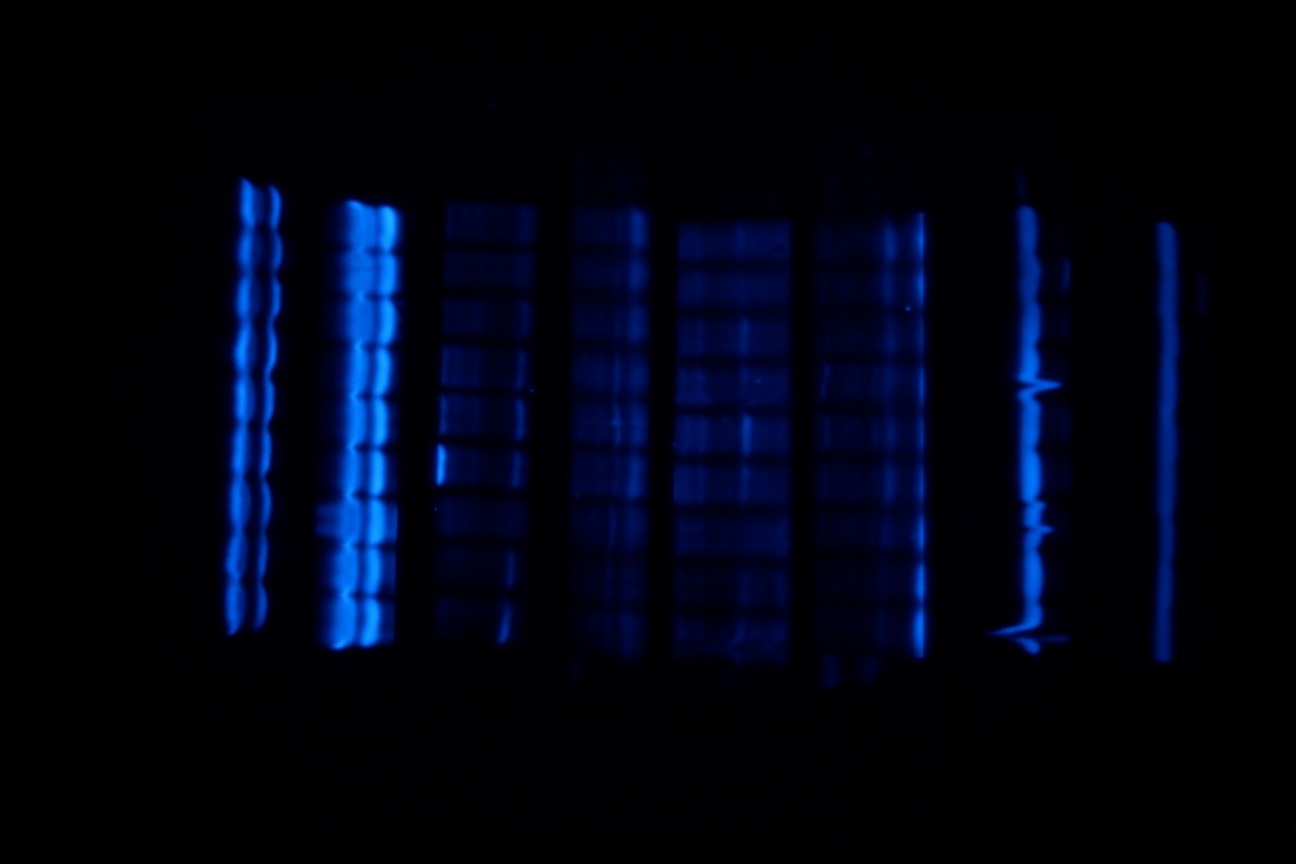


**GAPDH 37 kDa**

**GAPDH 37 kDa**




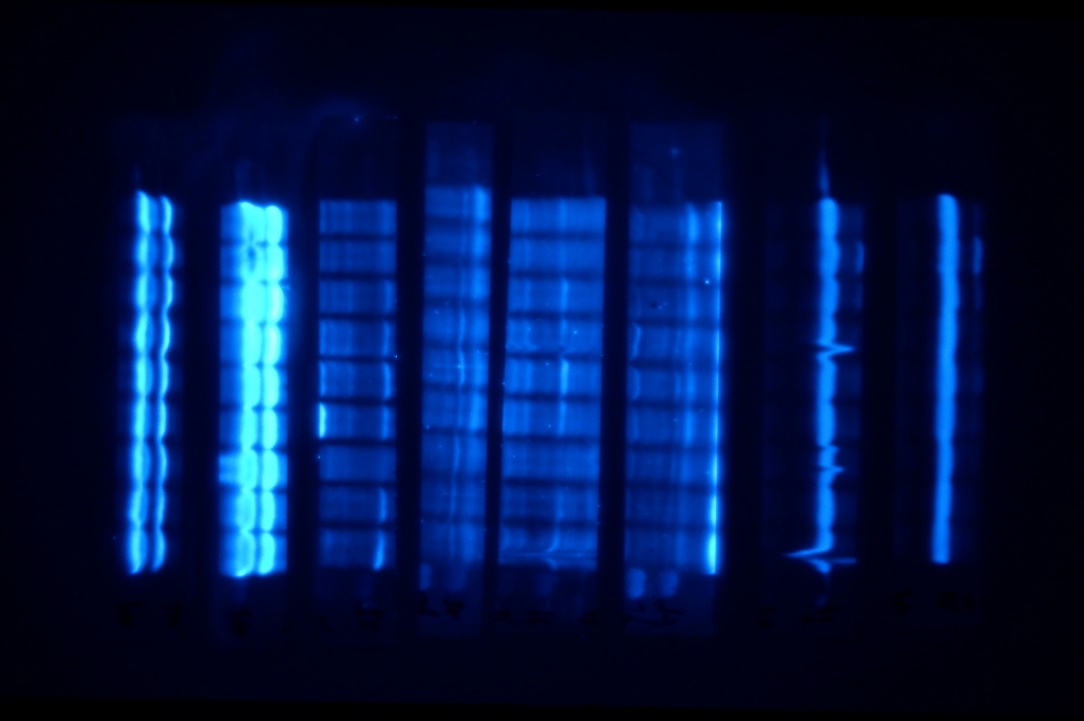


**p-JNK 46, 54 kDa**

**JNK 46, 54 kDa**

**GAPDH 37 kDa**

**GAPDH 37 kDa**


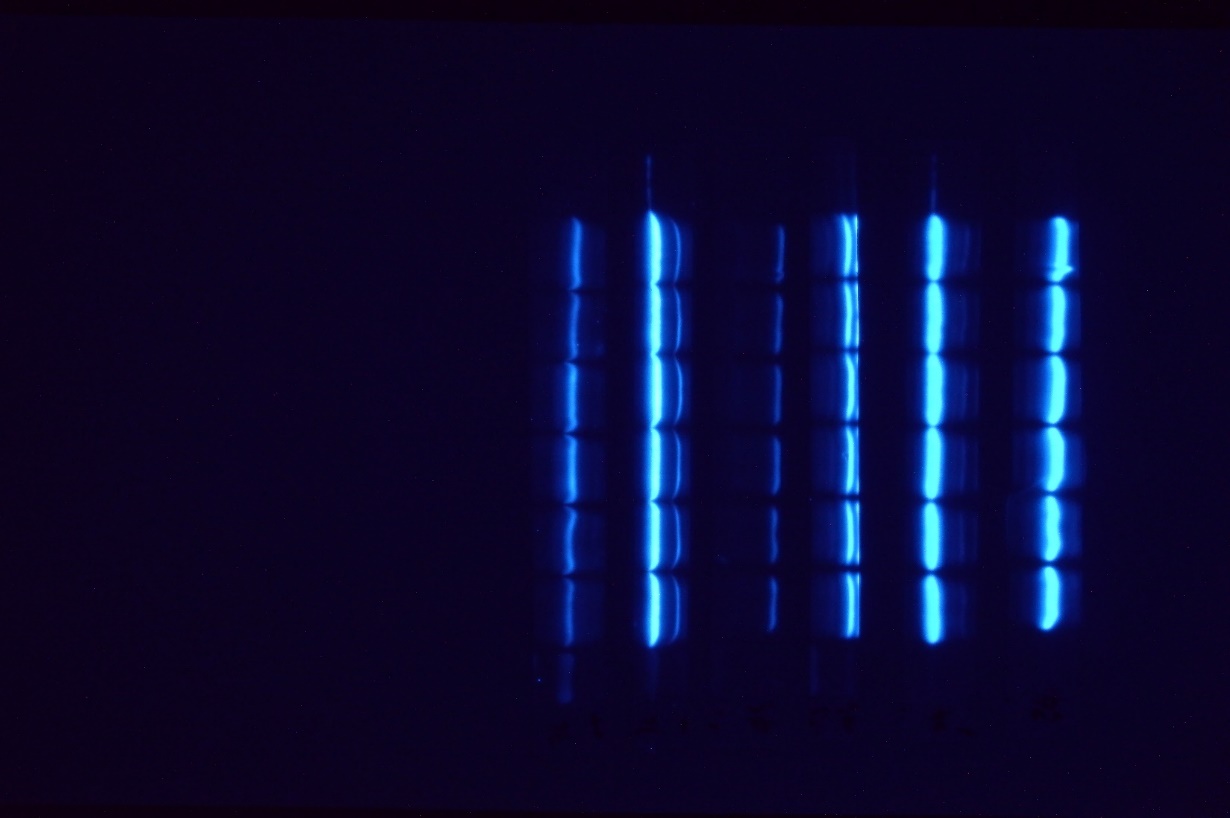


**p-NF-κB 65 kDa**





**NF-κB 65 kDa**

**p-ERK 42,44 kDa**

**ERK 42,44 kDa**

**GAPDH 37 kDa**

**p-p38 40 kDa**





**p38 40 kDa**


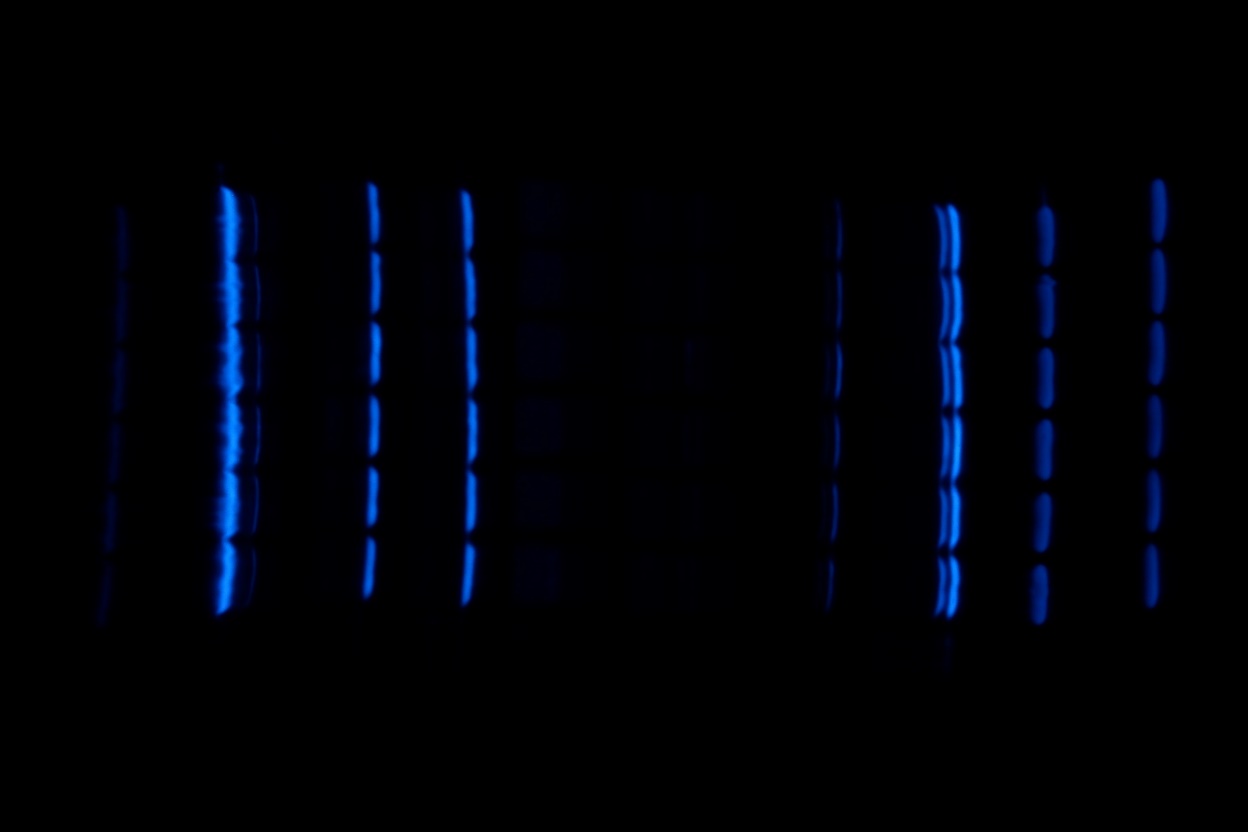


**GAPDH 37 kDa**

**GAPDH 37 kDa**




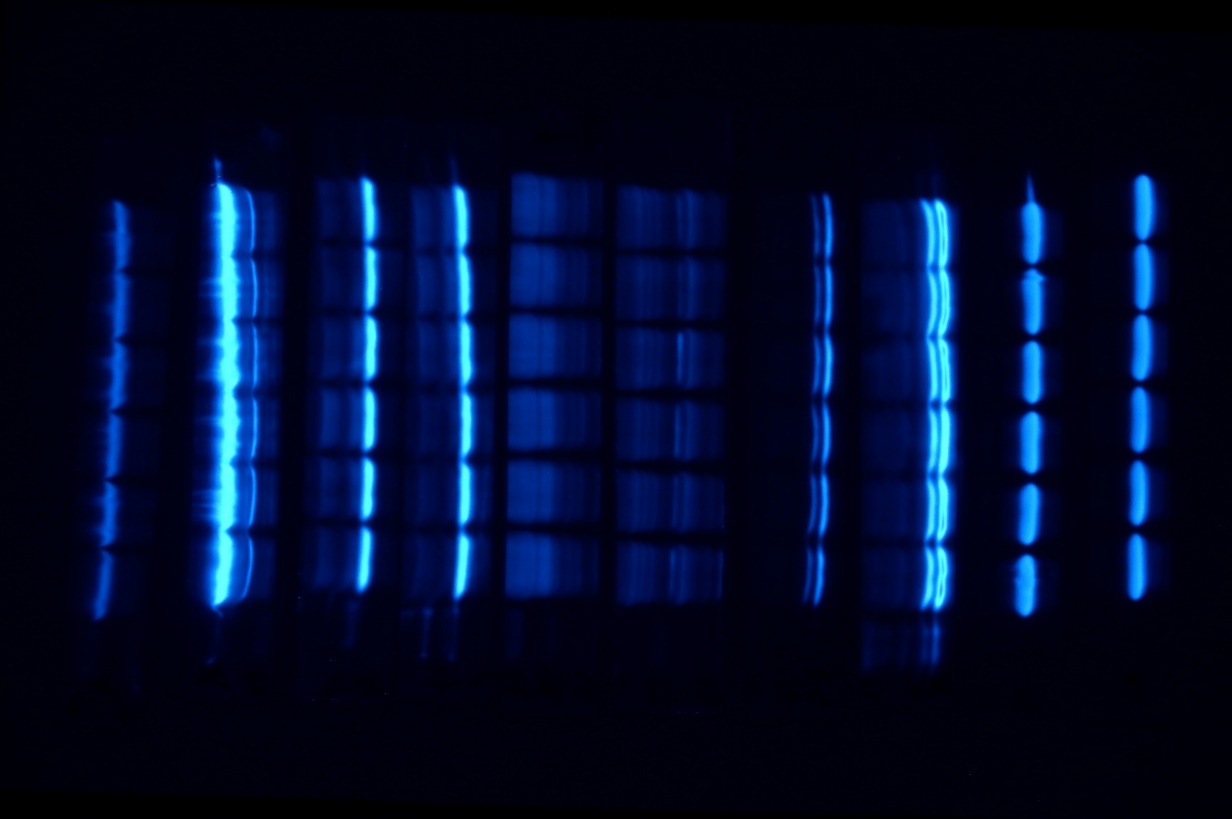


**p-ERK 42,44 kDa**

**ERK 42,44 kDa**

**GAPDH 37 kDa**

**GAPDH 37 kDa**





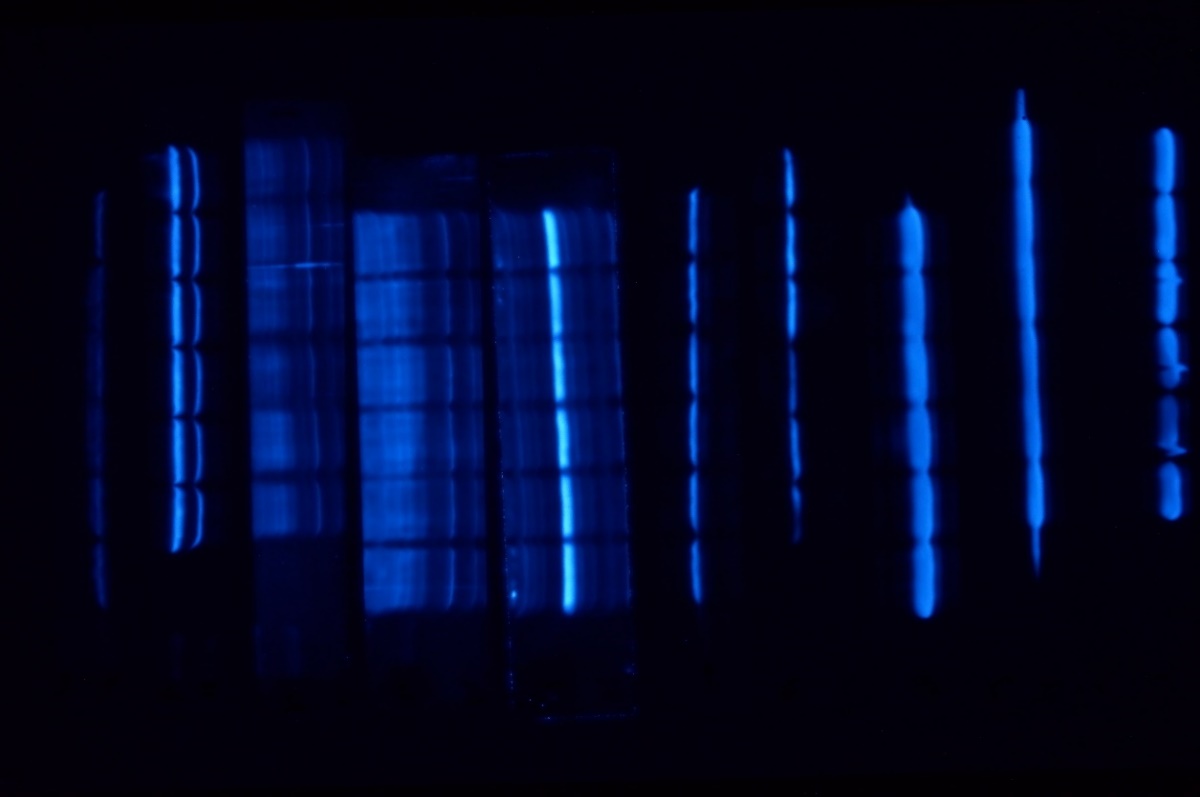


**p-JNK 46,54 kDa**

**JNK 46,54 kDa**

**GAPDH 37 kDa**


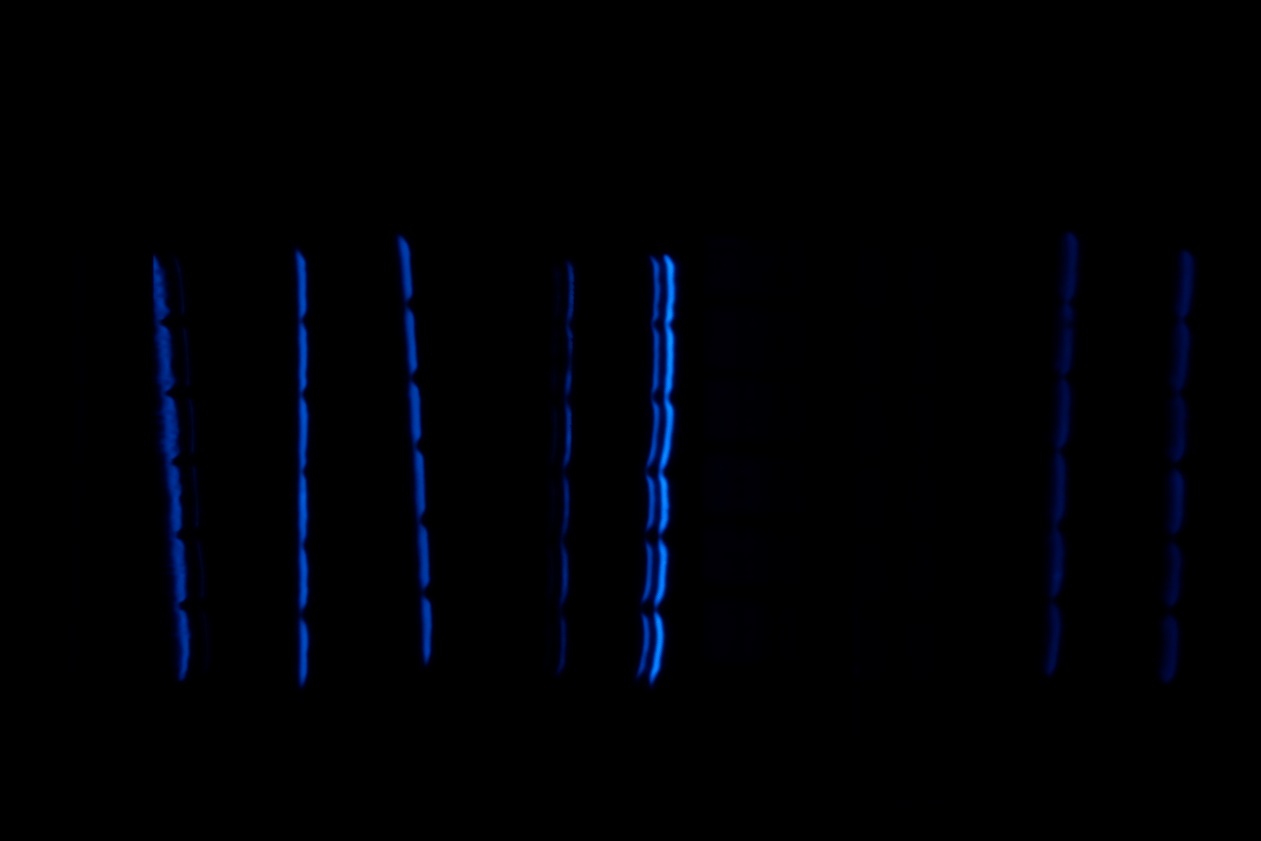

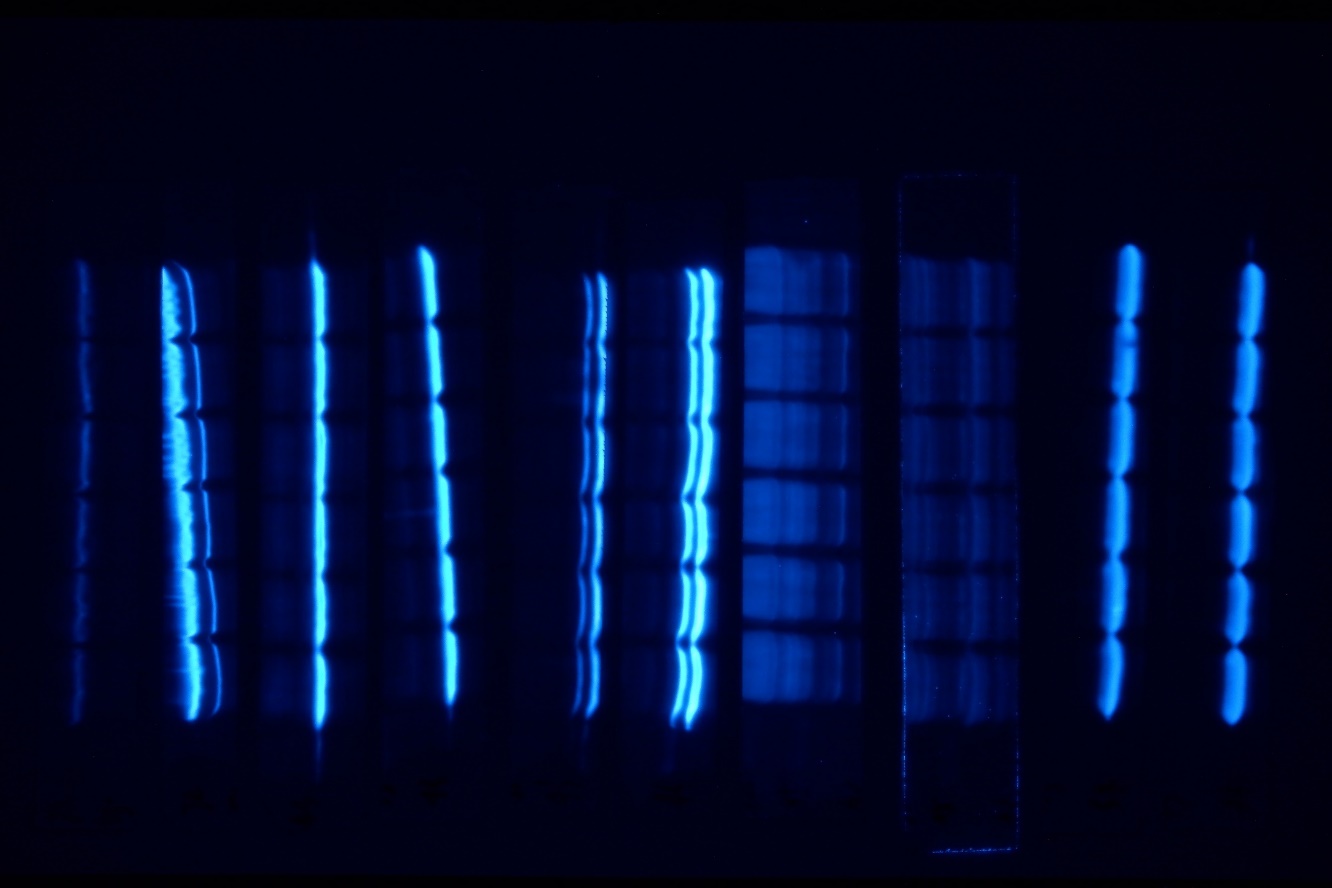


**p38 43 kDa**

**p-p38 43 kDa**

**GAPDH 37 kDa**

**JNK 46, 54 kDa**

**JNK 46, 54 kDa**

**ERK 42,44 kDa**

**p-ERK 42,44 kDa**

**p-NF-κB 65 kDa**

**NF-κB 65 kDa**
